# Long-range formation of the Bicoid gradient requires multiple dynamic modes that spatially vary across the embryo

**DOI:** 10.1242/dev.202128

**Published:** 2024-02-12

**Authors:** Thamarailingam Athilingam, Ashwin V. S. Nelanuthala, Catriona Breen, Narain Karedla, Marco Fritzsche, Thorsten Wohland, Timothy E. Saunders

**Affiliations:** ^1^ Warwick Medical School, University of Warwick, Coventry CV4 7AL, UK; ^2^ Mechanobiology Institute, National University of Singapore, Singapore 117411; ^3^ Department of Biological Sciences and Centre for Bioimaging Sciences, National University of Singapore, Singapore 117558; ^4^ Cornell University, Ithaca, NY 14850, USA; ^5^ Kennedy Institute of Rheumatology, University of Oxford, Oxford, OX3 7LF, UK; ^6^ Department of Chemistry, National University of Singapore, Singapore 117558

**Keywords:** Fluorescence correlation spectroscopy, Bicoid, *Drosophila* embryo, Morphogens, Diffusion

## Abstract

Morphogen gradients provide essential positional information to gene networks through their spatially heterogeneous distribution, yet how they form is still hotly contested, with multiple models proposed for different systems. Here, we focus on the transcription factor Bicoid (Bcd), a morphogen that forms an exponential gradient across the anterior-posterior (AP) axis of the early *Drosophila* embryo. Using fluorescence correlation spectroscopy we find there are spatial differences in Bcd diffusivity along the AP axis, with Bcd diffusing more rapidly in the posterior. We establish that such spatially varying differences in Bcd dynamics are sufficient to explain how Bcd can have a steep exponential gradient in the anterior half of the embryo and yet still have an observable fraction of Bcd near the posterior pole. In the nucleus, we demonstrate that Bcd dynamics are impacted by binding to DNA. Addition of the Bcd homeodomain to eGFP::NLS qualitatively replicates the Bcd concentration profile, suggesting this domain regulates Bcd dynamics. Our results reveal how a long-range gradient can form while retaining a steep profile through much of its range.

## Teaser

**Summary:** Morphogen dynamics can have spatial dependence, e.g. in the diffusivity, that can impact gradient extent and the speed of formation.

## INTRODUCTION

Morphogens are molecules that provide crucial spatial and temporal information to cells during development (Briscoe and Small, 2015; Wartlick et al., 2009). Knowing how morphogen gradients form and on what time scales is essential for understanding how information can be precisely decoded (Gregor et al., 2007b; Huang et al., 2017; Kicheva et al., 2007; Yu et al., 2009). Despite intensive study, the underlying dynamics of morphogen gradient formation remain controversial (Huang and Saunders, 2020; Kerszberg and Wolpert, 1998; Kornberg, 2014; Stapornwongkul and Vincent, 2021). A longstanding model is that morphogen gradients are formed by localised synthesis and diffusive processes combined with protein degradation or trapping (the SDD model) (Driever and Nüsslein-Volhard, 1988; Gregor et al., 2007b; Grimm et al., 2010; Yu et al., 2009). Even for systems that appear to be driven by diffusion, the SDD model is only an approximation and often requires adaption to system specifics (Gregor et al., 2007b; Yu et al., 2009).

Alternative hypotheses for generating a long-range gradient include distributed sources of morphogen (Ali-Murthy and Kornberg, 2016; Spirov et al., 2009) and long-range transport via cytonemes (Roy et al., 2014; Stanganello et al., 2015). For example, competing models have been proposed to explain whether Decapentaplegic forms through long-range diffusion, cytoneme-mediated transport or endocytic recycling (Romanova-Michaelides et al., 2022; Roy et al., 2014; Zhou et al., 2012). The shape of the morphogen profile can be adjusted by the mode of degradation (Eldar et al., 2003; Müller et al., 2013; Yu et al., 2009), which may make the gradient more robust to variation in morphogen protein levels (Eldar et al., 2003; He et al., 2010; Saunders and Howard, 2009). The morphogen profile can also be modulated through receptor binding (Stapornwongkul and Vincent, 2021; Veerapathiran et al., 2020).

A range of quantitative techniques have been used to measure morphogen dynamics *in vivo.* These include fluorescence correlation spectroscopy (FCS) (Fradin, 2017; Krieger et al., 2015; Wang et al., 2016; Zhou et al., 2012), fluorescence recovery after photobleaching (FRAP) (Gregor et al., 2007b), single molecule tracking (Kuhn et al., 2022; Mir et al., 2018) and protein lifetime measurements (Donà et al., 2013; Durrieu et al., 2018; Khmelinskii et al., 2012). FRAP and protein lifetime measurements provide insight into the longer time dynamics of the system. Essentially, they average out sub-second processes, giving a measure of the effective dynamic parameters across the system. FCS and single molecule imaging have the advantage of measuring the local fast dynamics, but often do not provide information about longer time and spatial processes. See Huang and Saunders (2020) and Müller et al. (2013) for an extended discussion on these points. These differences have led to conflict in the measured dynamic parameters for morphogens. For example, the reported diffusion constant for eGFP::Bcd can vary from ∼1 µm^2^/s (FRAP) (Castle et al., 2011) to 7 µm^2^/s (FCS) (Abu-Arish et al., 2010). Although theoretical work has looked to integrate these measurements, taking account of the different time scales measured (Sigaut et al., 2014), there remains significant contention over the Bcd dynamics. Similar issues are pertinent in other morphogen systems, such as Nodal (Lord et al., 2021; Müller et al., 2012; Wang et al., 2016) and Wingless (Stapornwongkul et al., 2020; Stapornwongkul and Vincent, 2021). Finally, analysis of morphogen dynamics has typically focused on a fluorescently tagged version of the wild-type morphogen protein. There is a lack of quantitative data on how morphogen dynamics are altered when protein domains, e.g. DNA or receptor binding motifs, are perturbed. Despite intense study over the past 20 years, it remains a major challenge to dissect the multiple time and spatial scales that underlie morphogen dynamics, and the mechanisms that shape the gradient; such knowledge is essential for understanding of how morphogens gradients form *in vivo* (Stapornwongkul and Vincent, 2021)*.*

Here, we take advantage of FCS combined with new Bcd mutant lines tagged with eGFP for live imaging to dissect the dynamics of Bcd with unprecedented precision. Using this, we reveal, with high accuracy, the dynamic modes that generate the Bcd gradient. We measure the Bcd dynamics at the anterior and posterior region of the embryo through nuclear cycles (n.c.) 12-14. We demonstrate that Bcd dynamics do not substantially change over n.c. 12-14, contrary to previous claims (Little et al., 2011). A two-component fit to the FCS curves is substantially better than a one-component fit in both the cytoplasm and nucleus; this implies two (‘slow’ and ‘fast’) dynamic modes in each region. The dynamics of the slower mode correspond closely to measured Bcd dynamics from FRAP. We show that the effective diffusion coefficient varies across the embryo, with it increasing towards the posterior. These results reveal that Bcd gradient formation is more complicated than previously considered. Implementing such spatial variation within a reaction-diffusion model, we show that the multiple dynamic modes can generate a long-range yet steep gradient across a large distance in a relatively short time. Finally, we explored eGFP::Bcd dynamics in a range of mutants with disrupted Bcd-binding capacity. Loss of DNA binding increases the fraction of eGFP::Bcd in the fast dynamic mode within the nucleus. In the cytoplasm, we demonstrate that the Bcd homeodomain plays a role in regulating Bcd diffusivity. Combining the Bcd homeodomain with an eGFP::NLS can reproduce the dynamics and gradient shape of the eGFP::Bcd gradient. Overall, we provide an improved version of the SDD model – involving spatially dependent dynamics – for the formation of the Bcd gradient, and we demonstrate that the Bcd dynamics are sensitive to a range of perturbations to binding elements with the protein.

## RESULTS

### Bicoid has multiple dynamic modes and these do not vary across nuclear cycles12 to 14

**Fig. 1.**
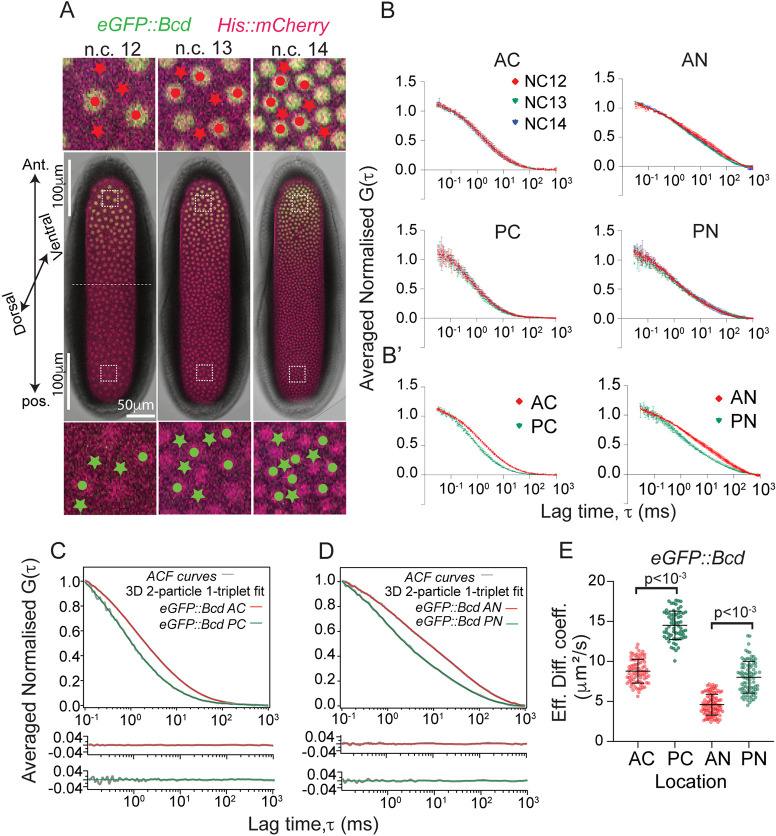
**Spatio-temporal dynamics of eGFP::Bcd in the early *Drosophila* embryo.** (A) *Drosophila* blastoderm showing the interphase periods of nuclear cycles (n.c.) 12, 13 and 14. Nuclei (mCherry::His2Aν, magenta) and eGFP::Bcd (green). Circles and stars indicate cytoplasmic and nuclear regions, respectively, where FCS measurements are carried out in the anterior (red) and posterior (green). The measurements are carried out within 100 µm of the anterior and posterior end of the embryo. The broken lines indicate the midline of the embryo. (B) Qualitative comparison of normalised and averaged autocorrelation (ACF) curves with mean and s.d. of eGFP::Bcd in the cytoplasmic and nuclear compartments of the n.c. 12, 13 and 14 interphases. The anterior and posterior domains are shown in the upper and lower panels, respectively. Lag times from 0.1 ms to 1 s are shown. (B′) Superimposed cytoplasmic (left) and nuclear ACF (right) curves from the anterior (red) and posterior (green). (C,D) Comparison of ACF curves (grey) with residues fitted with 3D 2-particle 1-triplet diffusion model. The comparisons are shown from 0.1 ms to 1 s lag times for visual clarity. Red and green fits correspond to anterior and posterior, respectively. (E) Scatter plot comparing the effective diffusion coefficients in different locations within the embryo: anterior cytoplasm (AC), posterior cytoplasm (PC), anterior nuclei (AN) and posterior nuclei (PN). For each condition, multiple measurements are taken from between three and five embryos in n.c. 12 to n.c. 14 (see Tables S2 and S3). Significance was calculated using a two-sided permutation test (Ho et al., 2019). *P*<0.001 indicates that diffusion values differ between anterior and posterior domains.

Previously, confocal FCS was used to measure the diffusion coefficients of eGFP::Bcd in the anterior cytoplasm (Abu-Arish et al., 2010) and anterior nuclei (Porcher et al., 2010). These measurements were limited in their position and timing within the embryo. We revisited these results, expanding the FCS measurements to both the anterior and posterior regions of the embryo during the interphases of n.c. 12, 13 and 14 (Fig. 1A,B, Fig. S1A-B). Owing to embryo curvature, our measurements were close to, but not at, the embryo poles on the dorsal surface (dashed boxes, Fig. 1A). Our FCS autocorrelation curves were highly reproducible between embryos both in the anterior domain and the posterior domain, where the brightness is very low (Fig. S1C). We co-imaged our eGFP::Bcd with H2Aν::mCherry to ensure that we precisely measure in the cytoplasmic and nuclear regions during the interphase stages of nuclear cycles (n.c.) 12-14.

Comparing the normalised FCS autocorrelation curves of cytoplasm and nuclei in n.c. 12-14 (Fig. 1B, Fig. S1C), we saw no clear differences in the curves within or between cycles during interphase. These results reveal that the dynamics of eGFP::Bcd are relatively stable through n.c. 12-14, both at the anterior and posterior locations of the embryo. This contrasts with previous claims based on imaging the Bcd profile within nuclei, which predicted a decrease in Bcd diffusion in later stages: D<1 µm^2^ s^−1^ (Little et al., 2011). We did not explore the dynamics during mitosis with point FCS because the nuclei (chromatin) move rapidly during this phase.

We next fitted dynamic models to the averaged autocorrelation curves (Fig. 1C,D). We found that a one-component model of diffusion is insufficient (Fig. S1D,E, Table S1), but a two-component diffusive model (with a fast- and slow-diffusing population) fitted the data well in all cases (Fig. 1C,D), consistent with previous FCS measurements (Abu-Arish et al., 2010; Porcher et al., 2010). We define the effective diffusion coefficient as *D*_*eff*_=*f*_*fast*_*D*_*fast*_+*f*_*slow*_*D*_*slow*_, where *D*_*fast*_ and *f*_*fast*_ represent the diffusion coefficient and fraction for the fast dynamic mode (and similarly for the slow dynamic mode), with *f*_*fast*_+*f*_*slow*_=1. We found the effective diffusion coefficients of eGFP::Bcd in the anterior cytoplasm and nuclei were 7-9 µm^2^ s^−1^ and 4-6 µm^2^ s^−1^, respectively (Fig. 1E), consistent with previous observations (Abu-Arish et al., 2010; Porcher et al., 2010). Therefore, we are confident our results are reproducible and reflect accurate measurements of the eGFP::Bcd interphase dynamics.

### Bicoid dynamics are spatially dependent in both the nuclei and cytoplasm

We analysed the effective diffusion coefficient in different regions of the embryo. Strikingly, for both nuclear and cytoplasmic eGFP::Bcd, the effective diffusivity was larger in the posterior of the embryo compared with the anterior (Fig. 1E). In the posterior, the effective eGFP::Bcd diffusion coefficient in the cytoplasm and nuclei ranged from 12-15 µm^2^ s^−1^ and 7-10 µm^2^ s^−1^, respectively (Tables S2 and S3), a ∼1.7 fold increase compared with the anterior (Fig. S1F). We confirmed this result even if each n.c. was analysed separately (Fig. S1G).

**Fig. 2.**
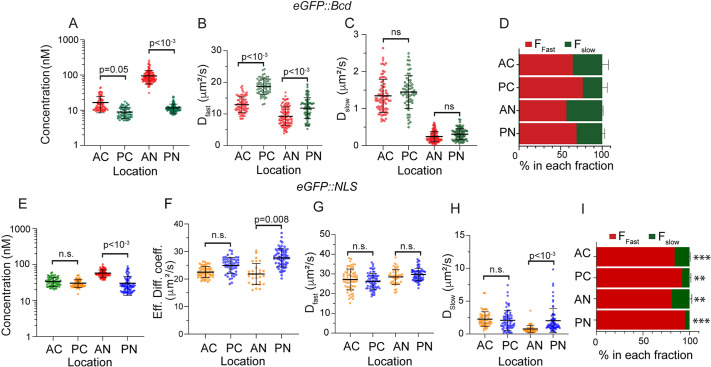
**Spatial dependence in Bcd but not NLS dynamics.** (A-C) Scatter plot comparing the concentration (A), fast (B) and slow (C) diffusion components of eGFP::Bcd at different locations within the embryo. For each condition, multiple measurements are taken from between three and five embryos in n.c. 12 to n.c. 14. (D) Fraction of Bcd in fast and slow dynamic form at different locations within the embryo. (E) Effective diffusion coefficient plotted against approximated Bcd concentration. A,E are on log scale for concentration. E,G-I is same as for A-D but for eGFP::NLS. F corresponds to Fig. 1E. The significance of data in scatter plots (A-C,E-H) and bar plots (D,I) was calculated using a two-sided permutation test (Ho et al., 2019). *P*<0.001, *P*<0.01 and n.s. (not significant) indicate statistical variations in the observed concentration and diffusion values. **P<0.01, ***P<0.001 indicate statistical comparison of the fast and slow component fractions of corresponding regions between eGFP::Bcd (D) and eGFP::NLS (I).

Our FCS measurements can be used to estimate the local eGFP::Bcd concentration (Fig. 2A) (see Materials and Methods). The measured effective diffusion seems inversely correlated to eGFP::Bcd concentration (compare Fig. 1E with Fig. 2A). Our estimation of concentration uses G(0), which is inversely proportional to the number of molecules in the FCS curves. In the anterior, this estimation is reliable as there is high signal-to-noise ratio. However, in the posterior, the relative noise is higher, and so our estimate of concentration is more uncertain (Fig. S1H, see Materials and Methods). Exploring the different dynamic modes for eGFP::Bcd (Fig. 2B-D), we see that in the cytoplasm the slow dynamic mode was similar across the embryo: ∼ 1 μm^2^ s^−1^ (Fig. 2C). The fast mode showed an increased diffusivity from *D*_*fast*_ (*anterior*)= 13.0±2.6 μm^2^ s^−1^ to *D*_*fast*_ (*posterior*)=18.8±2.5 μm^2^ s^−1^ (Fig. 2B, Table S2). In the nuclei, eGFP::Bcd showed a significant, although small, increase in diffusivity in the fast dynamic mode from anterior to posterior regions (9-12 µm^2^ s^−1^ respectively), with the slow component remaining unchanged (Fig. 2B,C, Table S3).

We next asked whether the relative fractions of slow and fast components change along the embryo AP axis. In the anterior cytoplasm, the faster diffusing species comprised *f*_*fast*_=65±8%, whereas in the posterior this increased to *f*_*fast*_=78±6% (Fig. 2D, Table S2). We further tested the accuracy of the measured *D*_*fast*_ and *D*_*slow*_ values by refitting our two-particle model with the photophysical parameters (triplet states) (Fig. S2). Although the slow cytoplasmic dynamic mode was similar in both anterior and posterior of the embryo, the effective diffusion increased towards the posterior because (1) the fast dynamic mode was quicker and (2) the fraction of the eGFP::Bcd in the fast mode was larger. Likewise, in the nucleus the fraction of eGFP::Bcd in the faster dynamic mode increased towards the posterior, thereby increasing the effective diffusion coefficient (Fig. 2D, Table S3). In addition, we verified the obtained *D*_*slow*_ values of confocal FCS through SPIM based FCS that captured the spatial dynamics of eGFP::Bcd simultaneously in the anterior nuclei, cytoplasm and nuclear periphery (Fig. S3).

We performed a similar analysis for eGFP::NLS, driven by the Bcd promoter region, thus forming an anterior-posterior gradient (see Materials and Methods, Fig. S4A). The results again fitted with a two-component diffusive model. The majority of the cytoplasmic eGFP::NLS was in the faster diffusive mode (Fig. 2E-H, Fig. S4, Tables S2 and S3). There was a small difference in the eGFP::NLS dynamics in the nuclei between anterior and posterior ends, possibly owing to crowding/non-specific interaction effects in the anterior where protein number is higher (Fig. 2E). Supporting this, anterior nuclei show a small but appreciable fraction (∼20%) of eGFP::NLS in a slower mode (Fig. 2G,H, Table S3). eGFP::NLS diffusion is more rapid than eGFP::Bcd, with 80-95% existing in the faster diffusive mode. This is consistent with eGFP::NLS only weakly interacting with other components (e.g. itself or DNA binding and cytoplasmic elements).

In summary, the dynamics of eGFP::Bcd varies across the embryo, both in terms of the magnitude of the diffusivity and the fraction of slow- and fast-diffusing eGFP::Bcd. This means that the classic SDD model needs to be revisited as the dynamics are dependent on the spatial location and/or the local morphogen concentration within the embryo.

### Formation of the Bcd gradient with multiple dynamic modes

**Fig. 3.**
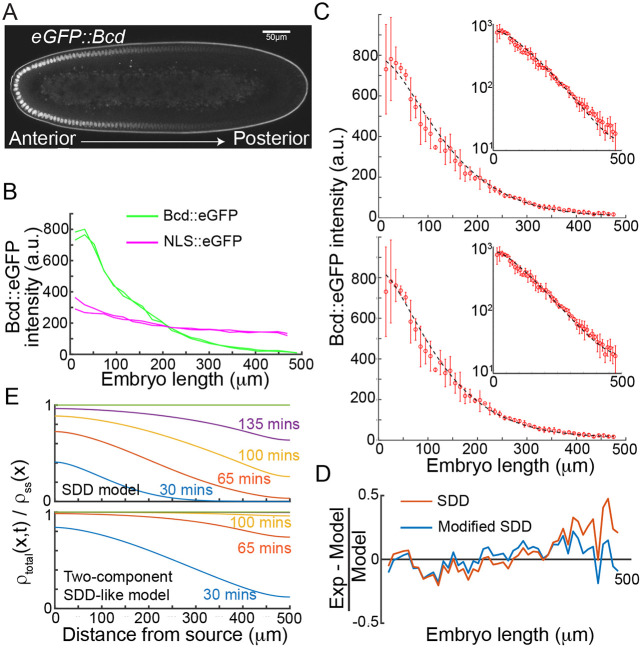
**Concentration-dependent parameters within the SDD model can replicate the observed Bcd dynamics and gradient.** (A) Embryo expressing eGFP::Bcd, showing the gradient from anterior (left) to posterior (right). (B) Two example profiles for Bcd::eGFP (green) and NLS::eGFP (magenta) normalised from Histone mCherry background fluorescence. (C) Top: SDD model prediction for the formation of the Bcd gradient (dashed black line) compared with experimental profiles (red circles, mean±s.d., *n*=3). Inset shows the same on a log intensity scale. Bottom: the same as above but with a fit from the modified 2-component SDD model (see supplementary Materials and Methods). (D) Relative fitting precision of the two models from (C) along the embryo AP axis. (E) Evolution of gradient formation for the SDD model (top) and modified 2-component SDD model (bottom). *Y*-axis is the relative difference from steady-state at each position (value of 1 indicates being in a steady-state).

Given distinct dynamic modes for Bcd movement exist across the embryo, we considered the effects of these on long-range gradient formation (Fig. 3A). We quantified the gradient of Bcd::eGFP and NLS::eGFP in our embryos (Fig. 3B and Materials and Methods). We see that the NLS::eGFP is noticeably shallower than the Bcd::eGFP gradient, qualitatively consistent with our measured diffusion coefficients above.

The SDD model assumes a single effective diffusion coefficient across the embryo. It can fit the data well for <300 µm along the AP-axis (see Materials and Methods), but the fit quality reduces towards the posterior (Fig. 3C), consistently underestimating the posterior concentration. To develop an improved SDD-like model of Bcd gradient formation, we need to consider: (1) Bcd has multiple dynamic modes that vary across the embryo; and (2) the observed Bcd gradient is exponential with a decay length λ=80-100 µm across most of the embryo. There are (at least) four effective modes of Bcd movement: (1) fast cytoplasmic fraction; (2) slow cytoplasmic fraction; (3) fast nuclear localised fraction; and (4) slow nuclear localised fraction. We know the fractions in each population in the anterior and posterior of the embryo (Fig. 2D). As our focus here is on the long-range establishment of the Bcd morphogen gradient, we considered the nuclear-bound fraction to be stationary, and only considered the cytoplasmic component. This assumption is supported by work showing that the Bcd gradient can form with substantially reduced Bcd nuclear import (Grimm and Wieschaus, 2010). To implement the spatially varying diffusion coefficient, we consider 
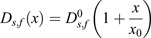
 for both slow and fast cytoplasmic components. It is likely that the Bcd diffusivity is really a function of concentration (discussed below), but we use this linear form for simplicity. To account for the changes in the fraction in the slow and fast forms across the embryo, we consider the rate of cytoplasmic Bcd transition from fast to slow forms (denoted by *β*) to behave as 
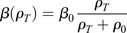
, where *ρ*_*T*_=*ρ*_*s*_+*ρ*_*f*_ is the total amount of Bcd at each position, *ρ*_*s*_ and *ρ*_*f*_ are the slow and fast forms of Bcd cytoplasmic concentration, respectively, and *β*_0_, *ρ*_0_ are constants. With this form, the transition rate from fast to slow forms is larger near the anterior pole.

Applying these assumptions leads to the coupled differential equations:

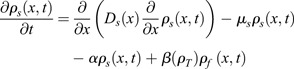
and

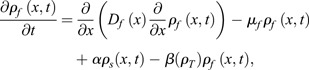
where *μ*_*s*,*f*_ denotes the degradation rate for the slow and fast forms, and *α* is the rate of slow-to-fast transition. For simplicity, we keep all parameters constant that are not explicitly highlighted as having a functional dependence. Implementing parameters based on our above and other *in vivo* measurements (Abu-Arish et al., 2010; Drocco et al., 2011; Durrieu et al., 2018), we can fit the n.c. 14 Bcd gradient as a function of position (Fig. 3C,D). In the supplementary Materials and Methods, we discuss other potential model variations and the resulting fit quality (Fig. S5, Table S7). The additional of fast and slow populations allows a more accurate fit to the Bcd profile (Fig. 3D), although this is arguably unsurprising given the increased parameter space.

Are there other advantages by having multiple dynamic modes? Given the large value for *D*_*fast*_, we postulated that the rate of gradient formation in the posterior will be more rapid in the two-component model. Comparing the SDD model with our modified two-component version (Fig. 3E), we see that the morphogen reaches the posterior more rapidly with two dynamic components. We note that in the posterior the absolute concentration differences between the models is not large, but there is a substantial change in the relative concentration differences from steady-state as a function of time.

We can estimate the time taken for a molecule to move from the anterior to posterior poles. The time taken for a molecule to diffuse an average distance x_rms_ is given by 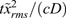, where the constant *c* depends on the system dimensionality. Taking *D*≈7 μm^2^ s^−1^, *x_rms_*≈450 µm and *c*=4 (representing effectively 2D diffusion near the surface) gives *t*≈2 h. This is two to four times the Bicoid lifetime – i.e. fewer than 10% of the molecules that may diffuse a sufficient distance are also likely not to degrade within that time. In the case of *D*≈18 μm^2^ s^−1^, this time is more than halved.

From this analysis, we see three important points: (1) having slow and fast forms of Bcd leads to more rapid gradient formation at larger distances (Fig. 3E); (2) the increased fraction in the fast form results in increased concentration in the posterior compared with a simple SDD model (Fig. 3D), consistent with experimental observation of Bcd in the most posterior (Mir et al., 2017); (3) even with the multiple species and varying diffusion, the gradient is still exponential across a large extent, consistent with experimental observations (Fig. 3B, see Materials and Methods for details of gradient quantification and error estimation) (Gregor et al., 2007b).

How do these different dynamic modes arise? They are not a simple consequence of spatial differences across the embryo, as the eGFP::NLS results show no such spatial dependence. In the next sections, we explore perturbations to binding elements of Bcd to dissect possible mechanisms driving the observed behaviour.

### Bicoid DNA binding determines the slow diffusion dynamics within the nucleus

Given our above observations, we investigated the possible mechanisms regulating Bcd diffusivity. Bcd is a transcription factor known to cooperatively bind to DNA (Burz et al., 1998; Lebrecht et al., 2005). Furthermore, Bcd binds to *caudal* mRNA in the BRE (Bicoid response element) of the *caudal* 3′UTR, repressing its translation in the cytoplasm (Niessing et al., 2002, 1999, 2000). Therefore, we hypothesised that direct DNA/RNA binding impacts Bcd dynamics.

**Fig. 4.**
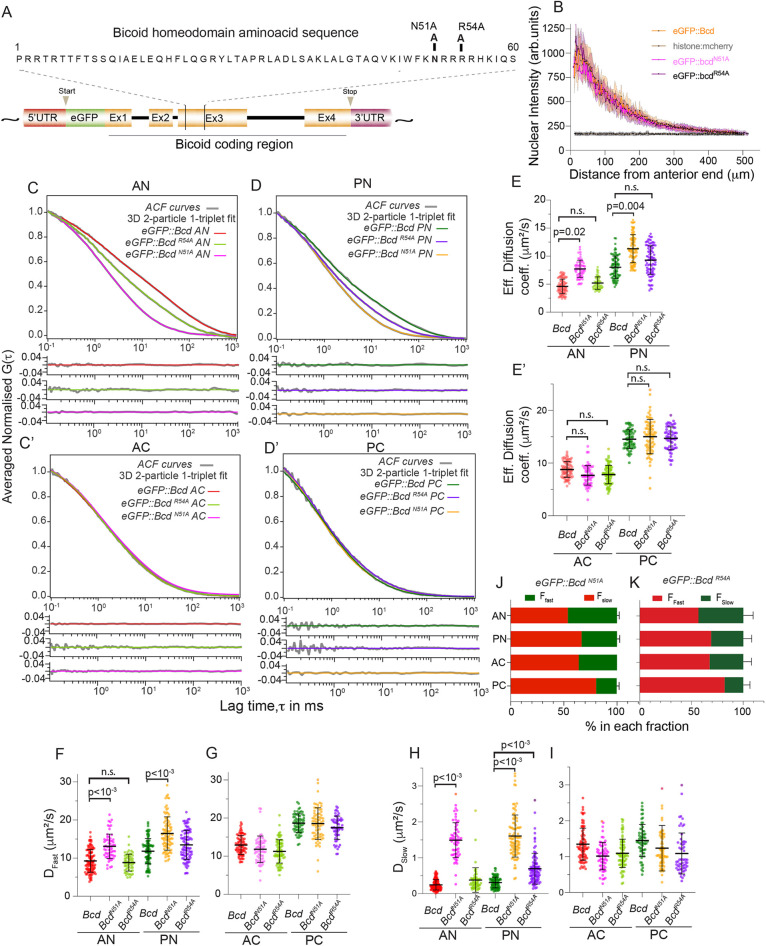
**Bcd homeodomain alters nuclear Bcd dynamics.** (A) Schematic of *Drosophila bcd* genomic region. A 60 amino acid region constituting the *bcd* homeodomain is expanded. Amino acid conversion at position 51 from asparagine to alanine (N51A) and at position 54 from arginine to alanine (R54A) is displayed. (B) Gradient profile to embryo length plot of the midsagittal sections of eGFP::bcd^N51A^, eGFP::bcd^R54A^, eGFP::bcd and mCherry::His2Aν. (C-D′) Comparison of averaged normalised ACF curves (grey) of eGFP::Bcd, eGFP::Bcd^N51A^ and eGFP::Bcd^R54A^ with residues fitted with 3D 2-particle 1-triplet diffusion model. Red and green fits correspond to nuclear and cytoplasmic regions in anterior and posterior regions, respectively for eGFP:Bcd. Magenta and orange fits correspond to eGFP::Bcd^N51A^. Light green and purple fits correspond to eGFP::Bcd^R54A^. (E,E′) Comparison of the effective diffusion coefficients of eGFP::Bcd, eGFP:Bcd^R54A^ and eGFP::Bcd^N51A^ in the nucleus (E) and cytoplasm (E′). (F-I) Scatter plots comparing D_fast_ (F,G) and D_slow_ (H,I) of eGFP::Bcd^N51A^ and eGFP::Bcd^R54A^ at different locations within the embryo. Anterior cytoplasm (AC), posterior cytoplasm (PC), anterior nuclei (AN) and posterior nuclei (PN). (J,K) Bar plots indicating fractions (%) of fast- and slow-diffusing eGFP::Bcd^N51A^ (J) and eGFP::Bcd^R54A^ (K) molecules in the corresponding embryo compartments. The significance of scatter plots was tested using a two-sided permutation test (Ho et al., 2019). *P*<0.0001, *P*<0.001, *P*<0.05 and n.s. (not significant) indicate statistical comparison of observed diffusion values. All error bars indicate±1 s.d.

The Bcd homeodomain mutation *bcd^N51A^* leads to loss of downstream target expression (e.g. *hunchback*), posited due to disruption of Bcd DNA-binding efficacy (Niessing et al., 2000). It also has a reported role in reducing the repression of *caudal* expression by Bcd in the cytoplasm. Another homeodomain mutation, *bcd^R54A^*, is also reported to de-repress caudal expression but it has normal *hunchback* expression (Niessing et al., 2000). To test whether these mutations in Bcd alter the protein dynamics, we generated eGFP::Bcd^N51A^ and eGFP::Bcd^R54A^ (Fig. 4A). These embryos show a clear anterior-to-posterior gradient of Bcd (Fig. 4B).

We performed FCS on eGFP::Bcd^N51A^ embryos at different locations within n.c. 12-14 embryos (Fig. S6, Table S4). In the nucleus, the normalised autocorrelation curves for eGFP::Bcd^N51A^ embryos were clearly different from eGFP::Bcd embryos, with faster dynamics (Fig. 4C-E). However, the dynamics in the cytoplasm were not significantly altered (Fig. 4C′-E′). The effective diffusion coefficients further reveal that the diffusion of eGFP::Bcd^N51A^ in the anterior nuclei was 7-9 μm^2^ s^−1^ and in the posterior it was 9-13 μm^2^ s^−1^ (Fig. 4E, Table S4).

In the nucleus, both the fast and slow modes in eGFP::Bcd^N51A^ embryos show increased diffusivity compared with eGFP::Bcd embryos (Fig. 4F-I). Although the increase in the slow mode was expected due to the loss of Bcd binding to the DNA, the reason for the change in the fast mode is less clear. These effects are apparent in both the anterior and posterior of the embryo (Fig. S6). As with eGFP::Bcd, the slow mode diffusivity in eGFP::Bcd^N51A^ embryos was similar across the embryo (Fig. 4H), with the diffusion coefficient of the fast component increasing towards the posterior (Fig. 4F). The relative fractions of eGFP::Bcd^N51A^ in the slow and fast modes also changed from anterior to posterior (Fig. 4J), with a larger fraction of eGFP::Bcd^N51A^ being in the faster diffusive mode within the posterior nuclei. These results demonstrate that homeodomain function influences the Bcd diffusion dynamics, at least in the nuclei.

The dynamics of eGFP:Bcd^R54A^ embryos were more similar to eGFP::Bcd (Fig. 4F-K, Fig. S7). There is little difference in the measurements for the diffusion coefficients in nuclei. This is consistent with the R54A mutation not affecting hunchback expression (Niessing et al., 2000). There is a decrease in *D_slow_* in the posterior, suggesting that the disrupted interaction with *caudal* may be altering posterior Bcd dynamics.

In addition to the homeodomain, the YIRPYL motif and the PEST domain are involved in Bcd-mediated *caudal* mRNA repression. Point mutations in these *bcd* domains are known to abolish the caudal repression in the cytoplasm (Niessing et al., 2002, 1999). Conversions of tyrosine (Y) to alanine (A) and leucine (L) to arginine (R) in the YIRPYL motif (YIRPYL→AIRPYR) abolishes *caudal* repression in the cytoplasm by breaking the interaction of Bcd with the translation initiation factor, eIF4E, at the 5′cap *caudal* RNA (Niessing et al., 2002). Furthermore, replacing four threonine (T) and one serine (S) residues to five alanine (A) residues (Bcd^5aa^) in the PEST domain at positions 188, 193, 195, 197 and 200 abolishes *caudal* repression (Niessing et al., 1999). The above mutations may still have residual interacting elements of Bcd to *caudal* mRNA. To test the effect of removing all these binding elements on Bcd dynamics, we generated eGFP:Bcd multi-mutant (MM) line that harbours mutations in the PEST (Bcd^5aa^) domain along with homeodomain N51A and YIRPYL motif (AIRPYR) (Fig. S8A). We refer this line as eGFP::Bcd^MM^. FCS measurements of eGFP::Bcd^MM^ embryos in the anterior cytoplasm and nuclei revealed similar Bcd dynamics to eGFP::bcd^N51A^ embryos both in slower and faster Bcd dynamics (Fig. S8, Table S5).

Our results for the N51A, R54A and *bcd^MM^* alleles suggest that: (1) the Bcd homeodomain plays an important role in determining Bcd dynamics; (2) Bcd binding to *caudal* mRNA alone is insufficient to explain the Bcd cytoplasmic dynamics; and (3) there are likely other components (either within Bcd itself or other proteins) in the cytoplasm that affect Bcd dynamics at different Bcd concentrations.

### The Bcd homeodomain regulates protein dynamics in both the nucleus and cytoplasm

Given our above results, combined with the presence of putative cytoplasmic interaction sites within the Bcd homeodomain (Burz et al., 1998; Lebrecht et al., 2005; Ling et al., 2019; Ma et al., 1996; Niessing et al., 1999; Yuan et al., 1996), we hypothesised that the Bcd homeodomain alone (without the rest of the Bcd protein) may be sufficient to replicate, at least partially, the observed Bcd protein dynamics.

**Fig. 5.**
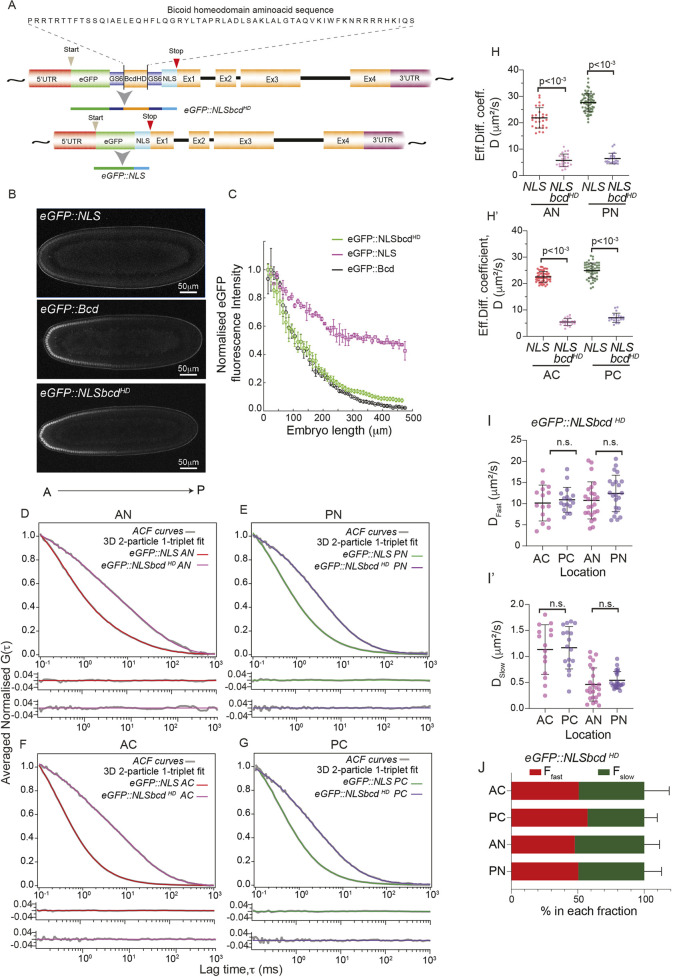
**Effects on dynamics of adding the *Bcd* homeodomain to NLS.** (A) Schematic of the construction of the eGFP::NLS and eGFP::NLSbcd^HD^ lines. The *bcd* homeodomain is inserted between eGFP and NLS flanked by GS6 linker to generate eGFP::NLSbcd^HD^. (B) Midsagittal sections of *Drosophila* embryos at n.c.14 expressing eGFP::NLS (top), eGFP::Bcd (middle) and eGFP::NLSbcd^HD^ (bottom). (C) Comparison of normalised gradient profiles between eGFP::NLS (magenta), eGFP::NLSbcd^HD^ (green) and eGFP::Bcd (black). Dashed lines indicate the standard deviation of gradient profiles. (D-G) Comparison of averaged normalised ACF curves (grey) of eGFP::NLS and eGFP::NLSbcd^HD^ with residues fitted with 3D 2-particle 1-triplet diffusion model. Red and green fits correspond to nuclear and cytoplasmic regions of anterior and posterior of eGFP::NLS, respectively. Magenta and violet fits correspond to eGFP::NLSbcd^HD^. (H,H′) Comparison of the effective diffusion coefficients of eGFP::NLS and eGFP::NLSbcd^HD^ for nuclei (H) and cytoplasm (H′). (I,I′) Scatter plots comparing D_fast_ (I) and D_slow_ (I′) for eGFP::NLSbcd^HD^. (J) Bar plots indicating fractions (%) of fast- and slow-diffusing eGFP::NLSbcd^HD^ molecules in the corresponding embryo compartments. *P*-values calculated using a two-sided permutation test (Ho et al., 2019). All error bars indicate±1 s.d.

To test this hypothesis, we fused the *bcd* homeodomain to eGFP::NLS, which we refer to as eGFP::NLSbcd^HD^ (Fig. 5A). We compared the gradient profiles of eGFP:NLS*,* eGFP:: NLSbcd^HD^ and eGFP::Bcd (Fig. 5B,C)*.* Consistent with our prediction, the gradient of eGFP::NLSbcd^HD^ was steeper than eGFP::NLS (Fig. 5C)*.* Remarkably, the eGFP::NLSbcd^HD^ concentration gradient closely matched the eGFP::Bcd profile (Fig. 5C), with only an increased concentration towards the posterior. This is consistent with the lifetime of eGFP::NLSbcd^HD^ being longer than eGFP::Bcd. This strongly suggests that the homeodomain interactions in the cytoplasm and nuclei are significant contributors to determining Bcd dynamics.

Next, we performed FCS of eGFP::NLSbcd^HD^ embryos to explore the dynamic modes (Fig. 5D-G, Fig. S9, Table S6). Interestingly, there were no anterior-posterior differences in the diffusion coefficients of eGFP::NLSbcd^HD^ embryos (Fig. 5H,H′, Fig. S9A″). This supports the conclusion that the anterior-posterior dynamic changes are due to interactions of the Bcd protein with itself or other elements, and not, for example, due to differences in the physical environment between the anterior and posterior. For eGFP::NLSbcd^HD^, the slow and fast populations represented ∼50% each at all positions within the embryos (Fig. 5J). There is ∼3.5- to 4.5-fold decrease in the effective diffusion coefficients of eGFP::NLS (Fig. 5H,H′) upon addition of the homeodomain to NLS encompassing ∼2.5-fold decrease in both D_fast_ and D_slow_ values (Fig. S9B-E). Furthermore, the fraction of eGFP::NLSbcd^HD^ in the slower form (*F_slow_*) significantly increased compared with eGFP::NLS (compare Fig. 2I and Fig. 5J). We note that D_slow_ in the nuclei and cytoplasm (0.5 µm^2^ s^−1^ and 1 µm^2^ s^−1^) are comparable between eGFP::NLSbcd^HD^ and eGFP:Bcd (0.2-0.3 µm^2^ s^−1^ and 1 µm^2^ s^−1^) embryos. This suggests that homeodomain binding affinities to nuclear DNA and cytoplasmic RNA or cytoplasmic elements are different, and this may regulate the slow Bcd diffusive mode. However, D_fast_ displays distinct differences between eGFP::NLSbcd^HD^ and eGFP:Bcd; the homeodomain is not sufficient to replicate all the Bcd protein dynamics.

## DISCUSSION

We have provided a detailed analysis of Bcd morphogen dynamics in both space and time. Our FCS measurements demonstrate that eGFP::Bcd has both highly motile and slower fractions, in both the nucleus and the cytoplasm. Crucially, these dynamics are spatially varying across the embryo. Given the changing fractions of the fast and slow populations in space, the interactions between the populations are likely non-linear, and dependent on the local eGFP::Bcd concentration. The resulting dynamics generate a gradient across the whole embryo. Although there is no known role for Bcd in the embryo posterior, it has recently been shown that Bcd influences boundary specification at 70% EL (around 350 µm from the anterior) (Singh et al., 2022).

FRAP measurements of eGFP::Bcd in the cytoplasm have reported an effective diffusion coefficient in the range of 1 μm^2^ s^−1^ (Abu-Arish et al., 2010; Castle et al., 2011). Our measurements of the cytoplasmic slow fraction (D_slow_∼1 µm^2^ s^−1^) are consistent with this. Second, distinct clusters of eGFP::Bcd have been observed in the embryo posterior (Mir et al., 2017). Even with an effective diffusion coefficient of 7 μm^2^ s^−1^, few molecules would be expected at the posterior given the estimated Bcd lifetime (30-50 min) (Fig. 3E). We show that eGFP::Bcd in its fastest form can move quickly 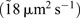, and the fraction of eGFP::Bcd in this form increases at lower concentrations. We note, however, that the relationship between Bcd diffusivity and concentration are correlative (also see Fig. S13E,F); we have not directly tested that altered Bcd concentration affects the Bcd diffusion coefficient. Given the fast population of Bcd, it is possible for a subpopulation of eGFP::Bcd to reach the posterior within the first 90 min of development, while the majority of eGFP::Bcd forms a steep concentration profile (Fig. 3D). This is consistent with theoretical predictions, which also postulated that Bcd may have spatially varying dynamics (Sigaut et al., 2014). The SDD model provides a good estimate of the Bcd gradient profile and the ‘average’ dynamics. However, it is inconsistent with Bcd puncta in the posterior pole, the speed of gradient formation in the posterior and our measurements of spatially varying diffusivity. Here, we present a modified SDD model, where the diffusion component is itself spatially varying. However, we emphasise that this model is phenomenological; it will be interesting to dissect further the specific mechanisms driving the non-linear dynamics we observe.

These results suggest that: (1) Bcd DNA binding plays an important role in determining Bcd dynamics within the nucleus and (2) the dynamics of Bcd within the nucleus are more complicated than a simple model of bound versus unbound Bcd (Fradin, 2017). This might point towards anomalous diffusion as the dominant diffusive mode (Höfling and Franosch, 2013), in which, instead of two distinct diffusion components, the diffusion coefficient is scale dependent. In fact, the anomaly parameter can provide a good empirical measure for changes in molecular interactions (Fradin, 2017). However, the anomalous and two-component fit cannot be easily differentiated, and the two-component model provides a simpler model and clearer interpretation of the changes due to binding. There may be other modes that are not considered, but our approach – given the excellent fit to the FCS profiles – appears to approximate well the underlying dynamics. We include binding/unbinding within our model. Our evidence suggests that the dynamics in the nucleus are driven by DNA binding/unbinding. Yet the dynamics are less clear in the cytoplasm and it is an assumption of our phenomenological 2-state model that Bcd can reversibly move between fast and slow cytoplasmic forms.

Bcd maintains a similar profile across multiple nuclear cycles (Gregor et al., 2007b). The presence of a rapidly diffusing pool can (at least partially) help to re-establish the gradient quickly after each division. It has also been proposed that Bcd can be produced throughout the embryo, without need for long-ranged diffusive movement (Spirov et al., 2009). Yet our results suggest that >50% of eGFP::Bcd exists in a rapidly diffusing form (*D*>5 μm^2^ s^−1^) that will ‘wash out’ a locally produced gradient. To summarise, our results provide a mechanism for Bcd to have both slow dynamics (as measured by FRAP) and rapid movement (measured in FCS), which set up the gradient across the embryo in a few hours, largely driven by hindered diffusion. A caveat to this conclusion is that previous analysis of Bcd::eGFP profiles suggested that Bcd dynamics become constrained in n.c. 13 and 14 (Little et al., 2011). Our results show that there still exists a dynamic pool of Bcd at these times. However, local barriers between nuclei – particularly during cellularisation – may impede long-range Bcd movement. To test this idea, we used SPIM-FCS to explore the local spatial distribution of diffusion of Bcd::eGFP around the nucleus (Fig. S3). We see a clear reduction in Bcd::eGFP diffusivity around the nuclear envelope, consistent with slow movement in/out of the nucleus during interphase. However, we note that this approach does not capture the fast diffusive mode (owing to limitations in camera speed).

We have also tried to dissect the specific interaction elements of Bcd that drive its dynamics. In the nucleus, the two populations can be largely (though not completely) explained by Bcd binding to DNA. We have shown in the cytoplasm that the ability to transform the eGFP::NLS gradient into one that qualitatively matches the eGFP::Bcd gradient through the addition of the Bcd homeodomain suggests that this region of Bcd is crucial in determining Bcd dynamics. Our eGFP::NLSbcd^HD^ construct does not show concentration/spatial dependence, suggesting that the homeodomain is not wholly sufficient to explain the Bcd dynamics. It is possible that Bcd interacts with cytoplasmic elements, including actin and microtubule structures (Cai et al., 2017), which alter its diffusivity. We also observe evidence for non-specific binding in the *bcd^N51A^* embryos and also in the NLS::eGFP nuclear anterior fraction. This is consistent with non-specific DNA interactions (Vukojević et al., 2010), but further work is required to dissect these interactions.

Such spatially varying dynamics have been hypothesised previously (Lipkow and Odde, 2008). The subcellular gradient of MEX-5 within the *C. elegans* embryo has spatially varying dynamics, due to interactions mediated by polarised distribution of PAR proteins (Griffin et al., 2011), and a recent study in *Xenopus* extracts has shown that cytoplasmic organisation can alter protein diffusivity (Huang et al., 2022). In single molecule tracking of Nodal, multiple dynamic modes have been observed (Kuhn et al., 2022). In our case, there are no known significant structural differences in the anterior and posterior ends of the embryo at this stage, nor are there gradients of polarity. Our results suggest that the Bcd homeodomain has a role in regulating the protein dynamics in the cytoplasm. Bcd binds to the BRE (Bicoid response element) in the *caudal* 3′UTR and it also binds to the 5′cap of the *caudal* mRNA through its PEST domain via adaptor proteins (Cho et al., 2005; Macdonald, 2005; Niessing et al., 2002, 1999, 2000). Our results with the N51A and bcd^MM^ lines reveal Bcd binding to *caudal* is unlikely to have a major impact on the diffusion of Bcd in the cytoplasm. One future test of this result is to measure Bcd dynamics in embryos over- or underexpressing *caudal*. On the other hand, our results with NLSbcd^HD^ show that the *bcd* homeodomain does impact protein dynamics in the cytoplasm. However, the specific domains behind this behaviour remain unclear. There are additional factors, such as Zelda, that may also play a role in spatially varying the effective Bcd dynamics (Drocco et al., 2011). Finally, we observe that the relative fraction of Bcd in the fast/slow forms spatially varies across the embryo. This suggests that the transition between the slow and fast cytoplasmic forms is non-linear, and in particular may depend non-linearly on the local Bcd concentration. More refined spatial dissection of the dynamics will help to illuminate this behaviour more clearly. Such behaviour may enable Bcd to adapt to embryos of variable size (Huang et al., 2020). One possible other factor may be cytoplasmic flows, which have recently been demonstrated to play a role in refining the Bcd gradient (Hernandez-Lopez et al., 2023).

Bcd operates as a morphogen within the *Drosophila* blastoderm. Are our observations potentially relevant for other morphogens, which are typically extracellular ligands? Molecules can be hindered either passively due to micro-geometries of the diffusing environment (Kuhn et al., 2022) or actively stalled by binding and unbinding of the specific receptors on the cell surfaces or transient binding of interacting proteins (Müller et al., 2013). Recent evidence in Nodal suggests that its movement is akin to hindered diffusion (Kuhn et al., 2022), resulting in an exponential morphogen distribution. During expansion of *Drosophila* wing imaginal discs, the distribution of Dpp activity can be scaled to the size of the tissue via pentagone (Hamaratoglu et al., 2011), a feedback regulator of Dpp, and by Dpp recycling (Romanova-Michaelides et al., 2022). In both the above examples, feedback between the cellular environment (including receptor distribution) and the morphogen dynamics sets up the gradient. Our results indicate that Bcd dynamics can be also considered as hindered diffusion in the *Drosophila* blastoderm; a balance between diffusion and interactions with the local region (at least in part mediated by the Bcd homeodomain) generates the effective dynamics that create the Bcd gradient. It seems likely that, in the cytoplasm, Bcd movement is hindered by the cytoskeletal structures, which could be pertinent for extracellular morphogens. Therefore, we predict that our key observation – that the effective morphogen dynamics are not constant in space – will be relevant for other morphogen systems.

We have used dual colour imaging to ensure that we are recording accurately either nucleus or cytoplasmic pools. An alternative strategy is to use imaging-FCS (Fig. S3) (Krieger et al., 2015). With this approach, the available timescales are reduced (lowest time resolution about 0.1 ms) but spatial cross-correlation can be explored. This approach has the advantage of being able to image throughout mitosis, as spatial movements of nuclei can be accounted for. It will be interesting to explore how Bcd dynamics change during nuclear division in the blastoderm.

Overall, the combination of new Bcd mutant eGFP lines with careful FCS measurements has revealed insights into how a morphogen gradient can form across the required spatial and temporal scales. The apparent concentration-dependent dynamics of Bcd provides a mechanism for how the Bcd gradient can form sufficiently quickly while also having slower more local dynamics. Outstanding questions include: (1) what interactions are determining Bcd dynamics in the cytoplasm; (2) is Bcd diffusivity concentration dependent and, if so, how; and (3) do other morphogens display position and/or concentration-dependent dynamics?

## MATERIALS AND METHODS

### Generation of fly lines

eGFP::Bcd fly line was a gift from Thomas Gregor (Princeton University, NJ, USA). The eGFP::Bcd line was generated by introducing eGFP coding region into the N-terminus of the *bcd*-coding region after the start codon in the pCaSpeR7 Bcd plasmid (Barolo et al., 2000; Hazelrigg et al., 1998; Thummel and Pirrotta, 1992). eGFP tagged Bcd was brought in the background of *bcd^E1^* null allele to ensure that it is the only source of Bcd in these embryos. eGFP::Bcd was crossed with His2A::mCherry (Krzic et al., 2012) to mark the nuclei of early blastoderm embryos, such that the 560-laser line could act as a reference to mark the nucleus and to differentiate the cytoplasmic region in the syncytium. eGFP::Bcd^N51A^, eGFP::Bcd^R54A^ and eGFP::Bcd^MM^ mutant lines were generated by PCR. For N51A and R54A, the Asn (N) at position 51 and Arg (R) at position 54 of Bicoid homeodomain, respectively, are edited to Ala, as generated by Niessing et al. (2000). The PstI-SalI fragment of the Bicoid homeodomain sequence bearing appropriate base pair changes (N51A and R54A) replaces the existing PstI-SalI fragment of the eGFP::Bcd pCaSpeR7 plasmid by restriction digestion. Likewise, multi-mutant eGFP::Bicoid (eGFP::bcd^MM^) is generated through sequential editing in eGFP::Bcd^N51A^ pCaSpeR7 construct background. Fragments containing the mutant YIRPYL motif (AIRPYR) (Niessing et al., 2002) and the mutant PEST (bcd^5aa^) domain of Niessing et al. (1999) are reintroduced and replace the existing fragments of the eGFP::Bcd^N51A^ pCaSpeR7 construct. Therefore, eGFP::Bcd^MM^ bears targeted mutations in the homeodomain (N51A), YIRPYL motif and PEST domain (Figs 4A and Fig. S8A).

For control, we generated an eGFP::NLS line expressed using the Bcd regulatory sequences as described by Gregor et al. (2008). We introduced a PCR amplified fragment containing a single copy of the SV40 nuclear localisation sequence and a stop codon (NLS-STOP) in between the eGFP sequence and Bcd-coding region in the eGFP::Bcd pCasper7 plasmid explained in the preceding paragraph (illustrated in Fig. 5A). Upon translation, only the eGFP::NLS part of the eGFP-NLS-Bcd mRNA is expressed, generating a gradient across the A-P axis of the embryo (Fig. S4A; Fig. 5B) (Gregor et al., 2008). We generated the eGFP::NLSbcd^HD^ line by introducing the homeodomain sequence of Bcd that encodes 60 amino acids. We PCR amplified the 180 bp homeodomain sequence from exon 3 of the Bcd genomic region using primers that had a GS_6_ linker and Kpn1 restriction site at their extreme ends. The Kpn1-GS_6_-bcd^HD^-GS_6_-Kpn1 fragment was digested and inserted into the newly introduced Kpn1 site (between eGFP and NLS-STOP) of the acceptor eGFP::NLS pCaSpeR7plasmid. In all cases, the eGFP sequence used is from eGFP::Bcd pCaSpeR7, which belongs to eGFP(F64L/S65T) (Gregor et al., 2007b; Patterson et al., 1997). All transgenic lines were injected and generated by Bestgene.

### Preparation of embryos for FCS measurements

eGFP::Bcd*;* His2A::mCherry embryos at n.c. 9 were dechorionated and mounted in PBS on a coverslip in such a way that the dorsal surface of the embryo struck to the surface of the coverslip and faced the objective. The dorsal surface is flatter compared with the curved ventral surface and the dorsal surface covers a larger area with shorter *z*-depth. The cortical planar surface of the embryo, which contained the maximum number of in-focus nuclei, was selected for FCS measurements (Fig. 1A). His2A::mCherry marked nuclei and was used as a reference for the nuclear Bcd FCS measurements; the area devoid of His2A::mCherry was used for the cytoplasmic Bcd measurements. Typically, the FCS measurements were performed for 60 s in the cytoplasm and for 20-40 s in the nuclei. The reduction in the duration of nuclear measurements were due to fluctuation of the nuclear positions during imaging.

### FCS measurements

FCS was carried out using a FV1200 confocal microscope (Olympus) equipped with a time-resolved FCS upgrade kit (PicoQuant) at 25°C. The 488 nm pulse wave laser line was used to excite eGFP::Bcd through an UplanSApo 60× NA 1.2 water immersion objective (Olympus). The laser power was optimised using nuclear eGFP::Bcd at n.c. 14. Laser powers of 2-3 μW, which had better signal-to-noise ratio and minimal photobleaching, were used for FCS measurements both in the nucleus and cytoplasm regions of the anterior and posterior domains of the embryo (Fig. S10). The fluorescence emission was passed through a 405/488/543/635 dichroic mirror (Chroma Technology), a confocal pinhole of one airy unit (120 μm) and then split using a 50/50 mirror plate. The split emission was detected simultaneously by an avalanche photodiode (SPCM-AQR14; PerkinElmer). Dual detector measurements effectively remove after-pulsing information in the FCS curves. The photon counts from the detector were registered by a TimeHarp 260 time-correlated single-photon counting board (PicoQuant) and processed by the SymPhoTime (Kapusta, 2010) software (PicoQuant). The same software was also used to calculate the auto-correlation function. For further details on FCS calibration, see supplementary Materials and Methods.

### Qualitative comparison of ACF curves

ACF curves of individual measurements were normalised and compared to show their qualitative differences. The ACF curves were normalised as *G*(*τ*)−*G*(∞)/*G*(0)−*G*(∞). *G* (0) is the amplitude of the ACF curves (typically at lag time 0.0001 s), *G* (∞) is the convergence value at the longer lag times (typically 1 s). The curves and graphs are plotted in GraphPad Prism version 10.0.0.

### FCS curve fitting

The FCS curves of each measurement were fitted by three-dimensional diffusion models involving diffusion of 1 and 2 species with and without reversible switching of the fluorophores to dark states, using Igor-Pro (8.03), FCS data processing plug-in, Version 2.1, https://www.dbs.nus.edu.sg/lab/BFL/confocal_FCS.html. See supplementary Materials and Methods for further details.

### Estimation of eGFP::Bcd concentration in the measurement volume

We estimated the concentration of eGFP::Bcd and eGFP::NLS through generating a standard curve (Fig. S11). A high known concentration of Atto-488 dye was serially diluted to lower concentrations (10, 7, 5 and 2 nM) and the correlation amplitudes determined. Similarly, known concentrations (3, 4, 6 and 15 nM) of eGFP *in vitro* solutions were also used to generate a linear line, as in Atto488. A plot of the inverse of correlation amplitude, i.e. the number of molecules versus concentration in nM fit a linear line. The unknown concentrations of eGFP::Bcd and eGFP::NLS in the embryos were found from the line equation shown in Fig. S11B,D. For details on the issues on the concentration estimation of posterior domain, see supplementary Materials and Methods.

### SPIM based imaging-FCS

Embryos were imaged on a home build selective plane illumination microscope (SPIM) set up as described previously (Dhasmana et al., 2021; Krieger et al., 2015; Ng et al., 2016). For elaborated details on the configuration, mounting of embryos for SPIM-FCS and processing, see supplementary Materials and Methods section.

### Quantification of eGFP::Bcd, eGFP::Bcd^N51A^, eGFP::Bcd^R54A^, eGFP::Bcd^MM^and eGFP::NLS gradients

Time lapse videos of embryos (eGFP::Bcd and Bicoid mutants with H2b::mCherry background) at n.c. 14 were acquired along the longitudinal plane passing through midline of the embryo. For each embryo, two separate images of 512×512 pixels along the mid sagittal plane covering anterior and posterior domains of the embryo were stitched together. The images were captured at 8 bits/pixel, pixel dwell time of 3 µs, line averaging 4, five *z*-sections of 1 µm each. The conditions captured maximum in-focus peripheral nuclei along the mid-sagittal plane in the Zeiss LSM 710 confocal scanning microscope. The nuclear eGFP intensities along the embryo circumference at n.c. 14 were measured manually by placing an elliptical window in the nuclear centres (Histone::mCherry marked nuclei) that covers maximum nuclear area of all nuclei around the peripheral edge of the embryo in ImageJ.

The measured raw nuclear intensity profile along the circumference of the embryo was background corrected and plotted against the actual length of the embryo. We estimated background using two approaches. First, we simply subtracted the background outside the embryo. However, this is does not account for the illumination variation across the embryo and also possible yolk effects. We also imaged embryos expressing H2b::mCherry but not Bcd::eGFP. For these embryos, we imaged in both 488 nm and 561 nm channels. The 488 nm channel provides an estimate of the background signal for our Bcd::eGFP measurements (similar to Gregor et al., 2007a).

### Modelling of gradient formation

We considered Bcd to be produced within a region close to the anterior, defined by *f*(*x*, *x*_*s*_)=1 if *x*<*x*_*s*_ and *0* otherwise. We take *x*_*s*_=30 μm, consistent with previous observations of *bcd* mRNA and fits from our Bcd::eGFP measurements. We also allow this to be a fitting parameter in the equations. *D* is taken from the FCS measurements. We took *μ*=1/50 min, consistent with eGFP lifetime in the early *Drosophila* embryo (Durrieu et al., 2018). Again, we also allow this to be a fitting parameter in the simulations, but it stays around this bound. Boundary conditions ∂_*x*_*φ*(*x*=0, *t*)=0 and ∂_*x*_*φ*(*x*= *L*, *t*)=0 were used, where *L*=500 μm represents the embryo length. We also account for the time taken for Bcd to fold (around 45 min). Formulae and parameter details are provided in the supplementary Materials and Methods.

For eGFP::Bcd, the equations are given in the main text. All equations solved in 1D using MATLAB pdepe solver with zero flux boundary conditions at *x=L*. Parameter fitting was carried out as follows. We performed 100 simulations for each model. Each simulation was fitted to a randomly generated concentration profile formed by using the measured mean and s.d. in the concentration at each position; i.e. we fit to a range of profiles that are defined by the experimental error (a form of bootstrapping). We then calculate the mean and s.d. of each fitting parameter. Parameter minimisation was performed using *fminsearch* in Matlab. Code has been deposited in Github (https://github.com/TimSaundersLab). Further details are provided in the supplementary Materials and Methods. Full lists of parameter values and different models considered are provided in the supplementary Materials and Methods.

## Supplementary Material



10.1242/develop.202128_sup1 Supplementary information
