# Supplementary material for: Long-range formation of the Bicoid gradient requires multiple dynamic modes that spatially vary across the embryo
Source: Development. 2024 Feb 12;151(3):dev202128. doi: 10.1242/dev.202128 (PMC10911119; doi:10.1242/dev.202128)
Supplement: Supplementary information [file develop-151-202128-s1.pdf]

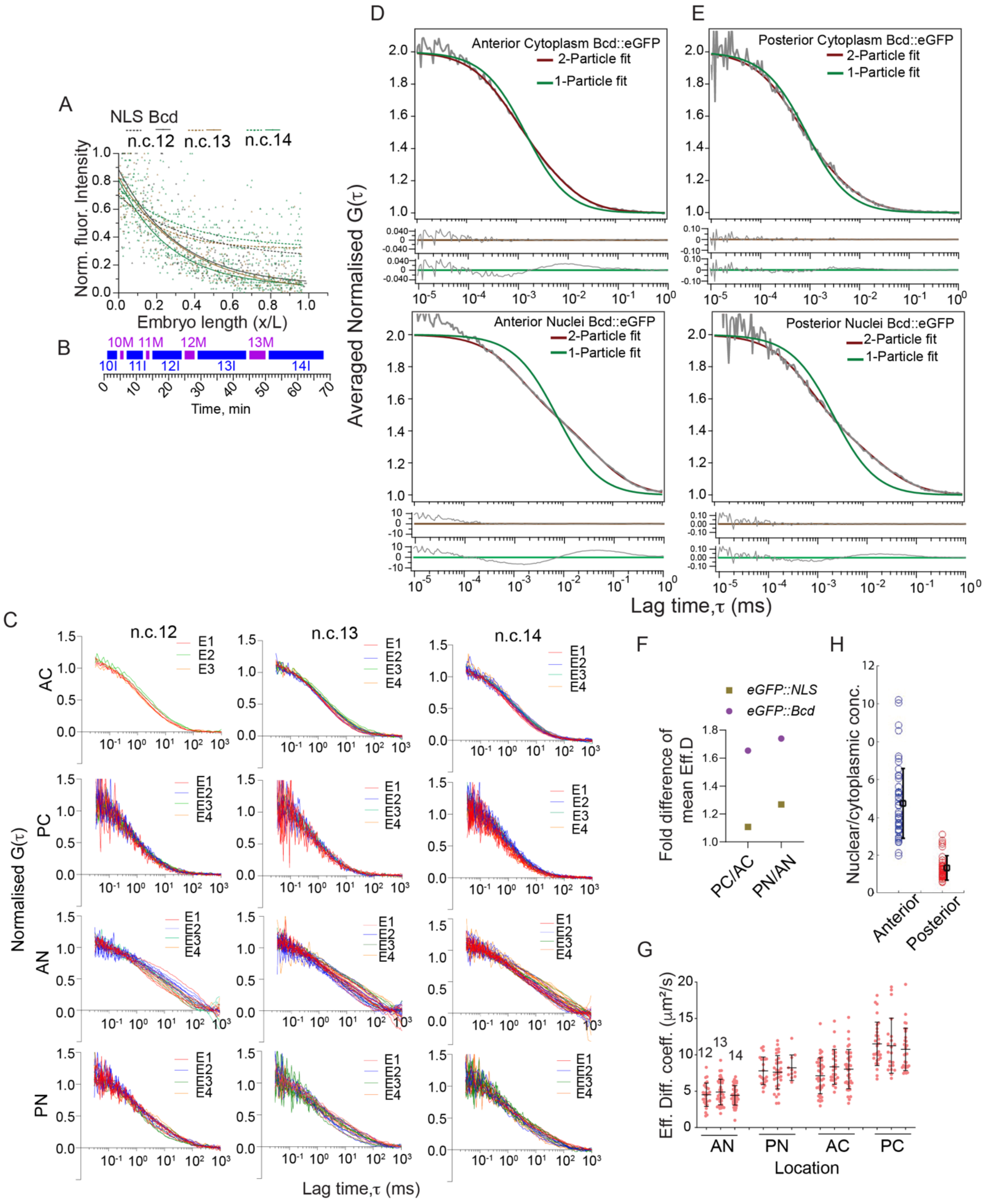

Fig. S1. FCS and fitting of eGFP::Bcd (related to Figure 1)

(A) Comparison of the gradient profiles of eGFP::Bcd and eGFP::NLS plotted with normalised fluorescence intensities in Y-axis and the normalised embryo length (x/L). Dots show individual nuclear intensities, and the solid lines are fits to exponential profiles. (B) Time profile of the early embryo from n.c. 10 to 14 at 25ºC. I indicates interphase, and M, mitosis. (C) Normalised ACF curves of eGFP::Bcd in the cytoplasmic and nuclear compartments of anterior (Anterior Cytoplasm, AC, Anterior Nuclei, AN) and posterior (Posterior Cytoplasm, PC, Posterior Nuclei, PN) in nuclear cycle (n.c.) 12, 13, and 14 interphases. (D, E) Comparison of ACF curves (grey) with residues fitted using 3D 1-particle and 2-particle diffusion model in the anterior cytoplasm (D), nuclei (D') and posterior cytoplasm (E), nucleus (E'). (F) Effective diffusion coefficients of nuclear and cytoplasmic locations of the anterior and posterior domains are compared for individual n.c. 12, 13, and 14. (G) Fold change in the mean diffusion coefficient across the embryo for the cytoplasmic and nuclear compartments. Comparison for eGFP::Bcd (circles) and eGFP::NLS (squares) are shown. (H) Ratio of measured apparent concentration (from ACF curve amplitude) in the nuclei and cytoplasm of anterior and posterior compartments. Note the posterior concentration is approximated as the signal is very low in the posterior compartment.

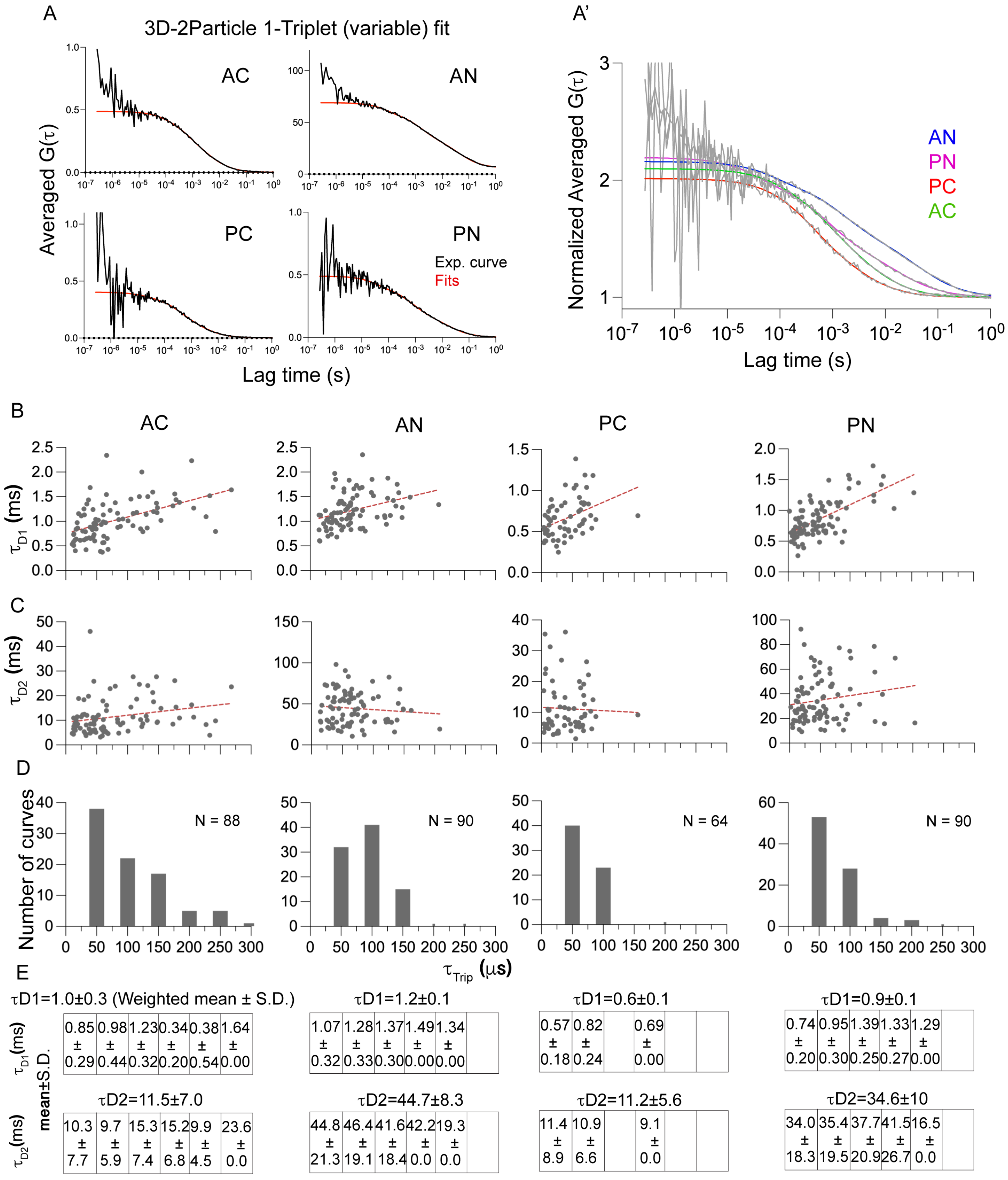

Fig. S2. Fitting and analysis of eGFP::Bcd with 3D 2-particle and variable triplet (related to Fig. 1)

(A) ACF curves of eGFP:Bcd (black) in the cytoplasm and nuclear compartments of anterior and posterior domains from nuclear cycles 12-14 fitted with 3D 2-Particle 1-triplet model with triplet states are allowed to vary. The normalised curves are shown in A'. The ACF curves are fitted from 10^{-5}s to 1s as the curves below 10^{-5} are noisy. (B,C) Scatter plot showing τ_{D1} (B) and τ_{D2} (C) distribution with respect to the $\tau_{Triplet}$ values for each curves. ACF curves from n.c.12, 13 and 14 are considered together in this analysis as there are no variations in D values observed across these nuclear cycles. (D) Distribution of ACF curves every 50μs of $\tau_{Triplet}$ values ranged from 0 to 300μs. N represents total number of curves. (E) The mean and S.D. of τ_{D1} and τ_{D2} values of binned ACF curves are in D. Note: Maximum number of curves have $\tau_{Triplet}$ value of <100μs in all four cases of ACF curves. Weighted means and S.D. of τ_{D1} and τ_{D2} are also shown in E that matches with the τ_{D1} and τ_{D2} values of 2-Particle fits without considering triplet in Table 2 and 3.

Development • Supplementary information

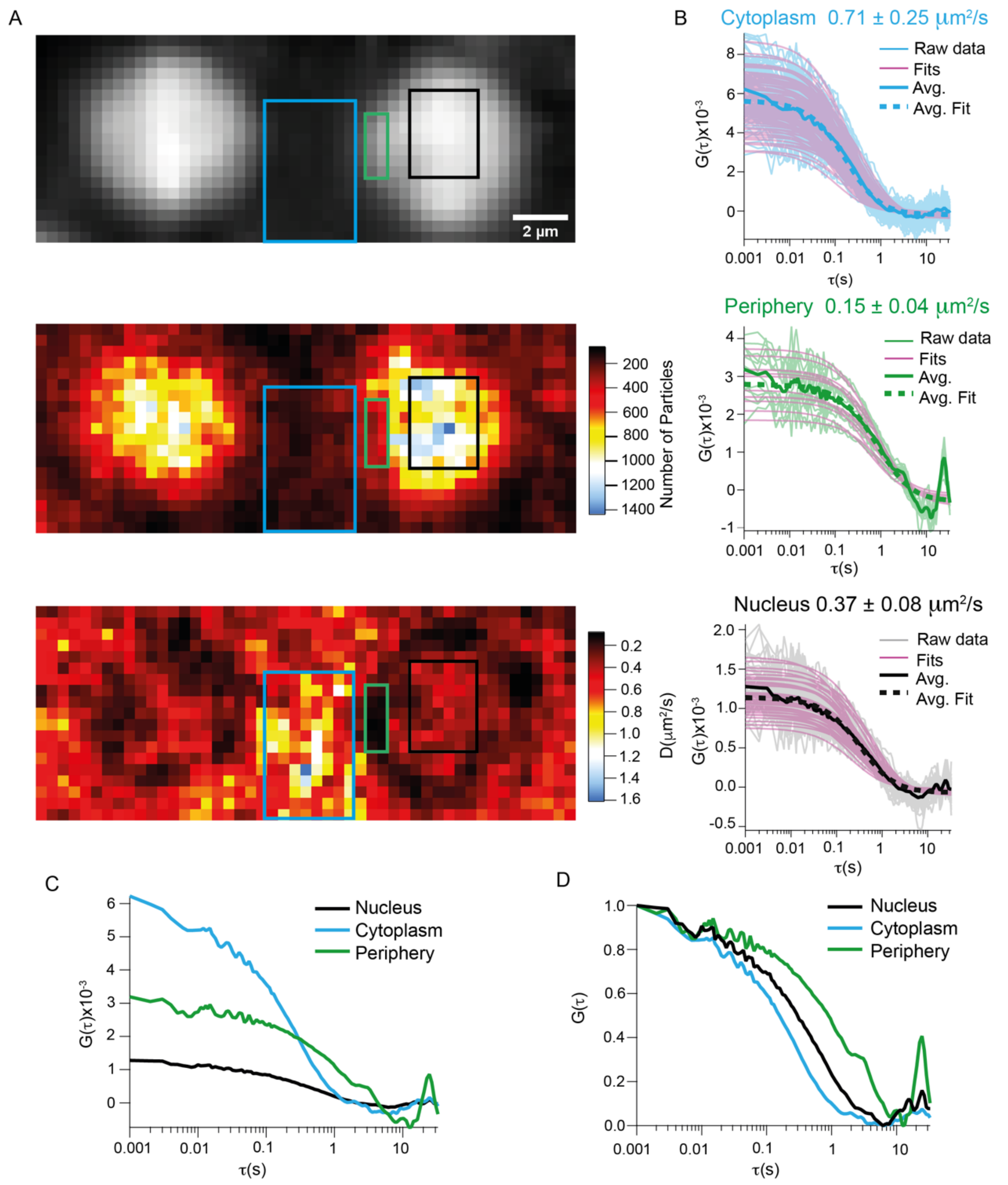

Fig. S3. SPIM-based Imaging-FCS on eGFP::Bicoid

(A) Intensity sum projection (top), number of particles (N, middle), and diffusion coefficient (D, bottom) maps for eGFP::Bcd in the anterior margin of the embryo during n.c. 13. The spatial maps show Bcd's relative localisation and dynamics in and around the nuclei. As seen from the N map, Bcd mostly localises inside the nucleus, followed by the nuclear periphery, and its concentration in the cytoplasm is relatively low. Conversely, the D map shows that Bcd diffuses faster in the cytoplasm than in the nucleus and is relatively slow at the nuclear periphery. (B) Individual ACFs from pixels, fits and the average ACFs for different ROIs representing the cytoplasm (cyan n = 104), nuclear periphery (green n = 12) and inside the nucleus (black n = 48). Based on the fit values obtained, the diffusion coefficients in different spaces vary as $D_{cytoplasm} > D_{nucleus} > D_{periphery}$. (C) Comparison between the average ACFs from the nucleus, cytoplasm, and nuclear periphery ROIs. The ACF amplitude is highest for the cytoplasm, followed by the nuclear periphery and is the

lowest for the ACF from the nucleus. As the amplitudes of the ACFs are inversely proportional to the number of particles, the ACFs show that $N_{nucleus} > N_{periphery} > N_{cytoplasm}$. (D) Comparison between the normalised average ACFs from the nucleus, cytoplasm, and nuclear periphery ROIs.

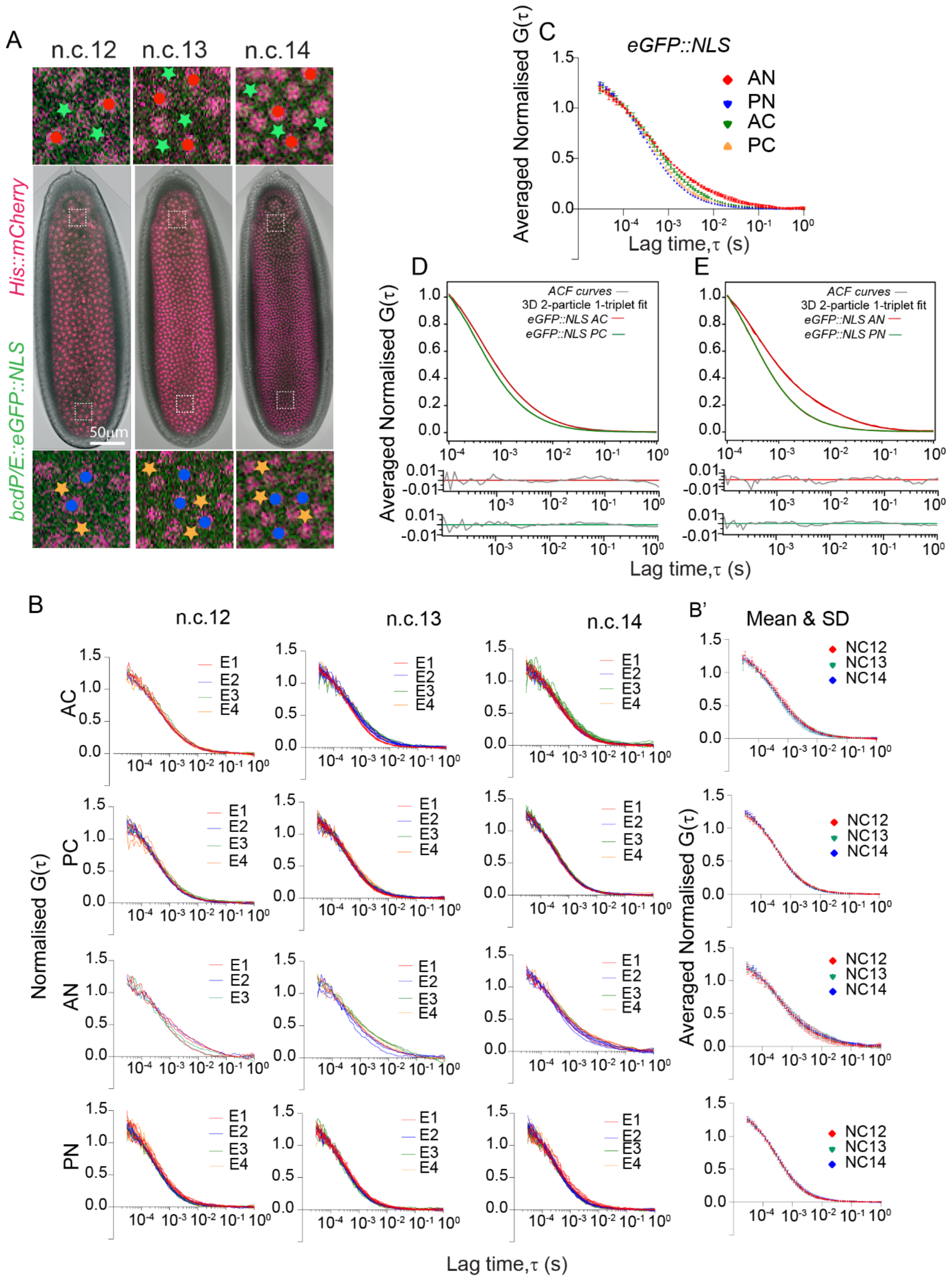

Fig. S4. FCS and fitting of eGFP::NLS (related to Figure 2)

(A) *Drosophila* blastoderm showing the interphase periods of n.c. 12, 13 and 14. Nuclei (mCherry::His2Aν, red) and eGFP::NLS (green). Dots and stars indicate cytoplasmic and nuclear regions, respectively, where FCS measurements are carried out in the anterior (red) and posterior (green). (B-B') Normalised ACF curves with mean and S.D. of eGFP::NLS in cytoplasmic and nuclear compartments of the n.c. 12,13, and 14 interphases. (C) Comparison of normalised, averaged ACF curves with mean and S.D. of eGFP::NLS in cytoplasmic and nuclear compartments in n.c. 12,13, and 14 interphases. (D-E) ACF curves (grey) with residues fitted with 3D 2-particle 1-triplet diffusion model for cytoplasm (D) and nuclei (E).

Development • Supplementary information

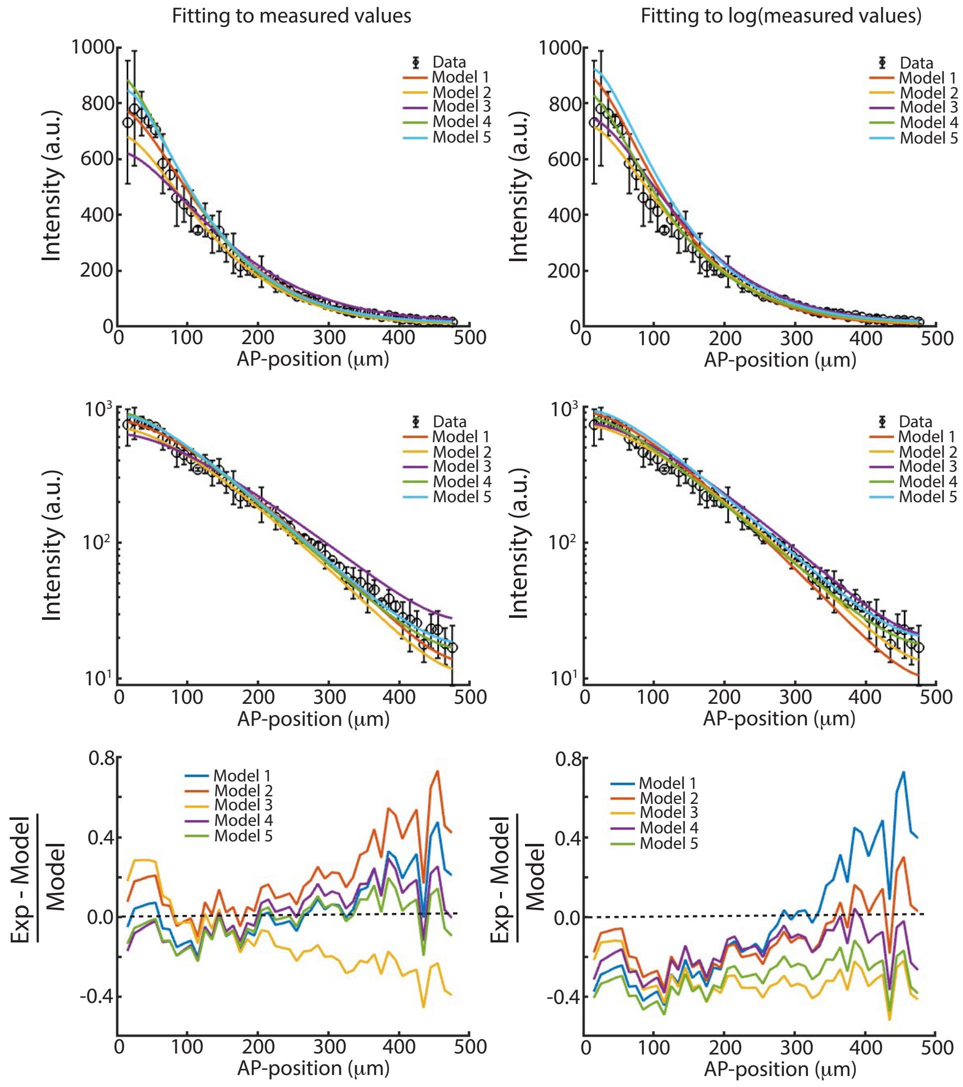

Fig. S5. Model fitting (related to Figure 3)

Model fits as described in the Supplementary Information to the experimental Bcd::eGFP profile. Left column represents fitting to background-subtracted intensity values. Right column represents fitting to log(background-subtracted intensity values). Experimental data shown as mean and s.d.. Top row: fit to data on linear scale. Middle row: fit to data on logarithmic intensity scale. Bottom row: Quality of fit across the embryo axis. Perfect fit shown by dashed black line.

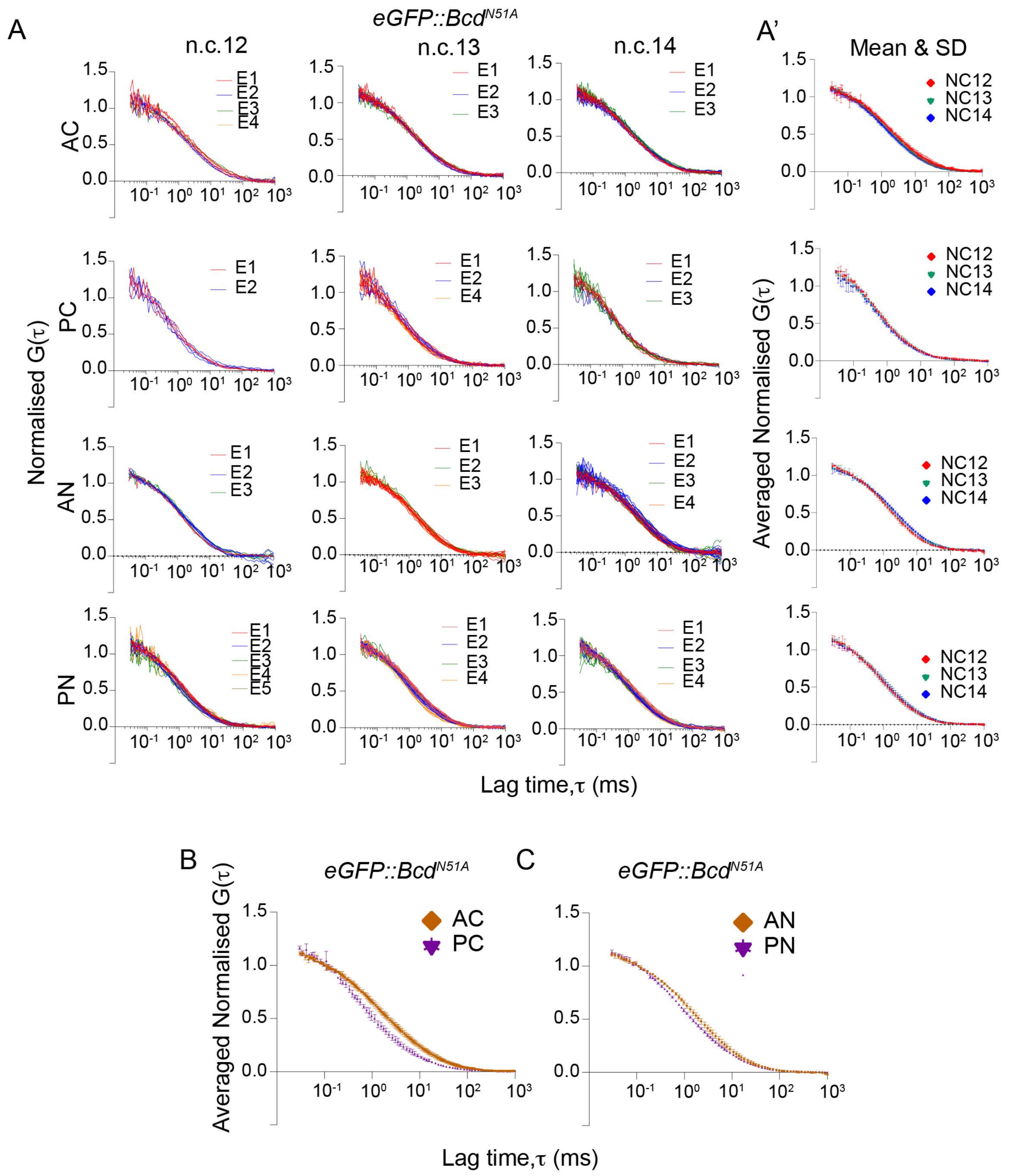

Fig. S6. FCS and fitting of eGFP:Bcd^{N51A} (related to Figure 4)

(A-A') Normalised ACF curves with mean and S.D. of eGFP::Bcd^{N51A} in the cytoplasmic and nuclear compartments of the n.c. 12,13, and 14 interphases. (B-C) Normalised average ACF curves of eGFP::bcd^{N51A} in the cytoplasmic (B) and nuclear (C) locations of the anterior and posterior domains of the embryo.

Development • Supplementary information

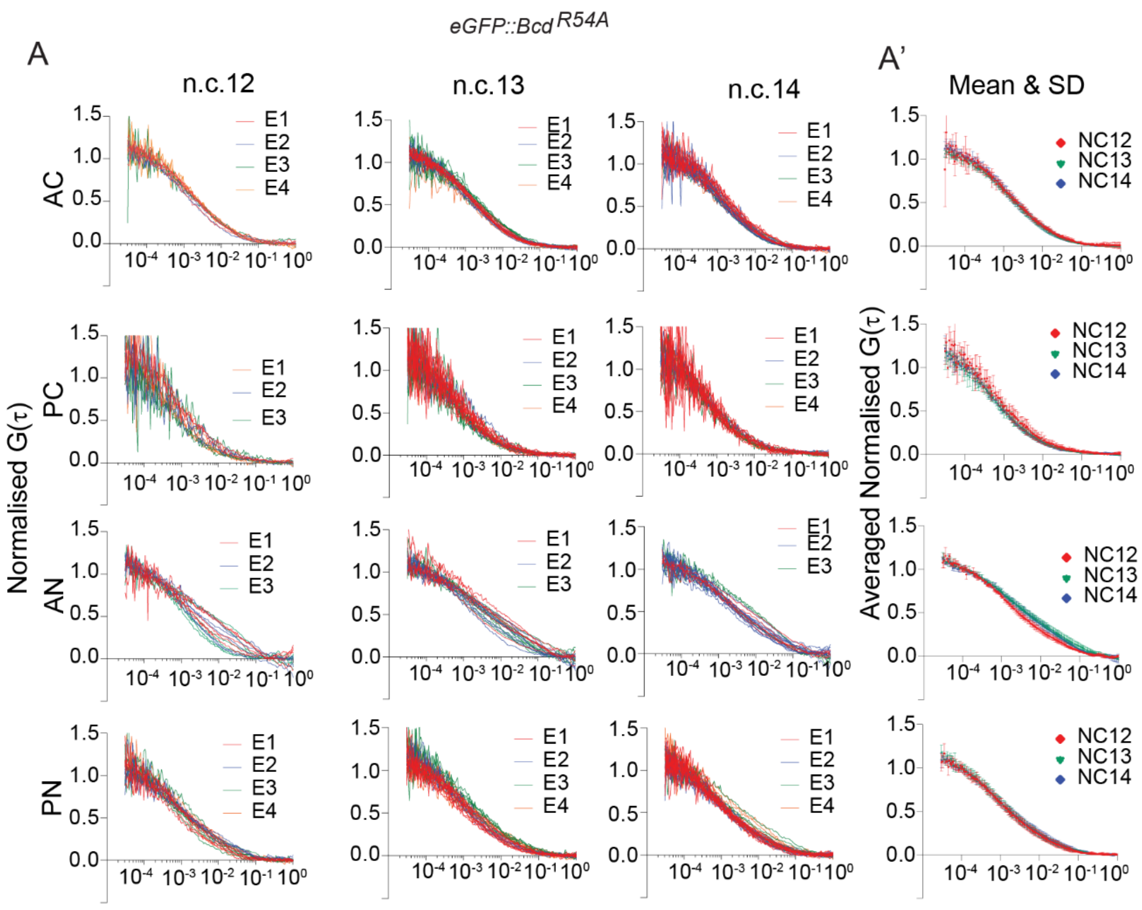

Fig. S7. Dynamics of eGFP::Bcd^{R54A} (related to Figure 4)

(A) Normalised ACF curves with mean and S.D. of eGFP::Bcd^{R54A} in the cytoplasmic and nuclear compartments of the n.c. 12,13, and 14 interphases. Normalised ACF curves from multiple embryos are shown. Lag times from 10^{-4} sec to 1sec are shown for visual clarity. (A') Normalised and averaged autocorrelation ACF curves with mean and S.D. of eGFP::Bcd^{R54A} in the cytoplasmic and nuclear compartments of the n.c. 12,13, and 14 interphases.

Development • Supplementary information

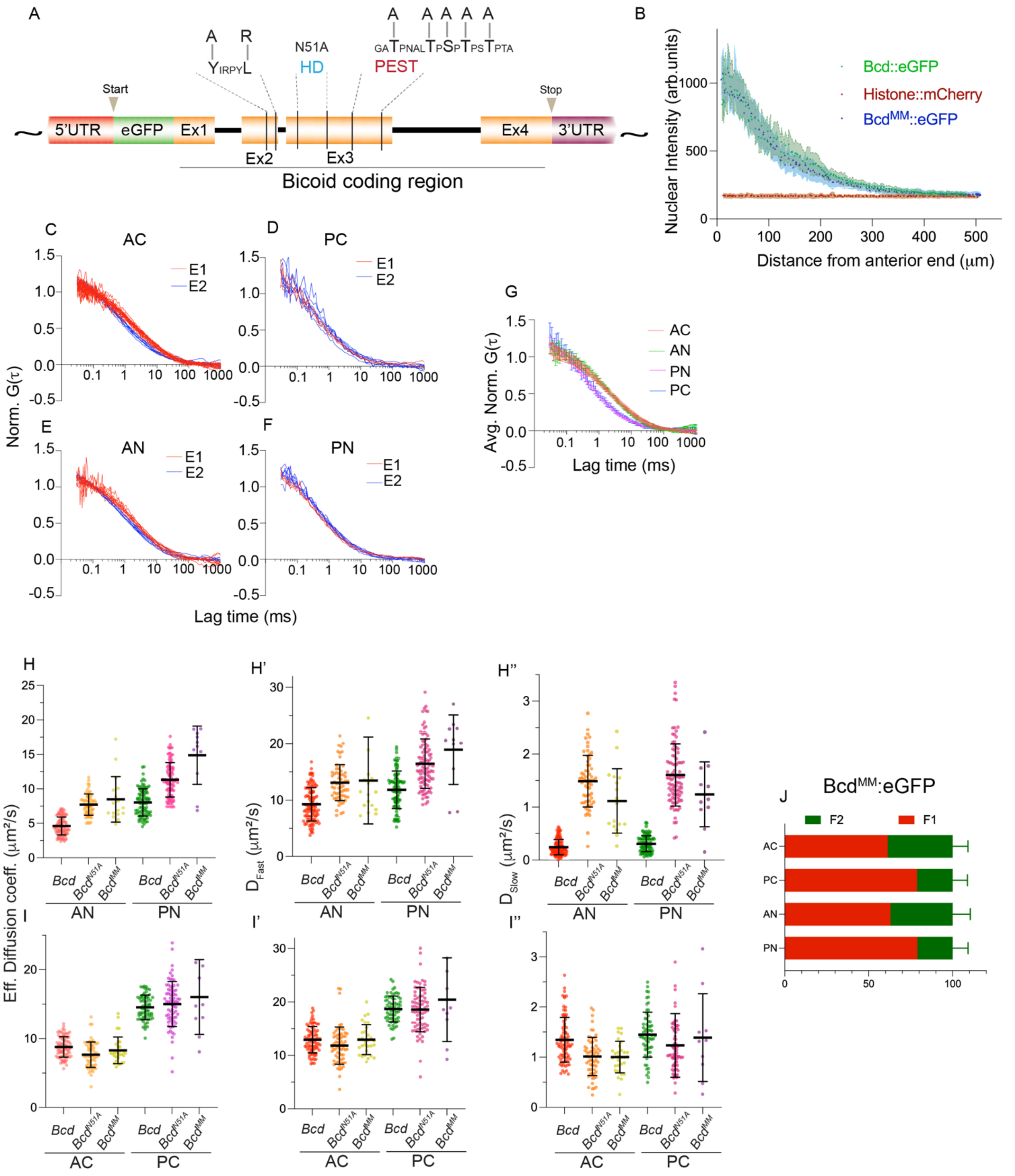

Fig. S8. FCS of eGFP:: Bcd^{MM} (related to Figure 4)

(A) Schematic of the point mutations introduced in homeodomain, YIRPYL motif and PEST domain that presumably abolishes the function of these domains in eGFP::Bcd^{MM}. (B) Gradient profile of eGFP::bcd^{MM} compared to eGFP::bcd and Histone:Mcherry. (C-G) Normalised individual ACF curves from anterior and posterior compartments of eGFP:Bcd^{MM} embryos are compared. Lag times from 10^{-4} sec to 1sec are shown for visual clarity. (H-I) Scatter plots of the effective diffusion (H,I), D_{fast} (H',I') and D_{slow} (H''-I'') values compared among eGFP:Bcd, eGFP:Bcd^{N51A} and eGFP:Bcd^{MM} . (G) Bar plots comparing the fractions of slow and fast components of the eGFP:bcd^{MM}.

Development • Supplementary information

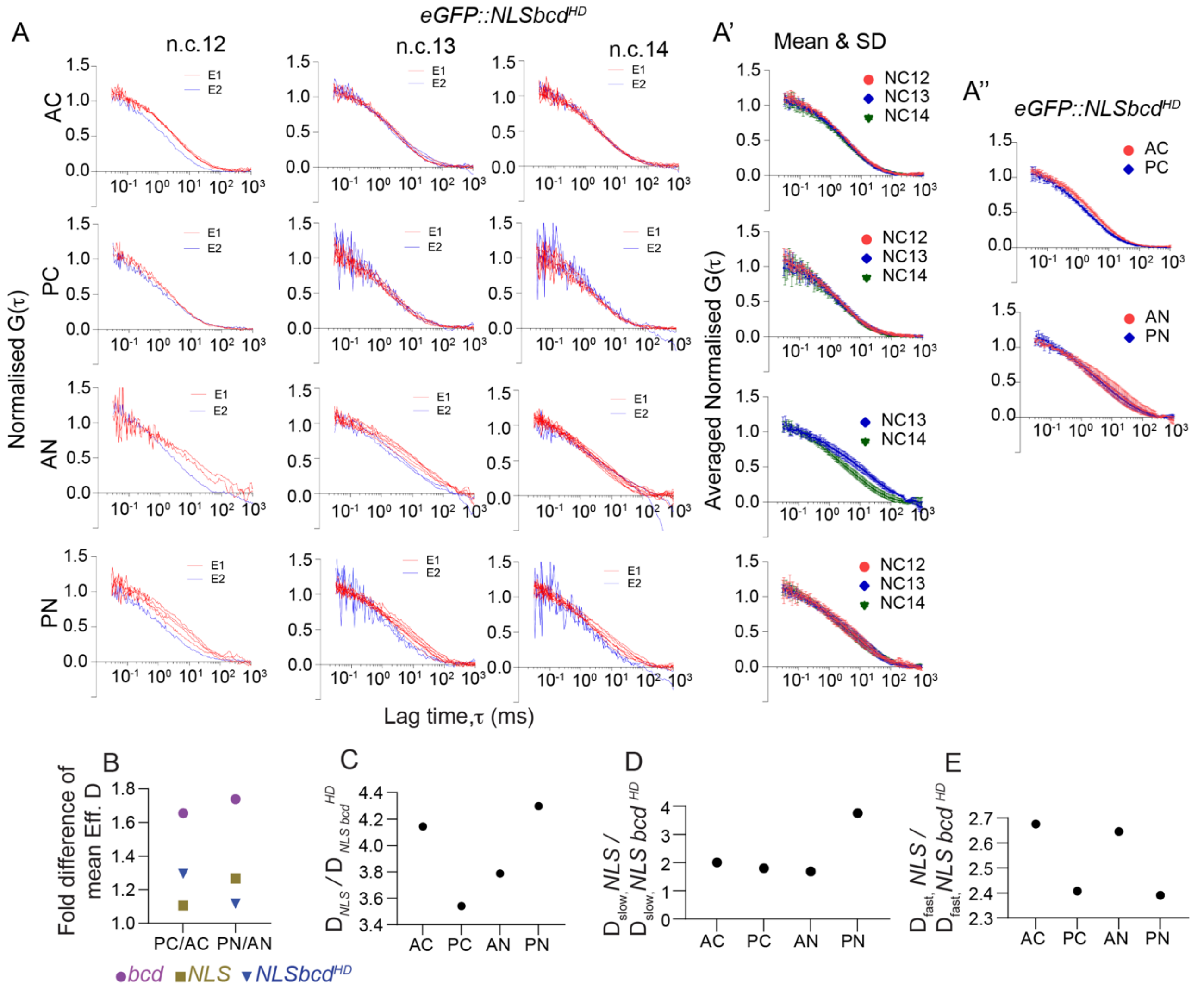

Fig. S9. FCS of eGFP::NLSbcd^{HD} (related to Figure 5)

(A-A') Normalised ACF curves with mean and S.D. of eGFP::NLSbcd^{HD} embryos in the cytoplasmic and nuclear compartments of the n.c. 12,13, and 14 interphases. Normalised ACF curves from two embryos (right) are compared. Lag times from 10^{-4} sec to 1sec are shown for visual clarity. (A'') Qualitative comparison of normalised ACF curves with mean and S.D. of eGFP::NLSbcd^{HD} in the cytoplasmic and nuclear compartments of the n.c. 12,13, and 14 interphases. (B-E) Change in relative diffusivity of eGFP::NLSbcd^{HD} compared with eGFP::NLS at different locations within the embryo.

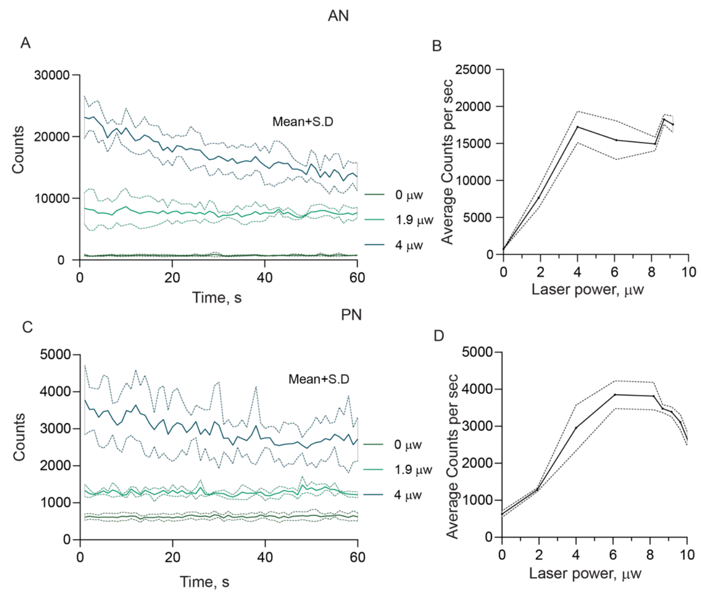

Fig. S10. Laser power optimisation

(A, C) line plot showing the time trace of the eGFP:Bcd in the anterior (A) and posterior nuclei (C). Mean (solid line) and SD (dotted line) of the time trace are shown for laser powers 0, 1.9 and 4μw. (laser power was measured before the objective) (B, D) Mean counts per sec increases with laser power and gets saturated above laser power of 4μw due to photobleaching.

Development • Supplementary information

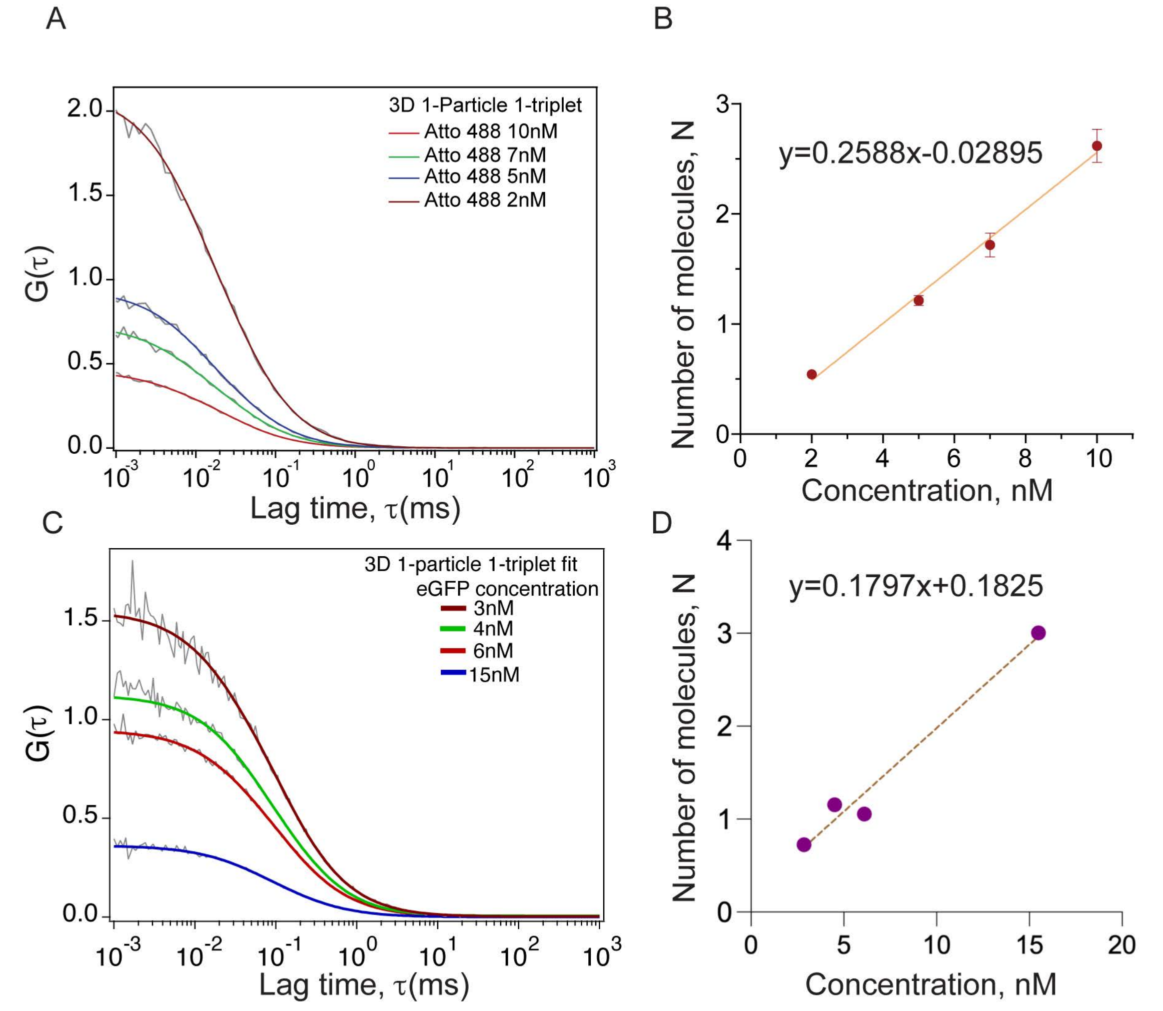

Fig. S11 (related to Figs. 1,2 and S1). Calibration of effective volume to estimate the concentration (A,C) Normalised ACF curves (grey) of Atto-488 (A) and of eGFP (C) of different concentration and the 1-particle 1-triplet fits. (B,D) linear increase in number of molecules in the confocal volume upon increasing the concentrations of Atto-488 and eGFP invitro. The standard curve shows negligible changes in both cases. Equation from Atto488 is used for concentration measurements.

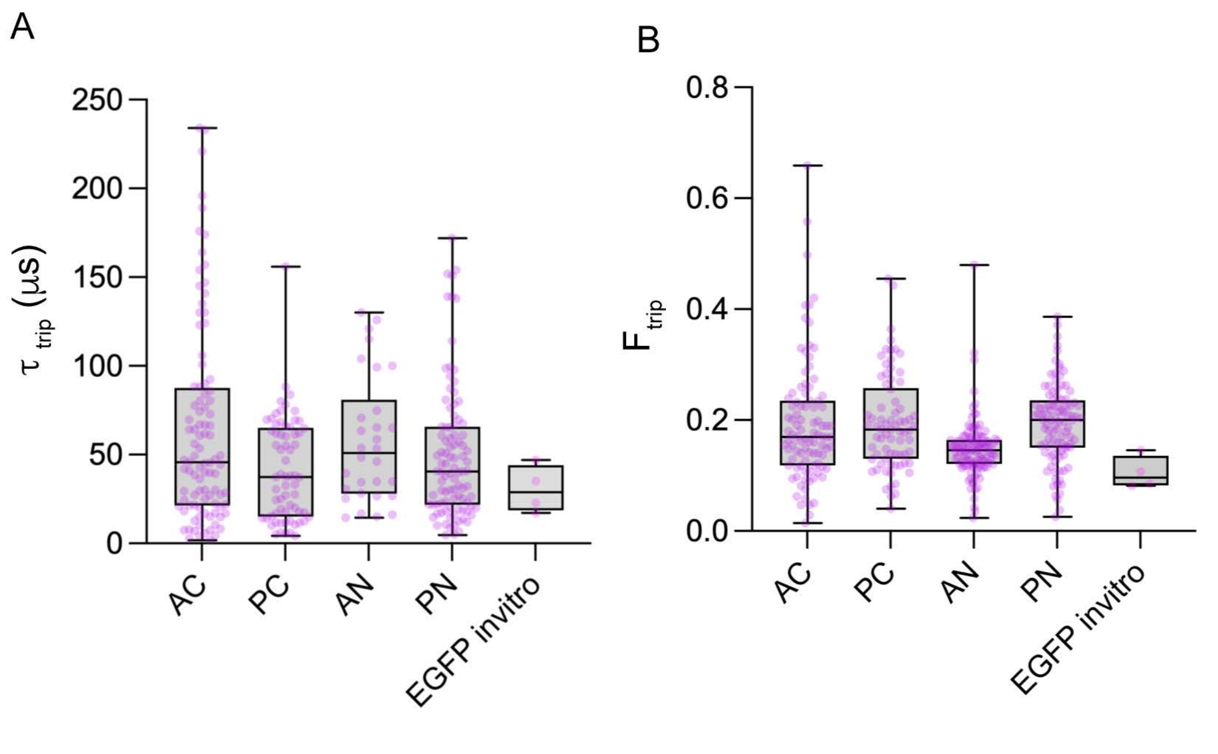

Fig. S12 (related to S2). Estimation of triplet lifetime, $t_{triplet}$ and its fraction ($F_{triplet}$)
(A) Box-whisker plot showing the values of the characteristic times of photophysical processes, $t_{triplet}$, from multiple ACF curves measured from 4 embryos, across all compartments. Triplet information from eGFP in PBS is also included. The fraction (F_{trip})is given in B. AC- anterior cytoplasm, PC-posterior cytoplasm, AN-anterior nuclei, and PN-posterior nuclei.

Development • Supplementary information

Table S1. Comparison of parameter values of 1-particle and 2-particle fits of eGFP::Bcd with fixed G(0) and G(μ) (related to Fig. 1 and Fig. S1).

	1-Particle			2-Particle							
Nuclear cycle	TauD(ms)	D(μm2/s)	Reduced Chi^2	TauD1(ms)	D1(μm2/s)	TauD2(ms)	D2(μm2/s)	F1 (%)	F2(%)	Eff.D (μm2/s)	Chi^2
**Anterior Cytoplasm**											
n.c.12	1.8±0.2	5.7±0.7	5.1	0.9±0.2	11.8±2.6	9.3±2.7	1.1±0.3	69±11	31±11	8.3±1.1	1.1
n.c.13	2.0±0.6	5.4±1.4	4.1	1.0±0.3	10.8±2.9	10.8±4.6	1.0±0.3	69±1	31±1	7.8±2.1	0.8
n.c.14	1.7±0.4	6.1±1.3	3.7	0.7±0.2	14.6±4.1	7.3±2.5	1.5±0.5	62±6	38±6	9.5±2.2	1.0
**Posterior Cytoplasm**											
n.c.12	1.1±0.2	9.3±1.4	1.0	0.6±0.2	16.5±4.7	7.3±3.5	1.6±0.5	76±8	24±8	12.7±2.8	0.4
n.c.13	1.0±0.2	9.7±1.6	1.7	0.6±0.1	16.2±3.5	8.0±3.4	1.4±0.5	78±7	22±7	12.8±2.3	0.6
n.c.14	1.0±0.1	10.5±0.8	3.6	0.6±0.0	17.6±1.4	8.6±2.9	1.3±0.3	78±6	22±6	14.0±0.6	0.7
**Anterior Nuclei**											
n.c.12	7.9±3.7	1.4±0.5	20.9	1.2±0.3	8.7±1.9	44.1±17.0	0.3±0.1	48±5	52±5	4.3±1.1	1.5
n.c.13	8.8±2.6	1.2±0.3	21.9	1.4±0.1	7.0±0.7	57.9±15.4	0.2±0.1	52±3	48±3	3.7±0.5	1.4
n.c.14	11.8±5.8	1.0±0.3	30.2	1.6±0.4	6.6±1.4	76.5±26.0	0.1±0.0	50±4	50±4	3.4±0.9	2.5
**Posterior Nuclei**											
n.c.12	3.1±1.6	3.8±1.4	25.3	1.0±0.5	11.4±3.9	47.9±13.0	0.4±0.1	65±2	35±2	7.6±2.7	1.3
n.c.13	3.0±0.2	3.6±1.0	37.7	0.8±0.2	12.8±2.3	36.6±13.2	0.3±0.1	63±3	37±3	8.3±1.7	2.0
n.c.14	3.9±1.0	2.7±0.6	34.6	1.0±0.2	10.0±2.2	47.9±11.7	0.2±0.1	63±1	37±1	6.3±1.4	1.3

Table S2. Comparison of parameter values of cytoplasmic eGFP:Bcd and eGFP:NLS diffusion fit using 3D 2-particle diffusion model. *(The duration of each cytoplasmic measurement are 60 secs in all cases)*

N.C.	Embryo No. (no. of ACF curves)	$TauD_1$ (ms)	D1 (μm²/s)	Fraction (F_1%)	$TauD_2$ (ms)	D_2 (μm²/s)	Fraction (F_2%)	Eff.Diff. (μm²/s)
**eGFP::Bcd AC : 3D-Diffusion 2-particle model**								
12	1(4),2(4),3(6),4(4)	0.8±0.1	13.2±1.1	66±4	8.5±1.7	1.3±0.2	34±4	9.1±0.8
13	1(9),2(8),3(4),4(5)	0.8±0.1	12.8±0.8	66±5	10.0±1.4	1.2±0.2	34±3	8.7±1.1
14	1(15),2(12),3(12),4(7)	0.8±0.1	12.9±1.1	64±5	7.9±2.1	1.4±0.3	36±5	8.7±1.1
**eGFP::Bcd PC : 3D-Diffusion 2-particle model**								
12	1(3),2(3),3(3),4(4)	0.5±0.0	19.2±1.6	78±3	8.5±1.7	1.3±0.2	22±3	15.3±1.6
13	1(4),2(6),3(7),4(3)	0.6±0.0	18.4±0.7	77±5	7.7±1.3	1.3±0.2	23±5	14.4±0.9
14	1(7),2(7),3(4),4(4)	0.6±0.0	19.0±1.2	79±3	9.8±1.6	1.2±0.1	21±3	15.2±1.2
**eGFP::NLS AC : 3D-Diffusion 2-particle model**								
12	1(4),2(2),3(3),4(2)	0.4±0.0	25.5±1.7	85±2	6.8±1.5	1.7±0.2	15±2	21.8±0.9
13	1(4),2(4),3(7),2(4)	0.4±0.0	27.9±3.6	84±6	6.4±2.6	2.0±0.6	16±6	22.8±1.0
14	1(8),2(7),3(5),4(6)	0.4±0.0	26.4±1.2	84±7	5.6±2.1	2.3±0.5	16±7	22.5±0.8
**eGFP::NLS PC : 3D-Diffusion 2-particle model**								
12	1(3),2(4),3(3),4(1)	0.3±0.0	28.1±3.2	91±0	6.4±1.7	2.1±1.3	9±0	26.1±2.6
13	1(3),2(4),3(6),4(5)	0.4±0.0	27.0±1.3	92±2	5.3±1.8	2.5±0.4	8±2	25.0±1.4
14	1(1),2(4),3(4),4(6)	0.4±0.0	26.4±2.6	94±1	6.9±2.8	2.3±0.6	6±1	25.1±2.2

Table S3. Comparison of parameter values of nuclear eGFP:Bcd and eGFP:NLS diffusion fit using 3D 2-particle diffusion model *(The duration of each nuclear measurement is 20-40 secs.)*

N.C.	Embryo No. (no. of ACF curves)	TauD1 (ms)	D1 (μm²/s)	Fraction (F_1%)	TauD2 (ms)	D2 (μm²/s)	Fraction (F_2%)	Eff.Diff. (μm²/s)
**eGFP::Bcd AN : 3D-Diffusion 2-particle model**								
12	1(6),2(8),3(5),4(3)	1.1±0.1	9.7±1.3	50±4	43.0±6.2	0.3±0.1	50±4	4.9±0.5
13	1(9),2(11),3(6),4(8)	1.2±0.1	9.3±0.5	49±3	66.4±15.6	0.3±0.0	51±3	4.6±0.5
14	1(13),2(10),3(11),4(10)	1.2±0.1	9.0±0.8	49±4	65.2±7.6	0.2±0.0	51±4	4.5±0.5
**eGFP::Bcd PN : 3D-Diffusion 2-particle model**								
12	1(8), 2(4), 3(6), 4(5)	0.9±0.1	11.9±1.5	64±3	36.0±9.4	0.4±0.1	36±3	7.7±1.1
13	1(9), 2(9), 3(10), 4(5)	0.8±0.1	13.1±1.3	61±1	38.3±5	0.4±0.1	39±1	8.1±0.9
14	1(5), 2(8), 3(9), 4(7)	0.9±0.1	11.9±1.3	63±2	42.2±11	0.3±0.1	37±2	7.5±0.7
**eGFP:NLS AN : 3D-Diffusion 2-particle model**								
12	1(2),2(1),3(2)	0.4±0.0	26.1±0.5	84±0	6.6±3.3	1.7±0.8	16±0	21.8±0.5
13	1(1),2(4),3(2),4(3)	0.4±0.0	26.1±2.6	79±6	12.2±2.3	0.9±0.3	21±6	20.4±1.2
14	1(3),2(6),3(1),4(4)	0.4±0.0	27.1±2.0	80±5	15.7±5.4	0.9±0.2	20±5	21.7±2.6
**eGFP::NLS PN : 3D-Diffusion 2-particle model**								
12	1(5),2(3),3(7),4(2)	0.3±0.0	28.2±2.2	95±1	9.6±5.0	2.1±1.4	5±1	26.9±2.2
13	1(4),2(5),3(3),4(8)	0.3±0.0	30.3±2.8	95±1	15.5±9.9	1.2±0.3	5±1	28.9±3.0
14	1(4),2(8),3(7),4(6)	0.3±0.0	28.3±2.8	95±3	14.8±8.3	1.3±0.5	5±3	26.9±2.9

Table S4. Comparison of parameter values of eGFP::Bcd^{N51A} and eGFP::Bcd^{R54A} diffusion fit using 3D 2-particle diffusion model.

N.C.	Embryo No. (no. of ACF curves)	TauD$_1$ (ms)	D1 ($\mu m^2/s$)	Fraction (F_1%)	TauD$_2$ (ms)	D$_2$ ($\mu m^2/s$)	Fraction (F_2%)	Eff.Diff. ($\mu m^2/s$)
**eGFP::Bcd^{N51A} AN: 3D-Diffusion 2-particle model (30s each measurement)**								
12	1(3),2(6),3(4)	0.7±0.1	16.1±3.8	53±3	5.3±0.9	2.0±0.4	47±3	9.3±1.5
13	1(6),2(6),4(3)	0.8±0.1	13.5±2.4	56±1	6.6±0.7	1.6±0.2	44±1	8.1±1.7
14	1(11),2(6),3(8),4(10)	0.8±0.0	13.0±1.0	52±1	8.0±1.2	1.4±0.1	48±1	7.4±0.7
**eGFP::Bcd^{N51A} PN: 3D-Diffusion 2-particle model (30s each measurement)**								
12	1(6),2(7),3(3),4(3),5(4)	0.7±0.0	15.8±1.5	69±5	7.0±1.2	1.7±0.4	31±5	11.0±1.3
13	1(11),2(10),3(5), 4(5),5(6)	0.7±0.1	16.0±2.2	67±5	7.0±1.8	1.5±0.2	33±5	11.0±2.0
14	1(7),2(12),3(8),4(6),5(9)	0.6±0.1	17.0±3.2	64±6	7.0±1.2	1.6±0.2	36±6	11.0±2.0
**eGFP::Bcd^{N51A} AC: 3D-Diffusion 2-particle model (60s each measurement)**								
12	1(4),2(2),3(1),4(2)	1.0±0.2	10.7±2.7	64±6	14.1±4.3	0.8±0.3	36±6	7.1±1.3
13	1(6),2(6),3(5),4(6)	1.0±0.3	10.7±2.4	64±3	13.4±4.3	0.9±0.3	36±3	7.0±1.4
14	1(6),2(7),3(8),4(8)	0.9±0.3	12.3±2.7	64±2	11.8±5.4	1.0±0.3	36±2	8.0±1.7
**eGFP::Bcd^{N51A} PC: 3D-Diffusion 2-particle model (60s each measurement)**								
12	1(4),2(2),3(4)	0.6±0.1	16.9±0.6	79±7	9.5±1.7	1.2±0.3	20±7	13.5±1.6
13	1(6),2(6),3(4),4(8),5(7)	0.6±0.0	18.5±2.8	79±3	9.7±2.5	1.3±0.5	21±3	14.7±2.2
14	1(6),2(4),3(6),4(6)	0.6±0.0	18.3±1.1	83±6	11.3±1.7	1.1±0.3	17±6	15.2±1.2
**eGFP::Bcd^{R54A} AN: 3D-Diffusion 2-particle model (30s each measurement)**								
12	1(5),2(2),3(5)	1.2±0.2	9.3±0.4	59±3	37.0±8.0	0.4±0.1	41±3	5.5±0.0
13	1(5),2(4),3(9)	1.4±0.2	8.1±1.3	55±1	57.8±15.0	0.3±0.1	45±1	4.5±0.7
14	1(9),2(12),3(7)	1.3±0.2	8.5±1.6	55±2	40.1±10.0	0.4±0.1	45±2	4.8±1.1
**eGFP::Bcd^{R54A} PN: 3D-Diffusion 2-particle model (30s each measurement)**								
12	1(5),2(3),3(7)	0.7±0.1	14.4±3.0	70±5	19.0±5.8	0.7±0.2	30±5	10.2±1.9
13	1(9),2(5),3(7)	0.7±0.0	15.2±1.0	70±2	16.0±1.0	0.7±0.1	30±2	10.7±0.7
14	1(13),2(8),3(9)	0.7±0.0	16.0±1.0	69±1	18.0±4.0	0.7±0.1	31±1	11.1±0.6
**eGFP::Bcd^{R54A} AC: 3D-Diffusion 2-particle model (60s each measurement)**								
12	1(5),2(3),3(2),4(2)	0.9±0.1	11.4±1.7	66±4	10.5±1.6	1.0±0.1	34±4	7.8±1.2
13	1(10),2(7),3(8),4(5)	1.0±0.2	11.3±1.8	66±3	10.0±3.0	1.1±0.3	34±3	7.7±1.3
14	1(10),2(13),3(3),4(11)	0.9±0.1	11.8±0.7	67±7	9.7±2.0	1.2±0.2	33±7	8.2±0.2
**eGFP::Bcd^{R54A} PC: 3D-Diffusion 2-particle model (60s each measurement)**								
12	1(4),2(4),3(3),4(4)	0.8±0.2	13.8±2.4	84±6	21.3±5.0	0.6±0.1	16±6	12.5±1.5
13	1(10),2(3),3(8),4(7)	0.8±0.1	13.8±2.6	86±2	22.0±5.9	0.7±0.2	14±2	11.8±2.3
14	1(7),2(3),3(7),4(4)	0.6±0.1	17.6±2.9	83±5	15.0±10.7	1.0±0.5	17±5	14.2±1.9

Development • Supplementary information

Table S5. Comparison of parameter values of eGFP::bcd^{MM} diffusion fit using 3D -2-particle diffusion model.

N.C.	Embryo No. (no. of ACF curves)	TauD$_1$ (ms)	D1 (μm^2/s)	Fraction (F$_1$%)	TauD$_2$ (ms)	D$_2$ (μm^2/s)	Fraction (F$_2$%)	Eff.Diff. (μm^2/s)
**eGFP:: bcd^{MM} AN : 3D-Diffusion 2-particle model**								
12-14	1(9), 2(8)	0.8±0.3	13.5±6.6	63±10	10.8±6.3	1.2±0.5	37±10	8.5±3.3
**eGFP:: bcd^{MM} PN : 3D-Diffusion 2-particle model**								
12-14	1(3), 2(8)	0.4±0.2	20.0±5.2	78±9	6.2±2.6	1.3±0.5	22±9	15.7±3.5
**eGFP:: bcd^{MM} AC : 3D-Diffusion 2-particle model**								
12-14	1(21), 2(9)	0.7±0.2	13.0±2.8	61±9	8.5±2.4	1.0±0.3	39±9	8.3±2.0
**eGFP:: bcd^{MM} PC : 3D-Diffusion 2-particle model**								
12-14	1(3), 2(7)	0.4±0.1	22.6±7.2	78±9	6.3±3.9	1.5±0.8	22±9	16.9±4.9

Table S6. Comparison of parameter values of eGFP::NLSbcd^{HD} diffusion fit using 3D -2-particle diffusion model.

N.C.	Embryo No. (no. of ACF curves)	TauD$_1$ (ms)	D1 (μm^2/s)	Fraction (F$_1$%)	TauD$_2$ (ms)	D$_2$ (μm^2/s)	Fraction (F$_2$%)	Eff.Diff. (μm^2/s)
**eGFP::NLSbcd^{HD} AN : 3D-Diffusion 2-particle model (30s each measurement)**								
12	1(2), 2(1)	2.3±0.1	4.3±0.2	49±1	55.6±33.3	0.2±0.1	51±1	2.2±0.2
13	1(10), 2(2)	1.2±0.3	9.0±2.2	52±13	64.3±53.8	0.3±0.3	48±13	4.7±1.5
14	1(12), 3(2)	0.8±0.3	13.3±4.3	53±11	19.0±8.8	0.6±0.3	47±11	7.2±2.2
**eGFP::NLSbcd^{HD} PN : 3D-Diffusion 2-particle model (30s each measurement)**								
12	1(5), 2(1)	0.7±0.1	14.8±2.7	44±13	19.3±5.9	0.6±0.2	56±13	6.7±1.2
13	1(10), 2(2)	0.8±0.3	14.0±4.6	51±16	18.2±6.1	0.6±0.2	49±16	7.3±2.6
14	1(7), 2(2)	1.3±0.3	8.5±2.4	54±7	25.2±9.8	0.4±0.0	46±7	5.1±0.2
**eGFP::NLSbcd^{HD} AC : 3D-Diffusion 2-particle model (60s each measurement)**								
12	1(4), 2(1)	1.0±0.3	10.6±2.5	46±13	8.5±2.4	1.3±0.4	54±12	5.3±0.3
13	1(7), 2(2)	0.8±0.3	14.0±4.4	39±8	7.3±2.3	1.4±0.3	61±8	6.2±1.6
14	1(7), 2(2)	1.0±0.2	10.7±2.4	50±5	8.2±0.9	1.2±0.1	50±4	6.0±1.0
**eGFP::NLSbcd^{HD} PC : 3D-Diffusion 2-particle model (60s each measurement)**								
12	1(3), 2(1)	0.9±0.0	10.7±0.8	57±0	7.8±1.4	1.3±0.2	43±0	6.7±0.4
13	1(6), 2(2)	1.0±0.2	10.3±2.0	59±7	12.6±9.1	1.0±0.5	41±7	6.4±1.0
14	1(9), 2(2)	1.0±0.3	11.4±3.8	59±10	9.2±3.7	1.2±0.4	41±10	7.2±2.2

Table S7. Table of models considered with fitting parameters.

Model	Brief Description	Fixed Parameters	Fitted parameters	Equations
**1**	SDD	$\nu = 1/50 mins^{-1}$	$D = 2.9 \pm 0.1 \mu m^2 s^{-1}$<br>$\mu = 1/49 \pm 1/98\ mins^{-1}$<br>$J = 86 \pm 3\ \mu m^{-2} s^{-1}$	[1-3]
**2**	SDD with source domain	$\nu = 1/50 mins^{-1}$	$D = 2.8 \pm 0.2 \mu m^2 s^{-1}$<br>$\mu = 1/48 \pm 1/96\ mins^{-1}$<br>$J = 3.1 \pm 0.1\ \mu m^{-3} s^{-1}$<br>$x_0 = 28 \pm 2 \mu m$	[4]
**3**	2-component with no spatial dependence	$\nu = 1/50 mins^{-1}$<br>$D_{slow} = 1 \mu m^2 s^{-1}$<br>$D_{fast} = 12 \mu m^2 s^{-1}$	$\alpha = 0.02 \pm 0.004 s^{-1}$<br>$\beta = 0.07 \pm 0.01 s^{-1}$<br>$J = 2.9 \pm 0.6 \mu m^{-3} s^{-1}$<br>$\mu = 0.0003 \pm 0.0001 s^{-1}$<br>$x_0 = 28 \pm 2 \mu m$	[5]
**4**	2-component with spatial variation only in diffusion coefficients	$\nu = 1/50 mins^{-1}$<br>$D_{1,ant} = 12 \mu m^2 s^{-1}$<br>$D_{1,pos} = 18 \mu m^2 s^{-1}$<br>$D_{2,ant} = 0.5 \mu m^2 s^{-1}$<br>$D_{2,pos} = 1 \mu m^2 s^{-1}$	$\alpha = 0.08 \pm 0.03 s^{-1}$<br>$\beta = 0.03 \pm 0.01 s^{-1}$<br>$J = 9.1 \pm 2.5 \mu m^{-3} s^{-1}$<br>$\mu = 0.0006 \pm 0.0002 s^{-1}$<br>$x_0 = 27 \pm 4 \mu m$	[5-6]
**5**	2-component with spatial dependence in diffusion and β	$\nu = 1/50 mins^{-1}$<br>$D_{1,ant} = 12 \mu m^2 s^{-1}$<br>$D_{1,pos} = 18 \mu m^2 s^{-1}$<br>$D_{2,ant} = 0.5 \mu m^2 s^{-1}$<br>$D_{2,pos} = 1 \mu m^2 s^{-1}$	$\alpha = 0.11 \pm 0.01 s^{-1}$<br>$\beta = 0.04 \pm 0.01 s^{-1}$<br>$J = 7.5 \pm 1.4 \mu m^{-3} s^{-1}$<br>$\mu = 0.0006 \pm 0.0001 s^{-1}$<br>$x_0 = 31 \pm 3 \mu m$<br>$x_1 = 208 \pm 30\ \mu m$	[5-7]

Development • Supplementary information

Supplementary Materials and Methods

Additional FCS Calibration Information

Calibration of FCS measurements:

We calibrated eccentricity of the confocal volume, κ before each round of measurements using the reference dye Atto-488 (Atto-Tec) that has a diffusion coefficient 400 $\mu m^2/s$ at room temperature (Kapusta, 2010). The effective volume of calibration (V_{eff} , from picoquant) was found to be 0.25±0.04fl and the value of κ was found to be 5.6±0.9. The value for κ was fixed at 5.6.

Additional details on FCS curve Fitting

The 3D model involving diffusion of 2 species was selected as it provided a good fit determined through quality of the residuals of each plot (Abu-Arish et al., 2010). Photophysical processes, *e.g*., triplet transitions (Atto488) or photoisomerisation and protonation kinetics (eGFP) at short times (Widengren et al., 1999) are a concern when estimating accurate diffusion times. The error rate in the measurement of the diffusion time becomes worse if the characteristic times of photophysical processes are large enough to overlap with the diffusion time. Further, since the total fluorescence of eGFP:Bcd in the anterior and posterior cytoplasm is lower compared to the nuclei, the noise in these curves further limits the distinction between photophysics and diffusion, reducing the accuracy of the determined τ_D values.

Therefore, we fitted our FCS curves with different time ranges to include or exclude the photophysical processes at short times. A lag time range of 0.001ms to 1s was considered for fits including photophysics (Figure S2) and 0.1 ms to 1 s for fits excluding photophysics (Table 2 and 3). The photophysical parameters, denoted for simplicity as τ_{trip} for the characteristic time and F_{trip} for the fraction, were allowed to vary. The distribution of τ_{D1} and τ_{D2} values with respect to their individual ttrip values are plotted and weighted means are calculated (Fig. S2B-E). Our estimation reveals comparable photophysics parameters values across the A-P axis (Fig. S12). The majority of the $\tau_{t\ riplet}$ values range from 30 to 100µs comparable to the characteristic times of eGFP measured previously (Haupts et al., 1998; Jimenez-Banzo et al., 2008). The fraction ranges from 0.15 to 0.22 (Fig. S12). The data with anomalies due to photobleaching or sudden jumps in the intensities due to movement of the nucleus during measurements, were excluded from evaluation.

Additional details on the concentration estimation of eGFP:Bcd

Determination of bicoid concentration in the posterior domain of the embryo

The eGFP::Bcd concentration in the posterior domain of the embryo become very low. Determining the accurate eGFP:Bcd concentration is a challenge. In the Anterior-posterior gradient profile, the quantification of the eGFP:Bcd nuclear intensity compared to that of the His::mcherry autofluorescence measured using 488 laser line reveals the signal is equivalent to the background noise in the posterior domain of the eGFP:Bcd embryo (Figure S13A). Further upon measurement of background noise using FCS we found the counts are ~4000-8500 per second in the cytoplasm and nuclear compartments of anterior and posterior domains. The signal is stronger over noise in the anterior nuclei (~60000cps) and cytoplasm (~10000cps) of the Bcd::eGFP embryos (Figure S13B). Therefore, it is reasonable to assume that the number of the molecules (N) in the anterior domain is $N = 1/G(0)$. In the case of posterior domain, both nuclear and cytoplasm counts are within ~10000 and therefore direct determination of the number of molecules from G(0) is complicated (Figure S13B). In the posterior nuclei, the signal to noise is better than the cytoplasm as suggested by the G(0) which above that of anterior nuclei (Figure S13C). For posterior cytoplasm, the concentration is estimated in figure 2A from the curves that are measured just below the midline of the embryo where the signal-to-noise is better than the posterior most. On the other hand, ACF curves of the background noise (H2b::mcherry) is uncorrelated whereas the bcd::eGFP in the nuclei and cytoplasm of the posterior domain are autocorrelated upto the lag time of 0.1ms (Figure S13C). Normalization of the ACF curves $[(G(t) - G(\infty))/(G(0) - G(\infty))]$ of nuclear and cytoplasmic compartments of anterior and posterior domains reveal differential mobility of Bcd::eGFP (Figure S13D). Further, we tested differential changes in dynamics of cytoplasmic bicoid at different location along the anterior-posterior axis of the embryo at n.c.13 (Figure S13E and S13F). We noticed that the correlation amplitude (G(0)) drops down as we go down the concentration gradient, suggesting the signal become very low towards the posterior most (Figure S13E) and normalized data with 2-P fit (Figure S13F) reveal the changes in bicoid dynamics along the A-P axis.

Development • Supplementary information

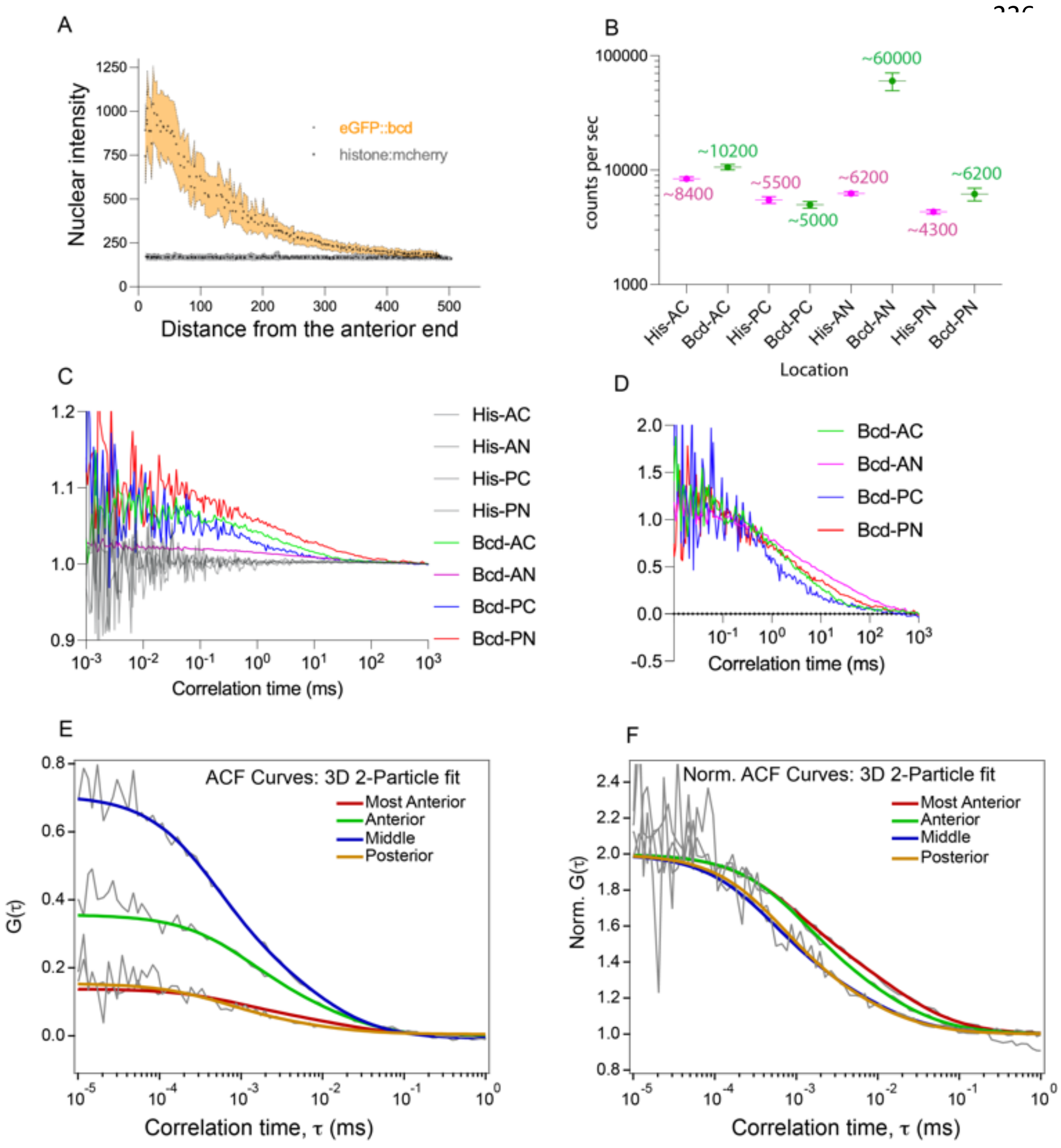

Fig. S13 (related to Figs. 1,2 and S1). Determination of Background noise and spatial variation in eGFP::Bcd diffusion in the early embryo

(A)Nuclear gradient profile of eGFP::Bicoid (orange) and histone mcherry (grey) from the anterior end of the blastoderm at n.c.14. (B) Photon counts per second measured from the n.c.14 embryos of eGFP::Bicoid (Green) and histone mcherry (magenta) from the cytoplasmic and nuclear compartments of anterior and posterior location. (C) ACF curves of Histone-mcherry (grey) compared to eGFP:Bicoid. Note the uncorrelated background noise from Histone-mcherry embryos. (D) Normalized ACF curves of eGFP:Bicoid from (C). (E) ACF curves (grey) and 3D 2-Particle fits without considering triplet state (colored) from 0.0001 ms to 1s from the cytoplasm measurements taken from equally distant location from the embryo's anterior end at early n.c. 13. Note the drop in the amplitude from the ACF curves of posterior most domain. (F) Normalized ACF curves from E.

Development • Supplementary information

Details on SPIM based Imaging-FCS

The setup included an illumination and detection arm placed orthogonal to each other. A 488 nm diode laser line (Cobolt 06-MLD 488nm 0488-06-01-0100-100, Cobolt AB, Sweden) was used to excite eGFP. The laser beam was directed through an optical fibre (kineFLEXP-3-S-405..640-1.0-4.0-P2, Qioptiq, United States) into the illumination arm consisting of an achromatic cylindrical lens (f =75 mm; ACY254-075-A; Thorlabs Inc., United States) and an illumination objective (SLMPLN 20 × /0.25 NA; Olympus, Japan). The optical fibre expanded the beam sufficiently to overfill the back aperture of the illumination objective, and the cylindrical lens-objective pair was used to generate a static diffraction-limited light sheet.

On the detection arm, a detection objective (LUMPLFLN 60×/1.0 NA; Olympus, Japan) was housed in a custom-built sample chamber of dimensions 3 cm x 3 cm x 3 cm with a coverslip facing the illumination objective side and an opening on top for mounting the sample. The detection objective was coupled with a piezo flexure objective scanner (P-721 PIFOC; Physik Instruments, Germany) for fine control of the position of the detection objective with respect to the light sheet while focusing (100 nm step size). The signal captured by the objective was passed through a filter (FF03-510/20-25 Semrock, United States) to capture only the eGFP's fluorescence signal. The signal was then focused onto an EMCCD camera (Andor iXon3 860, 128 × 128 pixels; Andor Technology, United Kingdom) by a tube lens (LU074700, f = 180 mm, Olympus, Japan). The illumination and detection arms were aligned to ensure that the light sheet coincided with the focal plane of the detection objective and that the signal from the thinnest section of the light sheet was captured at the centre of the camera.

eGFP::Bcd embryos were mounted in a Fluorinated Ethylene Propylene (FEP) tube of 1.1 × 1.5 mm^2 cross-section (FT 1.1 × 1.5, Adtech Polymer Engineering, England, United Kingdom) filled with 1% agarose (UltraPureTM Low Melting Point Agarose, 16520100, Thermofisher Scientific, United States) and positioned in a way that the anterior tip pointed into one of the walls of the tube. The FEP tube was held by self-closing forceps and mounted on a motorised stage with three linear positioning systems (Q-545 Q-MotionR Precision Linear Stage; Physik Instruments, Germany) with piezo motors for the three-axis and one rotation stage (DT-34 Miniature Rotation Stage; Physik Instruments, Germany). The FEP tube was placed in the sample chamber filled with water, and the tube was rotated to orient the anterior tip of the embryo to point towards the illumination objective to image the anterior margin with minimal illumination signal losses.

Since the position of the light sheet is fixed with respect to the detection objective, the embryo was imaged by moving the sample tube relative to the light sheet. The embryo was positioned such that the signal from the anterior margin was captured by the central region of the camera. This ensured that the

Development • Supplementary information

thinnest section of the light sheet illuminated this embryo region. A laser intensity of 30 W/cm^2 was used to illuminate the embryo for Imaging-FCS measurements.

For the Imaging FCS results in Figure S3, a time series image stack with 200,000 frames was recorded with an exposure time of 1 ms. The measurement was done in n.c. 13 to capture sufficient cytoplasm space. Since the nuclei moved during the measurement, only the first 80,000 frames were used to ensure that the nuclei stayed within the chosen ROI.

The image stack was analysed using an ImageJ plugin Imaging FCS 1.52 (Sankaran et al., 2021). Polynomial order 6 bleach correction was used. The fitting model used was:

$$G(\tau) = \frac{1}{N}\frac{g(\tau)}{g(0)}$$

where,

$$g(\tau) = \left\{\frac{\sqrt{4D\tau + \omega_{xy}^2}}{\sqrt{\pi}\cdot a} \times \left[e^{\left(-\frac{a^2}{4D\tau+\omega_{xy}^2}\right)} - 1\right] + \operatorname{erf}\left(\frac{a}{\sqrt{4D\tau + \omega_{xy}^2}}\right)\right\}^2 \times \left(1 + \frac{4D\tau}{\omega_z^2}\right)^{-\frac{1}{2}} + G_\infty$$

where a is the pixel size, τ is the lag time, N is the number of particles, D is the diffusion coefficient, ω_{xy} is the 1/e^2 radius of the PSF in the xy direction, and ω_z is the 1/e^2 radius of the thickness of the light sheet. The value for the pixel size used for fitting was 400 nm, the value ω_{xy} used was 721 nm, and the value of ω_z was 1.13 µm and the fit parameters of D and N were recorded for every pixel to create the spatial D and the N maps.

Development • Supplementary information

Additional Model Details

Models were solved in Matlab. Parameter optimisation was done using *fminsearch*. For each model, we performed 100 parameter minimisations, where we randomly sampled the experimental profile each time to generate distinct profiles (all within experimental error). Fitting was done by minimising the least square error. This was done both on the data values (which tended to more heavily weigh larger values, nearer the anterior) and on the logarithm of the data values (which tended to more heavily weigh smaller values, nearer the posterior). Both techniques gave similar results for the parameters, though the former better fit the profile in the anterior and the latter in the posterior.

Model 1: SDD model

First, we considered the standard SDD model, with point source at $x = 0$.

$$\frac{\partial\rho(x,t)}{\partial t} = D\frac{\partial^2\rho(x,t)}{dx^2} - \mu\rho \qquad [1]$$

with boundary constraints

$$D\frac{\partial\rho(x=0,t)}{dx} + J = 0, \frac{\partial\rho(x=L,t)}{\partial x} = 0$$

But we need to account for folding of Bicoid at rate ν:

$\rho_{total}(x,t) = \rho_{unfolded}(x,t) + \rho_{folded}(x,t)$, where

$$\frac{\partial\rho_u(x,t)}{\partial t} = D\frac{\partial^2\rho_u(x,t)}{dx^2} - (\mu+\nu)\rho_u(x,t) \qquad [2]$$

$$\frac{\partial\rho_f(x,t)}{\partial t} = D\frac{\partial^2\rho_f(x,t)}{dx^2} + \nu\rho_u(x,t) - \mu\rho_f(x,t) \qquad [3]$$

with boundary constraints

$$D\frac{\partial\rho_u(x=0,t)}{dx} + J = 0, \frac{\partial\rho_u(x=L,t)}{\partial x} = 0, \frac{\partial\rho_f(x=0,t)}{dx} = 0, \frac{\partial\rho_f(x=L,t)}{\partial x} = 0$$

This model is shown in Figure 2C.

Model 2: SDD model with distributed source

We can adapt Model 1 to include an extended source. For simplicity, we define the source as J if $x<x_0$ and zero otherwise. x_0 is left as a fitting parameter in the simulations.

The equations are as Model 1, except now equation [2] becomes

$$\frac{\partial\rho_u(x,t)}{\partial t} = D\frac{\partial^2\rho_u(x,t)}{dx^2} - (\mu+\nu)\rho_u(x,t) + J \text{ if } x < x_0 \qquad [4]$$

and $\frac{\partial\rho_u(x,t)}{\partial t} = D\frac{\partial^2\rho_u(x,t)}{dx^2} - (\mu+\nu)\rho_u(x,t)$ if $x \geq x_0$

with boundary condition $\frac{\partial\rho_u(x=0,t)}{dx} = 0$

Development • Supplementary information

Model 3: Two component model with no spatial variation

We next consider a two-component model, where the diffusion coefficients do not vary across the embryo. We assume that Bicoid is in the fast form immediately after translation. We denote the two Bcd species by 1 and 2, denoting fast and slow forms respectively. We include a source domain region defined by x_0. The switching rate between fast and slow forms (denoted by α and β) are fitted constants, that do not vary across the embryo.

$$\frac{\partial \rho_{u,1}(x,t)}{\partial t} = D_1 \frac{\partial^2 \rho_{u,1}(x,t)}{dx^2} - (\mu + \nu + \beta)\rho_{u,1}(x,t) + \alpha\rho_{u,2}(x,t) + J \text{ if } x < x_0$$
$$\frac{\partial \rho_{u,1}(x,t)}{\partial t} = D_1 \frac{\partial^2 \rho_{u,1}(x,t)}{dx^2} - (\mu + \nu + \beta)\rho_{u,1}(x,t) + \alpha\rho_{u,2}(x,t) \quad \text{ if } x \geq x_0 \qquad [5]$$

$$\frac{\partial f_{f,1}(x,t)}{\partial t} = D_1 \frac{\partial^2 \rho_{f,1}(x,t)}{dx^2} - (\mu + \beta)\rho_{f,1}(x,t) + \nu\rho_{u,1}(x,t) + \alpha\rho_{f,2}(x,t)$$

$$\frac{\partial \rho_{u,2}(x,t)}{\partial t} = D_2 \frac{\partial^2 \rho_{u,2}(x,t)}{dx^2} - (\mu + \nu + \alpha)\rho_{u,2}(x,t) + \beta\rho_{u,1}(x,t)$$

$$\frac{\partial \rho_{f,2}(x,t)}{\partial t} = D_2 \frac{\partial^2 \rho_{f,2}(x,t)}{dx^2} - (\mu + \alpha)\rho_{f,2}(x,t) + \beta\rho_{f,1}(x,t)$$

Model 4: Two component model with spatial variation only in diffusion

This version is as Model 3, except the first term on RHS is adapted to $\frac{\partial}{\partial x}\left(D_s(x)\frac{\partial}{\partial x}\rho_s(x,t)\right)$ where we take

$$D_s(x) = D_s(anterior) + (D_s(posterior) - D_s(anterior))\frac{x}{L} \qquad [6]$$

so that $D_s(0) = D_s(anterior)$ and $D_s(L) = D_s(posterior)$. This is a simple approximation to introduce spatially varying diffusion coefficients. Here, L denotes the length along the AP axis, typically taken to be 500μm.

Model 5: Two component model with spatial variation in diffusion and β Same as Model 4, except now

$$\beta(x) = \beta_0 \cdot \left(1 + \right) \quad \frac{x}{x + x_1} \qquad [7]$$

so x_1 is an additional fitting parameter. This is shown in Figure 3.

Supplementary References

Abu-Arish, A., Porcher, A., Czerwonka, A., Dostatni, N. and Fradin, C. (2010). High mobility of bicoid captured by fluorescence correlation spectroscopy: implication for the rapid establishment of its gradient. *Biophys J* **99**, L33-35.

Haupts, U., Maiti, S., Schwille, P. and Webb, W. W. (1998). Dynamics of fluorescence fluctuations in green fluorescent protein observed by fluorescence correlation spectroscopy. *Proc Natl Acad Sci U S A* **95**, 13573-13578.

Jimenez-Banzo, A., Nonell, S., Hofkens, J. and Flors, C. (2008). Singlet oxygen photosensitization by EGFP and its chromophore HBDI. *Biophys J* **94**, 168-172.

Kapusta, P. (2010). Absolute Diffusion Coefficients:Compilation of Reference Data for FCS Calibration. *PicoQuant*.

Sankaran, J., Balasubramanian, H., Tang, W. H., Ng, X. W., Rollin, A. and Wohland, T. (2021). Simultaneous spatiotemporal super-resolution and multi-parametric fluorescence microscopy. *Nat Commun* **12**, 1748.

Widengren, J., Mets, Ü. and Rigler, R. (1999). Photodynamic properties of green fluorescent proteins investigated by fluorescence correlation spectroscopy. *Chemical Physics* **250**, 171-186.
