# Supplementary material for: Long-range formation of the Bicoid gradient requires multiple dynamic modes that spatially vary across the embryo
Source: Development. 2024 Feb 12;151(3):dev202128. doi: 10.1242/dev.202128 (PMC10911119; doi:10.1242/dev.202128)
Supplement: Supplementary file 1 [file develop-151-202128_review_history.pdf]

Long-ranged formation of the Bicoid gradient requires multiple dynamic modes that spatially vary across the embryo

Thamarailingam Athilingam, Ashwin VS Nelanuthala, Catriona Breen, Thorsten Wohland and Timothy Saunders
DOI: 10.1242/dev.202128

Editor: Paul Francois

Review timeline

Submission to Review Commons:	31 March 2023
Original submission:	26 June 2023
Editorial decision:	7 July 2023
First revision received:	29 November 2023
Accepted:	3 January 2024

Reviewer 1

Evidence, reproducibility and clarity

This manuscript reports a very thorough and careful study of the mobility of Bicoid in the early embryo, explored with single-point fluorescence correlation spectroscopy. Although previous groups have looked into this question in the past, the work presented here is novel and interesting because of the different Bicoid mutants and constructs the authors have examined, in particular with the goal of understanding the role of the protein DNA-binding homeodomain. The authors convincingly show that there is a significant increase in Bicoid dynamics from the anterior to the posterior region of the embryo, and that the homeodomain plays an important role in regulating the protein's dynamics. Their experiments are very well designed and carefully analyzed. The authors also modelled gradient formation to see whether this change in dynamics might play a role in setting the shape of the gradient. I am not sure I fully agree with their conclusion that it does, as mentioned in my comment below. However, it is an interesting discussion to have, and I think this paper makes a significant advance in our understanding of Bicoid's behavior in the early embryo.

Major comments:

1. Gradient profile quantification: Some of the conclusions made by the authors rely on the comparison between their model of gradient formation (as captured in the equations in lines 232 and 233) and the Bcd intensity profile measured in the embryos. Since the differences in gradient shape predicted by the different models are very small (see Fig. 3B, which is on a log scale and therefore emphasize small differences, and Fig. 3C), it is very important to understand how reliable the experimental concentration profiles are. At the moment, I do not find the evidence that [Bcd] concentration profile is more consistent with a 2-component diffusion model than a 1-component model very strong. A few comments related to this:
- 1a. Line 249, it is mentioned that: "observations ... incompatible with the SDD model". Which observations exactly are incompatible with the SDD model?
- 1b. In Fig. 3D, only the prediction of the 2-component model is shown. What would the simple 1-component diffusion model look like? Is it really incompatible with the data?
- 1c. Line 243: "The increased fraction in the fast form ... consistent with experimental observation of Bcd in the most posterior" (Mir et al.)". I am not sure how this is significant, since the simple model also predicts there will be Bcd in the posterior - the only difference is how much is there (as shown in Fig. 3C), and it's a very small difference.

© 2024. Published by The Company of Biologists under the terms of the Creative Commons Attribution License (https://creativecommons.org/licenses/by/4.0/).

- 1d. Since the difference between models is in the posterior region where Bcd concentration is very low, when comparing the models to the data the question of background subtraction is essential. How was the subtracted background (mentioned line 612) estimated?
- 1e. Along the same line, were the detectors on the Zeiss LSM analog or photon counting detectors, and how confident can we be that signal is exactly proportional to concentration?
- 1f. Can the gradients created by the two Bcd mutants (FIg. 4B) be quantified as well, and are they any different from the original Bcd gradient?
- 1e. What is the pink line in Figure 5C (I am assuming the green one is the same as in Fig. 3D)? It could be better to not use normalization here, or normalize everything respective to the eFP::Bcd data to make comparison in relative concentrations in the posterior for different constructs more evident (also maybe different colors for the three different data sets would help clarity).
- 1f. Discussion, lines 402-403: Does the detailed shape of the Bcd in the posterior region matter at all, since the posterior is not a region where Bicoid is active, as far as we know? Could a varying Bcd dynamics have other consequences that would be more biologically relevant?

2. Model for gradient formation (lines 231-238):
- 2a. Whether the molecules of Bcd can change from their fast to slow form is never questioned. How do we know (or why might we suspect) they do exchange?
- 2b. The values used in the model for alpha, beta_0 and rho_0 should be mentioned. Maybe having a table with all the parameters in the method section, or even in the supplementary section, would help. The exact values of alpha and beta matter, because if they are large (fast exchange) a single exponential gradient is to be expected, if they are 0 (no exchange) a double exponential gradient is to be expected, with intermediate behavior in between. Which case are we in here?

3. Discussion about anomalous diffusion (lines 386-388): The 2-component model used by the authors to interpret their FCS data seems very well justified here (excellent fits with very small residuals). I agree with the authors' conclusion that "the dynamics of Bcd within the nucleus are more complicated than a simple model of bound versus unbound Bcd", but I don't see how that can lead to a diagnostic of anomalous diffusion instead. Maybe it is just a matter of exactly explaining what is meant by anomalous diffusion here (since this term is often used to mean different things). A more likely scenario I think, is that there are more than just two Bcd components in the system.

4. Line 440 and after: What is the evidence that the transition between the two forms might vary non-linearly with Bcd concentration? How would that help adapt to different embryo sizes? It would be good to be more explicit here instead of just referring to another paper.

5. Since an important aspect of this work is the study of different Bcd constructs in vivo, it is important that these constructs are very clearly described, so the section on the generation of the fly lines (Methods) should be expanded. In particular:
- 5a. It seems that the eGFP:: NLS control used here was different from that first described in Ref. 64 (and used for FCS experiments in Ref. 30 and 36)? If so, what NLS sequence was used here, and precisely what type of eGFP was used (in particular, was the A206K mutation that prevents dimerization present in the eGFP used)? If it is the same construct as in Ref. 64, it should be mentioned explicitly.
- 5b. Were the mutant N51A and R54A lines gifts as well, or have they been described before? If so, previous publications should be referenced. If not, how the plasmid was introduced in the embryo should be briefly explained.

6. Concentration calibration measurements (Methods Fig. 2, line 568 and on). It is well known that background noise is going to interfere with the measurement of N when the signal becomes equivalent to the background noise (Koppel 197, Phys Rev A 10:1938-1945, and for a recent discussion of this effect for morphogens in fly embryos: Zhang et al., 2021, Biophysical Journal 120,4230-4241). It is almost certain that in the low signal regions of the embryo (e.g. posterior cytoplasm) this is affecting the reported concentration, and should be at least acknowledged.

7. Reference 3 is mis-characterized in two different ways in the manuscript:
- 7a. Line 50: The conclusion in Ref. 3 was not that the gradient was due to a diffusive process, on the contrary Gregor et al. argued that Bcd was too slow to form such a long- range gradient by diffusion. Studies that do present data consistent with a morphogen gradient formation mechanism driven by diffusion are reference 5, reference 30, Zhou et al., Curr. Biol. 2012;22(8):668-75 and Müller et al., Science 336 (2012) 721-724.
- 7b. The diffusion coefficient estimated from FRAP measurements and reported in Ref. 3 (D = 0.4 micron^2/s) is mentioned a couple of times in the manuscript (line 66, line 395, line 411). However, this number is simply incorrect. When fast components (such as the ones clearly

© 2024. Published by The Company of Biologists under the terms of the Creative Commons Attribution License (https://creativecommons.org/licenses/by/4.0/).

detected here by FCS) are present, they diffuse out of the photobleached area during the photobleaching step. If that is not corrected for during the analysis (and it wasn't in Ref. 3), then the recovery time measured is just equal to the photobleaching time, and has nothing to do with either the fast or slow fraction of the studied molecule - it has no other meaning than to give a lower bound on the value of the actual effective diffusion coefficient of the molecule. This effect (called the halo effect) is well known in the FRAP community (see e.g. Weiss 2004, Traffic 5:662-671), it has been experimental demonstrated to occur for Bcd-eGFP in the conditions used in Ref. 3 (Reference 30), and the actual diffusion coefficient that should have been extracted from the data presented in Ref. 3 has been recalculated by another group to be instead D = 0.9 micron^2/s (Castle et al., 2011, Cell. Mol. Bioeng. 4:116-121). It would therefore be better to report the corrected value from Castle et al. to help the field converge towards an accurate description of Bcd mobility.

Minor comments and suggestions:

8. Figure 1: From panel A, it seems that what is called "Anterior" and "Posterior" is about 150 micron away from the embryo mid-section, i.e. about 100 micron from either the anterior pole or the posterior pole (so not the tip of the embryo, but somewhere in the anterior half or posterior half). Maybe this should be made clear in the text.
9. Fig. 2A; It might be good to put this graph on a log scale, so that cytoplasmic values are seen more clearly. Also, what about reporting on nuclear to cytoplasmic ratios?
10. Fig. 2: It could be interesting to plot D_effective as a function of the measured concentration of Bicoid in different locations, since the (interesting) suggestion is made several time that [Bcd] could the a determinant of the protein mobility.
11. Figure 3B&C: Is the curve for 2-component diffusion (without concentration dependence) for steady-state missing?
12. Lines 78 and 471: What do the authors mean by "new reagents"? The word reagent evokes a chemical reaction, but there are none here. Do the authors mean new constructs? or new mutants?
13. Lines 57-59: Another good reference for FCS measurements performed to study the dynamics of a morphogen (in this case Dpp) is Zhou et al., Curr. Biol. 2012;22(8):668-75
14. Lines 109-111: A word must be missing. Precisely determined what?
15. Line 278: The increase in the slow mode is expected. Maybe explicitly mention why.
16. Line 282: "with the fast component increasing", maybe replace with "with the diffusion coefficient of the fast component increasing" or "with the fraction of the fast component increasing".
17. Line 517: Is there a reason why the dorsal surface is always placed in the coverslip?
18. Line 524 and on: FCS measurements: What was the duration of each individual FCS measurement? It is great that the exact number of measurements are reported in the supplementary!
19. An Airy unit of 120 um seems large in combination with an objective with a NA of 1.2, is there a reason for that? What was the radius of the resulting detection volume?
20. Thank you for detailing the reasons behind the choice of excitation power, an important and often omitted details. Where in the excitation path were the values of the laser power measured (before or after the objective?)?
21. Line 585: "since the brightness of eGFP::Bcd..." do the authors mean the molecular brightness of a single eGFP::Bcd molecule, or the total fluorescence signal?
22. It would be good for reference to mention the approximate value of the molecular brightness recorded for these eGFP constructs at the laser power used.
23. Reference 766: The year (and maybe other things) is missing.

Figure 2 (Methods): The concentrations shown on the figure should be in nM not uM.

Significance

Strengths:

Very careful and systematic study of Bcd's dynamics in the early embryo
Use of several mutant and truncated forms of Bcd to pinpoint the importance of the DNA binding domain in setting this dynamics
Uncovers a previously unknown change in Bcd dynamics from the anterior to the posterior of the embryo

© 2024. Published by The Company of Biologists under the terms of the Creative Commons Attribution License (https://creativecommons.org/licenses/by/4.0/).

Modelling of the Bcd concentration gradient shape taking into account the measured dynamics

Limitations:

The quantitative comparison between modelled and measured gradient could be improved. The discussion of the biological implications of the work is limited

Advance:

Uncovers a previously unknown change in Bcd dynamics from the anterior to the posterior of the embryo. This raises very interesting questions about molecular mechanisms involving Bicoid. Other studies (cited in this manuscript) reported on Bcd dynamics, but the present study represents a very welcome expansion of these earlier studies, by looking at spatial dependence and by examining several Bcd constructs.

Audience:

Somewhat specialized, as this work should firstly be of interest to scientists studying morphogen gradients. However, it is also a beautiful example dynamical studies in vivo, so it will also be of interest to experimental physicists studying protein motions in vivo (a rather large community).

Reviewer 2

Evidence, reproducibility and clarity

Summary:

In this study, Athilingam et al. investigated Bicoid (Bcd) protein dynamics along the anterior-posterior axis of Drosophila embryos. They performed Fluorescence Correlation Spectroscopy (FCS) experiments to analyze Bcd protein diffusion and found that Bcd had a 1.7-fold higher mobility in the posterior compared to the anterior. The authors also generated Bcd homeodomain mutations and analyzed their impact on gradient formation. They found that the BcdN51A mutation exhibited altered nuclear dynamics with faster diffusion, while cytoplasmic dynamics remained unchanged. BcdR54A embryos showed dynamics more similar to the wild-type Bcd, with a minor decrease in slow diffusion in the posterior region. Interestingly, the Bcd homeodomain alone, when fused to eGFP-NLS, was able to replicate the observed Bcd protein dynamics, particularly in the slower diffusive mode. The paper provides evidence that the Bcd homeodomain has a significant influence on protein dynamics and suggests a complex interplay of interactions between Bcd, nuclear DNA, and cytoplasmic RNA that regulate Bcd diffusion and function.

Overall, the data and methods presented in the paper are clearly described. The experiments are adequately replicated, and the statistical analysis appears sufficient. Prior studies are referenced appropriately, and the claims and conclusions are mostly supported by the data. However, the following points should be addressed to corroborate the conclusions:

Major points

1. FCS measurements and fits
- a) Please state the duration of each individual FCS measurement.
- b) The authors acknowledge potential issues with fluorophore photophysics and use different lag time ranges for the calibration dye Atto-488 (0.001 ms in Method Fig. 2) and eGFP (0.1 ms in the main figures). Given the strong influence of different parameters on data interpretation and conclusions, Method Fig. 2 should be repeated with purified eGFP. This is particularly relevant for the noisy FCS measurements in posterior regions.
- c) Please explain why no data is shown for "AN" around 0.1 ms lag time in Fig. 1B in contrast to all other figures.
- d) Please state what the estimated diffusion coefficients with one-component model fits are. Please also explain why the fits in Fig. S1E do not reach a value of 1 and why they plateau higher than the experimental data at long lag times. Please constrain the fits to G=1 at 0.1 ms tau and

© 2024. Published by The Company of Biologists under the terms of the Creative Commons Attribution License (https://creativecommons.org/licenses/by/4.0/).

G=0 at 1 s tau to make a fair comparison.
- e) Please assess the validity of all multi-component fits by comparing the relative quality of the models to the number of estimated parameters using the Akaike information criterion or similar approaches.
- f) Please also present the Bcd-GFP fits with 0.001 ms that are mentioned in line 590, and present the results for the data that did not give comparable tau_D1 and tau_D2 values mentioned in line 593.
2. Bicoid gradient and modeling
- a) Little et al. 2011 observed that the Bcd gradient decreases around n.c. 13. Can the authors of the present work observe a similar concentration decrease using FCS? This is important to i) validate the FCS concentration measurements, and ii) to resolve the controversy regarding "previous claims based on imaging the Bcd profile within nuclei, which predicted decrease in Bcd diffusion in later stages".
- b) Please explain why the experimental Bcd-GFP gradient data does not reach a value of 1 (e.g. in Fig. 3D) despite normalization. Please also explain why the fits become flatter in Fig. 5B compared to the steep fit in Fig. 3D.
- c) For modeling, please take into account observations that the Bcd source is graded with a wide distribution (30-40% EL, see Spirov et al. 2009, Little et al. 2011, Cai et al. 2017 etc.). The extent of the source used in the present work (x_s=20 um, line 620) is at least five times too small.
- d) Please discuss in the paper how well the simulations in Fig. 3B agree with the experimental data.
- e) Please provide a precise estimate for the statement "Even with an effective diffusion coefficient of 7 µm2s-1, few molecules would be expected at the posterior given the estimated Bcd lifetime (30-50 minutes)" to turn this into a quantitative argument. How many molecules are expected to reach posterior in which model, and how does it compare to experimental observations?
- f) The sentence "we find that a model of Bcd dynamics that explicitly incorporates fast and slow forms of Bcd (rather than a single "effective" dynamic mode) is consistent with a range of observations that are otherwise incompatible with the standard SDD model" needs to be toned down and corrected since a simple SDD appears to be sufficient to account for the observed gradients. If the authors disagree, please specifically point out in the paragraph around line 249 what observations exactly are incompatible with a standard SDD model.
3. Data presentation
- a) In line 27 and 122 it would be better to rephrase the wording "find/found" and give credit to previous papers that first made these observations.
- b) For the statement "This suggests that the dynamics of the fast fraction were not captured by previous FRAP measurements", please explain why this should not be the case even though the fast fraction is shown to be larger than the slow fraction in the current work.
- c) Similarly, the sentence "The dynamics of the slower mode correspond closely to measured Bcd dynamics from FRAP" likely needs to be corrected since it neglects the contribution of the faster mode, which is fluorescent as well and should also contribute to the dynamics from FRAP.
- d) In the absence of further evidence (see above), the sentences "We establish that such spatially varying differences in the Bcd dynamics are sufficient to explain how Bcd can have a steep exponential gradient in the anterior half of the embryo and yet still have an observable fraction of Bcd near the posterior pole" and "These results explain how a long- ranged gradient can form while retaining a steep profile through much of its range" in the abstract need to be toned down.
- e) The authors state that "However, we show that eGFP::Bcd in its fastest form can move quickly (~18 µm2s-1), and the fraction of eGFP::Bcd in this form increases at lower concentrations", but this has not been directly shown. Please tone down this statement or directly test the prediction that Bcd has a higher fraction of the fast form in earlier nuclear cycles when Bcd concentration is smaller.

Minor points

1. Introduction
- a) Please explain explicitly what exactly the contention in Bcd, Nodal and Wingless dynamics is in the cited references.
- b) In line 95, it would be better to state that this is a variation of the SDD model rather than "a new model".
2. Methods

© 2024. Published by The Company of Biologists under the terms of the Creative Commons Attribution License (https://creativecommons.org/licenses/by/4.0/).

- a) The authors state that "The same software was also used to calculate the cross- correlation function", but I couldn't find any cross-correlation analyses. Please clarify.
- b) Please correct the "uM" typo to "nM" in the legend of Method Fig. 2A.
- c) In the sentence "Further, since the brightness eGFP:Bcd in the anterior and posterior cytoplasm is lower compared to the nuclei", "brightness" probably needs to be changed to "concentration" since the molecular brightness is unlikely to change.
- d) Please explain the background-correction method mentioned in line 612. Please also state at what temperature the experiments were performed.

3. Results
- a) Please provide labels for anterior, posterior, dorsal and ventral in Fig. 1A.
- b) Please explain the colors in Fig. 5C.
- c) Please explain the dashed lines in Fig. 3C.

Optional

1. If possible, it would be helpful to mention whether the transgenic animals have any abnormal phenotypes or whether they can rescue the bcd mutant.
2. To validate the concentration measurements, it would be ideal if the authors could determine the Bcd concentration gradient using FCS along the anterior-posterior axis. This would also address whether there are further unexpected changes in diffusivity in medial regions and along the anterior-posterior axis that would have to be considered for modeling.
3. Local photoconversion experiments, e.g. in Bcd-Dendra2 embryos if available, would provide compelling support for the relevance of the measurements in the current work.

Significance

The paper investigates the diffusion dynamics of Bicoid (Bcd), a transcription factor crucial for establishing the anterior-posterior axis during Drosophila embryogenesis. The authors utilize Fluorescence Correlation Spectroscopy (FCS) and various Bcd mutants fused with eGFP to understand the role of Bcd's homeodomain and other domains in its nuclear and cytoplasmic diffusion dynamics. They performed elegant experiments using insightful transgenic lines, showcasing well-designed and well-executed methodology. The paper is very nice to read, with a clear and engaging writing style and excellent presentation of the data, making it easy to follow and understand their findings.

However, there are a few limitations to consider. First, the paper does not provide evidence for the switching between slow and fast populations central for their modeling, leaving an important aspect of the dynamics unexplained. Second, there are doubts regarding the accuracy of the model used to fit the FCS data (see detailed comments in the section "Evidence, reproducibility and clarity"), underscored by the statement "While the increase in the slow mode was expected, the reason for the change in the fast mode is less clear". Third, the relevance of a potentially higher Bcd mobility for gradient formation remains unclear. For example, the fit in Fig. 3D deviates substantially from the data around 0 um embryo length, which is likely even larger than the error expected from a "simple diffusion" fit at the posterior end of the embryo. In addition, in Fig. 3C the lines for the different models appear to be indistinguishable given the noisy measurements, calling the relevance of the findings into question.

Overall, the paper extends our knowledge by providing new insights into the role of the Bcd homeodomain in determining Bcd gradient formation. The paper highlights that homeodomain interactions in the cytoplasm and nuclei are significant contributors to determining Bcd dynamics. Additionally, the paper suggests that additional components within Bcd itself or other proteins in the cytoplasm affect Bcd dynamics at different Bcd concentrations. The paper will be of interest to a broad audience in developmental biology, molecular biology, and biophysics. Researchers studying transcription factor dynamics, morphogen gradients, and Drosophila embryogenesis will find this study particularly valuable. While the study is primarily focused on basic research, the insights gained on Bcd diffusion dynamics and the role of the homeodomain may contribute to a broader understanding of transcription factor regulation and function in other systems.

Reviewer 3

© 2024. Published by The Company of Biologists under the terms of the Creative Commons Attribution License (https://creativecommons.org/licenses/by/4.0/).

Evidence, reproducibility and clarity

The manuscript presents experiments that combine a number of techniques to produce an extremely detailed view of Bcd gradient formation in the early Drosophila embryo. The authors provide both empirical and theoretical evidence that support a conclusion that Bcd has both slow and fast moving forms, that the proportion of fast vs slow Bcd is higher at the posterior of the embryo, and that a theoretical model involving both fast and slow moving components fits empirical observations substantially better than a simple diffusion model. Next, they extend the work by investigating what functional motifs of Bcd are required for gradient formation. They demonstrate that a missense mutation N51A in the homeodomain (HD) that affects both DNA binding and cad regulation has major effects on Bcd gradient formation, while the R54A mutation that only affects cad regulation does not. They further show that a protein composed of only an NLS and the Bcd HD can form a gradient that is similar to full-length Bcd, although the two-component dynamics are not recapitulated by the HD-only form.

In my estimation the experimental work is rigorous and the results fully support the conclusions of the authors. I was surprised, however, that the HD-only form localizes via very different and simpler dynamics than does full-length Bcd, but nevertheless forms at least a qualitatively similar gradient. That leads to the question as to whether the existence of the fast and slow forms and their different ratios in different parts of the embryo actually are physiologically relevant. I don't see a straightforward way to test this experimentally, because the mutations that effect Bcd gradient formation also affect essential functions of the protein that if abrogated produce severe downstream effects on embryonic development and lethality. However I would like to see this point at least addressed in the discussion. The data and the methods are presented in such a manner that they can be reproduced, and the number of replicates and statistical analysis is overall robust.

Minor comments:

The presentation of the graphical data measuring Bcd levels along the a-p axis (Fig 1C, 1D, 4C-F and others) needs to be improved, because the grey lines that represent ACF curves are essentially invisible. This is partly because there is usually extensive overlap between the grey lines and other lines. This may be solved by using a more vivid colour than grey for the ACF curves, or perhaps the ACF lines could be made thicker but with some transparency so that overlapping data can be seen. In any event this aspect of the presentation needs to be improved.

In Figs 2D and 2I measurements of statistical significance between the proportion of protein in fast and slow modes need to be added.

Relevant to line 174 and Fig 2, NLS should be defined when first used, the source of the NLS should be given (is it from Bcd?) and the rationale for looking at eGFP::NLS should be made explicit.

In Fig 3D the dashed lines need to be defined. I assume these are experimental error bars but this is not stated.

On lines 344-5, shouldn't this conclusion concern the HD rather than the NLS?

On line 432, CAP is not an acronym, the correct term is 5' 'cap' or 'cap structure'. Also Cho et al. PMID 15882623 should be added to the references here.

On lines 446, 456, 469, and throughout: replace 'blastocyst' with 'blastoderm'. The former term is generally used for embryos that undergo full cellular divisions and cleavage in early embryogenesis, not for syncytial embryos such as Drosophila.

Significance

General assessment: The main strength of the paper is that it extends our knowledge about the dynamics of Bcd gradient formation, and by so doing it advances our understanding of the physical parameters underlying morphological gradients and patterning. The authors are admirably

© 2024. Published by The Company of Biologists under the terms of the Creative Commons Attribution License (https://creativecommons.org/licenses/by/4.0/).

open about questions that were unanswered by the study, which include identifying molecular interactions that affect Bcd dynamics in the cytoplasm, whether Bcd diffusion is dependent on its concentration, and whether other morphogens form gradients in a similar manner. Answering any of these questions would involve substantial experimental work and it is appropriate to leave these questions for subsequent manuscripts.

Advance: A number of high-profile papers were published on this topic in the late 2000s and early 2010s that reached difficult conclusions, so the nature of the mechanisms underlying Bcd gradient formation have remained controversial and somewhat opaque. While this paper does not provide a definitive answer to all relevant open questions, it nevertheless represents a significant advance toward their resolution.

Audience: This paper will be of interest both to developmental biologists interested in gradients and pattern formation, and to biophysicists interested in physical parameters affect molecular movements. It is fundamental research that does not have an obvious clinical or translational component.

Reviewer 4

Evidence, reproducibility and clarity

Summary:

Athilingam et al. are interested in understanding how the Bicoid (Bcd) morphogen gradient is formed in the early stages of Drosophila embryonic development. Using fluorescence correlation spectroscopy (FCS), the authors quantify the dynamics of Bcd in nuclei and cytoplasm at anterior and posterior regions of the embryo.
First, they characterize the dynamics of eGFP::Bcd in space and time. Analysing FCS autocorrelation curves at the anterior and posterior regions of the embryo during the interphases of nuclear cycles 12 to 14, they detect differences in Bcd diffusion and are able to infer and distinguish two Bcd populations with slow and fast diffusions. Moreover, these dynamics do not vary between nuclear cycles.
Using the different diffusions of the slow and fast populations, they find a model capable of explaining the formation of the gradient at larger distances and the existence of the Bcd molecules in the posterior, compatible with the SDD model widely accepted in the community.
Lastly, given that Bcd has multiple roles binding to DNA and also to RNA through its homeodomain, mutations affecting DNA/RNA and RNA only binding are used. Using a mutant without the ability to bind to DNA, their determine that the slower diffusion in the nuclei are due to DNA binding. They further confirm this by fusing the homeodomain of Bcd to eGFP::NLS.

Major comments:

The averaged autocorrelation curves were fitted to models of diffusion with one and two components. The one-component model was insufficient to reproduce the data and the two-component model seems to fit the data. Have the authors tested models with more than two components? Could it be possible to distinguish more Bcd populations?
In Figure 2E, the same concentration of eGFP::NLS is estimated to exist in the cytoplasm and nucleus. Since the NLS should target eGFP to the nucleus, what is the explanation for this observation? Is it possible that the method used to estimate the concentration of molecules is underestimating the concentration in the nucleus or the opposite in the cytoplasm?
In the simulation of the SDD model (Figure 3B), simulations at 10 min, 25 min and 120 min are shown. Assuming that 120 min corresponds to early nc14, are simulations at earlier timepoints corresponding to nc12 and nc13 indistinguishable from the profile at 120 min? This demonstration would further support the option to merge the data from all nuclear cycles.
The results obtained with the BcdN51A mutant show an increase in diffusion speed, while retaining similar proportions of fast and slow populations. In the slow fraction, a new population is found. Assuming that the BcdN51A molecules cannot bind specifically to DNA due to the mutation, what would this newly found population correspond to? Could the authors explore the possibility of nonspecific binding to DNA? The article would also win by discussing more on this aspect or other

© 2024. Published by The Company of Biologists under the terms of the Creative Commons Attribution License (https://creativecommons.org/licenses/by/4.0/).

options.
The experimental rational behind the BcdMM reporter needs to be better explained as it is not clear. It was previously shown that the N51A mutation disturbs zygotic hb activation and Caudal gradient formation (see Figure 3 in Niessing et al., 2000). Since N51A already causes a strong phenotype by disturbing hb expression and Cad gradient formation, what is the reasoning being adding extra mutations to this background? Since the mutations in the PEST domain and YIRPYL motif are involved in cad translational repression, it would be more interesting to add them to the R54A mutation and further study the repression of cad? It would also shed light on the unexpected no difference or even decrease in diffusion in the cytoplasm of the R54A mutant which should increase if indeed the cad mRNA binding is being repressed. Have the authors confirmed that their BcdR54A indeed inhibits cad translation?
How many embryos of BcdMM were analysed? The authors should also provide a table with all the values in SI as they have done for all the other reporters.
The claims with eGFP::NLSBcdHD need to be supported by data from multiple embryos. Even if multiple ACF curves are obtained from one embryo, analysing only one embryo is not sufficient. This would clarify the fact that this reporter seems to be able to reproduce the mobility of Bcd in the nucleus.
According to the methods, all reporters were expressed in a bcd null background, made with the bcd1 allele. This allele is also known as bcd085 and according to Driever and Nusslein- Volhard, 1988 (PMID: 3383244), this allele only causes an intermediate phenotype. This indicates that a truncated version of the protein probably still exists on the embryo. Do the conclusions obtained here still hold if a truncated version of the Bcd protein exists in addition to their reporters?

Minor comments:

In line 45: "Morphogens are signalling molecules", the authors should consider removing the word "signalling" since not all morphogens are, especially the one being studied, Bicoid.
In lines 80-81 (and also throughout the text): "We measure the Bcd dynamics at multiple locations along the embryo AP-axis", should be more accurate and changed to anterior and posterior of the embryo. Using "multiple locations along the AP axis" is ambiguous and not exact for what was done.
Throughout the article, the authors refer multiple times to "modes for/of Bcd transport". Since they or others have not proven that Bcd is being transported, which would involve at least another factor, the authors should replace transport by movement, diffusion or a similar word with which they are comfortable.
Suggestion: The authors claim that the Bcd gradient is exponential up to 60% of embryo length. Would this information allow a more precise calculation of the gradient decay length in the exponential region than the 80-100μm stated on line 202?
In lines 258-259, the sentence "Further, Bcd binds to caudal mRNA, repressing its expression in the cytoplasm" should be improved to clarify the role of Bcd in caudal mRNA translation repression and references should be added. This should also be corrected in the following paragraph.
In line 262, "mutations" should be singular since it corresponds to only one amino acid mutation.
Figure 4J needs to be corrected as the fractions of the slow and fast populations do not correspond to what is shown in Table 3. For example, Fslow fraction of AC is ~45% in the figure while it is 36% in Table 3. The problem occurs in all fractions.
In the discussion, in lines 379-380, "Given the changing fractions of the fast and slow populations in space, the interactions between the populations are likely non-linear". What is the reasoning for non-linearity and not interchangeability?
In line 432 caudal should be italicized.
In the discussion, the authors conclude that "In the nucleus, the two populations can be largely (though not completely) explained by Bcd binding to DNA". The discussion would win by explaining all the possible options.

Significance

The results presented in this article advance the knowledge of the field by adding data and quantifications of Bcd mobility at four locations: anterior nucleus, posterior nucleus, anterior cytoplasm and posterior cytoplasm. Until now, FCS studies have focused mostly on measuring the dynamics of Bcd in nuclei at the anterior (Abu-Arish et al. 2010; Porcher et al. 2010) of the embryo. The results are also consistent with what was previously found for eGFP:Bcd in the anterior nucleus. Still, this is not surprising as they use the same reporter as the previous

© 2024. Published by The Company of Biologists under the terms of the Creative Commons Attribution License (https://creativecommons.org/licenses/by/4.0/).

studies.

This article will be interesting to an audience comprising biologists and biophysicists interested in protein diffusion.

As a biologist, I do not have sufficient expertise to completely evaluate if the modelling is performed flawlessly. However, in my understanding, the FCS analysis is crucial for the results and their conclusions, hence the comment on the certainty of the existence of only two Bcd populations, though these populations being previously described with FCS. Comments from a physicist with experience in analysing FCS data are thus necessary.

Author response to reviewers' comments

1. General Statements

We received four reviews for our manuscript. All reviews are largely positive and highlight, as Reviewer 2 states, “The paper will be of interest to a broad audience in developmental biology, molecular biology, and biophysics”.

There are three important points raised across multiple reviews:

- While all reviewers were positive about the FCS, it is a method very sensitive to noise/error. Therefore, they have requested additional controls.
- The link between the theoretical model and the observed dynamics needs to be further clarified. We will add to the theoretical description to explore more clearly the predicted behaviour in the posterior region. We will also improve the Discussion to make sure that the results are better presented.
- While the reviewers do not request us to dissect the specific mechanisms regulating the dynamics (which would be a major new undertaking), they suggest improving the Discussion to make clear what can – and what cannot – be inferred from our measurements regarding the mechanisms underlying Bicoid transport.

To deal with the first point, we will perform additional experiments in the Fritzsche laboratory at Oxford University. We have a first session booked for early July and we are confident we can do these within three months.

To address the other main points, we will carefully rewrite the corresponding sections of the manuscript to ensure the ideas are clearer and to avoid confusion regarding model interpretation.

2. Description of the planned revisions

We thank the four reviewers for their generally positive feedback on the manuscript. Below, we provide a point-by-point response to each reviewer. Our comments are in blue. We have already carried out the minor revisions, such as correcting mislabeling and providing further Method details.

We are performing new FCS and gradient measurements as suggested by the reviewers. We are confident we can have these completed within three months (accounting for the summer break).

Reviewer #1 (Evidence, reproducibility and clarity (Required)):

This manuscript reports a very thorough and careful study of the mobility of Bicoid in the early embryo, explored with single-point fluorescence correlation spectroscopy. Although previous groups have looked into this question in the past, the work presented here is novel and interesting because of the different Bicoid mutants and constructs the authors have examined, in particular with the goal of understanding the role of the protein DNA-binding homeodomain. The authors convincingly show that there is a significant increase in Bicoid dynamics from the anterior to the posterior region

© 2024. Published by The Company of Biologists under the terms of the Creative Commons Attribution License (https://creativecommons.org/licenses/by/4.0/).

of the embryo, and that the homeodomain plays an important role in regulating the protein's dynamics. Their experiments are very well designed and carefully analyzed. The authors also modelled gradient formation to see whether this change in dynamics might play a role in setting the shape of the gradient. I am not sure I fully agree with their conclusion that it does, as mentioned in my comment below. However, it is an interesting discussion to have, and I think this paper makes a significant advance in our understanding of Bicoid's behavior in the early embryo.

We thank the Reviewer for their positive comments and their suggestions for improving the manuscript. We will resolve the concerns raised by the reviewer with clarity in the revision. We will also add additional comment in the Discussion regarding the interpretation of our results.

Major comments:

1) Gradient profile quantification: Some of the conclusions made by the authors rely on the comparison between their model of gradient formation (as captured in the equations in lines 232 and 233) and the Bcd intensity profile measured in the embryos. Since the differences in gradient shape predicted by the different models are very small (see Fig. 3B, which is on a log scale and therefore emphasize small differences, and Fig. 3C), it is very important to understand how reliable the experimental concentration profiles are.

This is a fair comment. It is worth noting that the key differences between the 1- and 2- component models are only apparent at large distances (and hence low concentrations) from the source.

We performed the quantification of the gradients in a manner similar to the Gregor lab, whereby the midsagittal plane is analysed. We used 488nm illumination (rather than 2-photon, as the Gregor lab does) so our measurements are likely noisier. However, we are not investigating the variability in the gradient here, but the mean extent. We currently correct background with a uniform subtraction, but we appreciate that is not the optimal method.

In the revised manuscript, we will repeat the above experiments using a 2-photon microscope. Further, we will image lines expressing His::mcherry without eGFP under the same imaging conditions to more accurately estimate the background signal. While we expect this to improve the data quality, we do not envisage significant change to the observed profiles based on prior experience.

At the moment, I do not find the evidence that [Bcd] concentration profile is more consistent with a 2-component diffusion model than a 1-component model very strong. A few comments related to this:
1a. Line 249, it is mentioned that: "observations ... incompatible with the SDD model". Which observations exactly are incompatible with the SDD model?
The key points are in the preceding paragraph. We will improve the model presentation in the Results and also include further contextualisation in the Discussion.

1b. In Fig. 3D, only the prediction of the 2-component model is shown. What would the simple 1-component diffusion model look like? Is it really incompatible with the data?
We agree with this comment and will provide the 1-component fit to the gradient profiles. We expect it to fit well for the anterior half of the embryo but fail at larger distances (as has been previously shown).

Regarding the FCS data, we also show one and two component fits. We will show the alternative fits - a 2 particle fit is clearly an improvement (see also related response to reviewer 2).

1c. Line 243: "The increased fraction in the fast form ... consistent with experimental observation of Bcd in the most posterior" (Mir et al.)". I am not sure how this is significant, since the simple model also predicts there will be Bcd in the posterior - the only difference is how much is there (as shown in Fig. 3C), and it's a very small difference.
The absolute differences are not large between the two models, but due to the observed clustering (Mir et al. 2018), even small differences can have very large effects. In the revision we will provide estimates of the actual concentration differences.

© 2024. Published by The Company of Biologists under the terms of the Creative Commons Attribution License (https://creativecommons.org/licenses/by/4.0/).

We are performing new experiments with the Fritzsche lab at Oxford to estimate if there is clustering of Bcd. We will also repeat our FCS experiments to validate our key conclusion of AP differences in diffusion of Bcd. These should be completed by the end of the summer.

1d. Since the difference between models is in the posterior region where Bcd concentration is very low, when comparing the models to the data the question of background subtraction is essential. How was the subtracted background (mentioned line 612) estimated?
See above response to the first comment.

1e. Along the same line, were the detectors on the Zeiss LSM analog or photon counting detectors, and how confident can we be that signal is exactly proportional to concentration?
We used PMTs and did not directly do photon counting. But the intensity is still proportional to the concentration. It is possible to estimate the absolute concentration value, *e.g.*, Zhang et al., 2021 (https://doi.org/10.1016/j.bpj.2021.06.035). However, our main conclusions - especially regarding the spatially varying Bcd dynamics - are not dependent on this.

1f. Can the gradients created by the two Bcd mutants (FIg. 4B) be quantified as well, and are they any different from the original Bcd gradient?
We agree this would be useful. We will provide the gradient quantifications of the *bcd* mutants in the revision.

1e. What is the pink line in Figure 5C (I am assuming the green one is the same as in Fig. 3D)? It could be better to not use normalization here, or normalize everything respective to the eGFP::Bcd data to make comparison in relative concentrations in the posterior for different constructs more evident (also maybe different colors for the three different data sets would help clarity).
This is a fair comment, and we will create graphs with new data for better visualisation.

1f. Discussion, lines 402-403: Does the detailed shape of the Bcd in the posterior region matter at all, since the posterior is not a region where Bicoid is active, as far as we know? Could a varying Bcd dynamics have other consequences that would be more biologically relevant?
Bcd is now known to act at 70% EL (Singh *et al.*, Cell Reports 2022). So, the gradient is relevant for a large extent of the embryo length, though it is not known if there is any effect in the most posterior region.

2) Model for gradient formation (lines 231-238):
2a. Whether the molecules of Bcd can change from their fast to slow form is never questioned. How do we know (or why might we suspect) they do exchange?
This is a good point. Within the nucleus, and based on our mutant data, we suspect the fast/slow forms correspond to unbound/bound DNA states.

In the cytoplasm, the dynamics are less clear. Bcd can bind to cytoskeletal elements (Cai *et al.*, PLoS One 2017) as well as to Caudal mRNA. Therefore, it seems reasonable to have different effective dynamic modes - yet, how such switching occurs remains unclear.

Ultimately, our model approximates multiple dynamic modes that are integrated to drive Bcd motion. Including switching between states is a reasonable assumption based on what is known about cytoskeletal and protein dynamics, but we do not have a specific mechanism.

It is challenging to estimate a specific k_{on} / k_{off} rate, as the dynamic changes also depend on the diffusion - which itself is changing. For now, we believe our level of abstraction is appropriate given what is known about the system. It will be very interesting to explore the specific interactions underlying such behaviour in the future, but that is beyond this current manuscript.

2b. The values used in the model for alpha, beta_0 and rho_0 should be mentioned. Maybe having a table with all the parameters in the method section, or even in the supplementary section, would help. The exact values of alpha and beta matter, because if they are large (fast exchange) a single exponential gradient is to be expected, if they are 0 (no exchange) a double exponential gradient is to be expected, with intermediate behavior in between. Which case are we in here?
We agree and will add a more complete table in the revision.

© 2024. Published by The Company of Biologists under the terms of the Creative Commons Attribution License (https://creativecommons.org/licenses/by/4.0/).

3) Discussion about anomalous diffusion (lines 386-388): The 2-component model used by the authors to interpret their FCS data seems very well justified here (excellent fits with very small residuals). I agree with the authors' conclusion that "the dynamics of Bcd within the nucleus are more complicated than a simple model of bound versus unbound Bcd", but I don't see how that can lead to a diagnostic of anomalous diffusion instead. Maybe it is just a matter of exactly explaining what is meant by anomalous diffusion here (since this term is often used to mean different things). A more likely scenario I think, is that there are more than just two Bcd components in the system.
This is a good point, and we can't easily differentiate two/multi- component fits from anomalous diffusion ones. This is a known problem. But we have recently shown in a collaboration with the Laurent Heliot lab (Furlan et al, Biophys J 2019), that anomalous diffusion is a good stable indicator of changes, even if it might not be the right model. We use anomalous diffusion as it stably predicts changes. We do not claim, however, that diffusion is anomalous. We will improve the discussion of these points in the revised manuscript.

4) Line 440 and after: What is the evidence that the transition between the two forms might vary non-linearly with Bcd concentration? How would that help adapt to different embryo sizes? It would be good to be more explicit here instead of just referring to another paper.
We will improve this discussion. The central point is that the action of Bicoid is unlikely to simply depend linearly on concentration as in that case the ratio of fast to slow forms would be constant across the embryo. Related to the above comment, it is important to emphasise that we are using a phenomenological model, not one based on a specific mechanism.

5) Since an important aspect of this work is the study of different Bcd constructs *in vivo*, it is important that these constructs are very clearly described, so the section on the generation of the fly lines (Methods) should be expanded. In particular:
5a. It seems that the eGFP:: NLS control used here was different from that first described in Ref. 64 (and used for FCS experiments in Ref. 30 and 36)? If so, what NLS sequence was used here, and precisely what type of eGFP was used (in particular, was the A206K mutation that prevents dimerization present in the eGFP used)? If it is the same construct as in Ref. 64, it should be mentioned explicitly.
5b. Were the mutant N51A and R54A lines gifts as well, or have they been described before? If so, previous publications should be referenced. If not, how the plasmid was introduced in the embryo should be briefly explained.
We agree and will expand on the fly lines in the revision.

6) Concentration calibration measurements (Methods Fig. 2, line 568 and on). It is well known that background noise is going to interfere with the measurement of N when the signal becomes equivalent to the background noise (Koppel 197, Phys Rev A 10:1938-1945, and for a recent discussion of this effect for morphogens in fly embryos: Zhang et al., 2021, Biophysical Journal 120,4230-4241). It is almost certain that in the low signal regions of the embryo (e.g. posterior cytoplasm) this is affecting the reported concentration, and should be at least acknowledged.
We agree with the reviewer. We will provide the SBR. We will also correct the N values based on the method followed in Zhang et al., 2021, Biophysical Journal 120,4230-4241.

7) Reference 3 is mis-characterized in two different ways in the manuscript:
7a. Line 50: The conclusion in Ref. 3 was not that the gradient was due to a diffusive process, on the contrary Gregor et al. argued that Bcd was too slow to form such a long-range gradient by diffusion. Studies that do present data consistent with a morphogen gradient formation mechanism driven by diffusion are reference 5, reference 30, Zhou et al., Curr. Biol. 2012;22(8):668-75 and Müller et al., Science 336 (2012) 721-724.
Gregor *et al.*, do not argue against a diffusion process - indeed, they utilise a SDD model in their paper. However, they do extensively discuss how the predicted dynamics from the SDD model are not compatible with gradient formation as observed after n.c. 13. This problem was resolved to some degree by FCS measurements of Bcd (e.g., Dostatni lab, Development 2011) and the use of a Bcd tandem reporter which showed that production and degradation change during n.c. 14 (Durrieu *et al.*, MSB 2018). We will improve the framing of these results in the revision.

7b. The diffusion coefficient estimated from FRAP measurements and reported in Ref. 3 (D = 0.4 micron^2/s) is mentioned a couple of times in the manuscript (line 66, line 395, line 411). However, this number is simply incorrect. When fast components (such as the ones clearly detected

© 2024. Published by The Company of Biologists under the terms of the Creative Commons Attribution License (https://creativecommons.org/licenses/by/4.0/).

here by FCS) are present, they diffuse out of the photobleached area during the photobleaching step. If that is not corrected for during the analysis (and it wasn't in Ref. 3), then the recovery time measured is just equal to the photobleaching time, and has nothing to do with either the fast or slow fraction of the studied molecule - it has no other meaning than to give a lower bound on the value of the actual effective diffusion coefficient of the molecule. This effect (called the halo effect) is well known in the FRAP community (see e.g. Weiss 2004, Traffic 5:662-671), it has been experimental demonstrated to occur for Bcd-eGFP in the conditions used in Ref. 3 (Reference 30), and the actual diffusion coefficient that should have been extracted from the data presented in Ref. 3 has been recalculated by another group to be instead D = 0.9 micron^2/s (Castle et al., 2011, Cell. Mol. Bioeng. 4:116-121). It would therefore be better to report the corrected value from Castle et al. to help the field converge towards an accurate description of Bcd mobility.
We fully agree and will use the improved FRAP estimated value for Bcd.

Minor comments and suggestions:

8) Figure 1: From panel A, it seems that what is called "Anterior" and "Posterior" is about 150 micron away from the embryo mid-section, i.e. about 100 micron from either the anterior pole or the posterior pole (so not the tip of the embryo, but somewhere in the anterior half or posterior half). Maybe this should be made clear in the text.
We have made changes in Figure 1A to indicate the region within which the FCS measurements are carried out. We have added the relevant details in the legend of figure 1 lines 137-138.

9) Fig. 2A; It might be good to put this graph on a log scale, so that cytoplasmic values are seen more clearly. Also, what about reporting on nuclear to cytoplasmic ratios?
We will rework on this graph and make necessary changes.

10) Fig. 2: It could be interesting to plot D_effective as a function of the measured concentration of Bicoid in different locations, since the (interesting) suggestion is made several time that [Bcd] could the a determinant of the protein mobility.
Our work provides an indication that Bcd concentration is connected to the diffusion. We did this by measuring at two locations. To extend this to a rigorous model would require substantial new measurement along the whole length of the embryo. While interesting, this represents a very large investment of time and lies beyond the current manuscript.

11) Figure 3B&C: Is the curve for 2-component diffusion (without concentration dependence) for steady-state missing?
We will clarify in the revision.

12) Lines 78 and 471: What do the authors mean by "new reagents"? The word reagent evokes a chemical reaction, but there are none here. Do the authors mean new constructs? or new mutants?
We have changed lines 78 and 479 from “new reagents” to new Bcd mutant eGFP lines”.

13) Lines 57-59: Another good reference for FCS measurements performed to study the dynamics of a morphogen (in this case Dpp) is Zhou et al., Curr. Biol. 2012;22(8):668-75
We added this reference in no.70.

14) Lines 109-111: A word must be missing. Precisely determined what?
Precisely measure within cytoplasm, and nuclear compartments and also during interphase stages. We have changed to “precisely measure in the cytoplasmic and nuclear regions during the interphase stages of nuclear cycles (n.c.)12-14.” in line no.111-112.

15) Line 278: The increase in the slow mode is expected. Maybe explicitly mention why.
In line 286, we have added “due to the loss of Bcd binding to the DNA”.

16) Line 282: "with the fast component increasing", maybe replace with "with the diffusion coefficient of the fast component increasing" or "with the fraction of the fast component increasing".
We have changed line 289 “with the diffusion component of fast component increasing towards the posterior”.

© 2024. Published by The Company of Biologists under the terms of the Creative Commons Attribution License (https://creativecommons.org/licenses/by/4.0/).

17) Line 517: Is there a reason why the dorsal surface is always placed in the coverslip?
We have added these details in line 528-529 in Methods.

18) Line 524 and on: FCS measurements: What was the duration of each individual FCS measurement? It is great that the exact number of measurements are reported in the supplementary!
Thank you for the complement. Typically, cytoplasmic measurements are 60secs and nuclear measurements are 20-40s. We have added this in line no.528-529. We also added a column to indicate the duration of each of the measurements in the supplementary tables.

19) An Airy unit of 120 um seems large in combination with an objective with a NA of 1.2, is there a reason for that? What was the radius of the resulting detection volume?
Olympus microscopes have a 3x magnification stage in their confocals. This leads to the change in the Airy unit. Otherwise, it would be 40 mm.

20) Thank you for detailing the reasons behind the choice of excitation power, an important and often omitted details. Where in the excitation path were the values of the laser power measured (before or after the objective?)?
Thank you for the complement. The laser power is measured before the objective. We removed the objective and measured the laser power in the objective path.

21) Line 585: "since the brightness of eGFP::Bcd..." do the authors mean the molecular brightness of a single eGFP::Bcd molecule, or the total fluorescence signal?
It is the total fluorescence signal. We have edited line no.592.

22) It would be good for reference to mention the approximate value of the molecular brightness recorded for these eGFP constructs at the laser power used.
We will measure and tabulate in the revised manuscript.

23) Reference 766: The year (and maybe other things) is missing. We have corrected this reference.

24) Figure 2 (Methods): The concentrations shown on the figure should be in nM not uM.
Thanks for noticing – we have changed.

Reviewer #1 (Significance (Required)):

Strengths:
Very careful and systematic study of Bcd's dynamics in the early embryo
Use of several mutant and truncated forms of Bcd to pinpoint the importance of the DNA binding domain in setting this dynamics
Uncovers a previously unknown change in Bcd dynamics from the anterior to the posterior of the embryo
Modelling of the Bcd concentration gradient shape taking into account the measured dynamics

Limitations:
The quantitative comparison between modelled and measured gradient could be improved. The discussion of the biological implications of the work is limited

Advance:
Uncovers a previously unknown change in Bcd dynamics from the anterior to the posterior of the embryo. This raises very interesting questions about molecular mechanisms involving Bicoid.
Other studies (cited in this manuscript) reported on Bcd dynamics, but the present study represents a very welcome expansion of these earlier studies, by looking at spatial dependence and by examining several Bcd constructs.

Audience:
- Somewhat specialized, as this work should firstly be of interest to scientists studying morphogen gradients. However, it is also a beautiful example dynamical studies in vivo, so it will also be of interest to experimental physicists studying protein motions in vivo (a rather large community).

© 2024. Published by The Company of Biologists under the terms of the Creative Commons Attribution License (https://creativecommons.org/licenses/by/4.0/).

We thank the Reviewer for the critical but positive comments on the manuscript. We believe that making the above changes will lead to a more robust manuscript.

Reviewer #2 (Evidence, reproducibility and clarity (Required)):

SUMMARY

In this study, Athilingam et al. investigated Bicoid (Bcd) protein dynamics along the anterior-posterior axis of Drosophila embryos. They performed Fluorescence Correlation Spectroscopy (FCS) experiments to analyze Bcd protein diffusion and found that Bcd had a 1.7-fold higher mobility in the posterior compared to the anterior. The authors also generated Bcd homeodomain mutations and analyzed their impact on gradient formation. They found that the BcdN51A mutation exhibited altered nuclear dynamics with faster diffusion, while cytoplasmic dynamics remained unchanged. BcdR54A embryos showed dynamics more similar to the wild-type Bcd, with a minor decrease in slow diffusion in the posterior region. Interestingly, the Bcd homeodomain alone, when fused to eGFP-NLS, was able to replicate the observed Bcd protein dynamics, particularly in the slower diffusive mode. The paper provides evidence that the Bcd homeodomain has a significant influence on protein dynamics and suggests a complex interplay of interactions between Bcd, nuclear DNA, and cytoplasmic RNA that regulate Bcd diffusion and function.

Overall, the data and methods presented in the paper are clearly described. The experiments are adequately replicated, and the statistical analysis appears sufficient. Prior studies are referenced appropriately, and the claims and conclusions are mostly supported by the data. However, the following points should be addressed to corroborate the conclusions:

We thank the reviewer for the appreciation of our work. Below, we address the specific issues raised.

MAJOR POINTS

1) FCS measurements and fits
a) Please state the duration of each individual FCS measurement.
In the cytoplasm, the measurements were carried out for 60 secs and in nuclei it is between 20-40s. We could not measure for 60s in the nuclei as the nuclear position fluctuates from its initial position. We will add another column to indicate the duration of FCS measurements in the supplementary tables.

b) The authors acknowledge potential issues with fluorophore photophysics and use different lag time ranges for the calibration dye Atto-488 (0.001 ms in Method Fig. 2) and eGFP (0.1 ms in the main figures). Given the strong influence of different parameters on data interpretation and conclusions, Method Fig. 2 should be repeated with purified eGFP. This is particularly relevant for the noisy FCS measurements in posterior regions.
Performing the experiment with purified eGFP will be a volume calibration. We routinely performed this before each imaging session, and that should be fluorophore independent. As noted by Reviewer 1, it is also important to be clear about background correction. We will provide brightness data for eGFP and background values in the revised manuscript. We can then use this to estimate the corrected concentrations.

We use 0.1 ms to start, as at that point any contribution from the photo-physics should have decayed (0.1 ms is about 3-5 times the day rate of the photophysical process, Sun *et al.*, Analytical Chem 2015).

c) Please explain why no data is shown for "AN" around 0.1 ms lag time in Fig. 1B in contrast to all other figures.
We will add the data for AN from 0.01 in the revised figures.

d) Please state what the estimated diffusion coefficients with one-component model fits are. Please also explain why the fits in Fig. S1E do not reach a value of 1 and why they plateau higher than the experimental data at long lag times. Please constrain the fits to G=1 at 0.1 ms tau and G=0 at 1 s tau to make a fair comparison.

© 2024. Published by The Company of Biologists under the terms of the Creative Commons Attribution License (https://creativecommons.org/licenses/by/4.0/).

The experimental ACF curves reach 0 at long lag times as would be expected. The one- component fits, however, don't describe the data well and as a result they do not reach 1 and 0 at short and long lag times, respectively. The fitting is done using a mean-squared estimation of the best approximation of the particular model function to the data. Fixing the parameters can be done, but it will further reduce fit accuracy and deviations will be larger. We will perform this analysis and tabulate the one component fits in supplementary 1 with necessary corrections.

e) Please assess the validity of all multi-component fits by comparing the relative quality of the models to the number of estimated parameters using the Akaike information criterion or similar approaches.
We will provide the values denoting the quality of the fits in the revision. We will provide the 3D 1 particle fit, the 3D 1 particle fit with triplet, the 3D 2 particle fit and the 3D 2 particle fit with triple and will provide appropriate measures of fit quality.

f) Please also present the Bcd-GFP fits with 0.001 ms that are mentioned in line 590, and present the results for the data that did not give comparable tau_D1 and tau_D2 values mentioned in line 593.
We will provide all the curves from 0.001ms in the supplementary. We did not provide these details as we have followed the methods from Abu Arish *et al*., 2010. As our cytoplasmic and nuclear TauD values match with Abu Arish et al., 2010 and Porcher et al., 2010, we thought the excess data would be redundant.

3) Bicoid gradient and modeling
a) Little et al. 2011 observed that the Bcd gradient decreases around n.c. 13. Can the authors of the present work observe a similar concentration decrease using FCS? This is important to i) validate the FCS concentration measurements, and ii) to resolve the controversy regarding "previous claims based on imaging the Bcd profile within nuclei, which predicted decrease in Bcd diffusion in later stages".
This is a good point regarding conclusions from the previous literature. The Little *et al*. paper inferred that diffusion had to decrease from fitting to the gradient profiles. However, subsequent analysis from our lab (Durrieu *et al.*, MSB 2018 [which uses a different method involving a tandem reporter for Bicoid] and this manuscript) strongly suggest that Bicoid remains dynamic, at least through n.c. 13 and early n.c. 14. One way to test this is to use SPIM-FCS, where longer time courses can be taken (though with slower time resolution in the FCS). We have performed preliminary experiments with SPIM-FCS and we will revisit this data to see if we can find evidence for changes in the diffusion.

We will also extend the Discussion to make the results clearer in terms of previous models and literature.

b) Please explain why the experimental Bcd-GFP gradient data does not reach a value of 1 (e.g. in Fig. 3D) despite normalization. Please also explain why the fits become flatter in Fig. 5B compared to the steep fit in Fig. 3D.
Both lines were measured under identical conditions. Therefore, we normalised to the maximum value of both experiments. We will redo, normalising to each individual experiment. Regarding Fig. 5C, the Bcd::eGFP curve is identical to Fig. 3D. The flatter curve is the line with eGFP tagged to a NLS alone.

c) For modeling, please take into account observations that the Bcd source is graded with a wide distribution (30-40% EL, see Spirov et al. 2009, Little et al. 2011, Cai et al. 2017 etc.). The extent of the source used in the present work (x_s=20 um, line 620) is at least five times too small.
Care must be taken in defining the source extent. The most careful measurements are reported in Little *et al.,* PLoS Biology 2011 who performed single molecule FISH. They conclude "We demonstrate that all but a few mRNA particles are confined to the anterior 20% of the egg". Further, the peak in the particle density is around 20-30mm from the anterior (Figure 3, Little *et al.,* PLoS Biology 2011), with the vast majority of counts being with 10% of the anterior pole. Further, Durrieu *et al.* MSB 2018, showed using a Bcd tandem reporter that there was unlikely to be an extended gradient of *bcd* mRNA (maximum extent of around 50mm). Here, we used a simple source domain, which was arguable a little narrow, but not significantly so. We will increase the value in the revision, but the claim that there is an extended *bcd* mRNA gradient (Spirov *et al.,*

© 2024. Published by The Company of Biologists under the terms of the Creative Commons Attribution License (https://creativecommons.org/licenses/by/4.0/).

Development 2009) has not been substantiated by later experiments.

d) Please discuss in the paper how well the simulations in Fig. 3B agree with the experimental data.
We will provide these details in the revision.

e) Please provide a precise estimate for the statement "Even with an effective diffusion coefficient of 7 μm2s-1, few molecules would be expected at the posterior given the estimated Bcd lifetime (30-50 minutes)" to turn this into a quantitative argument. How many molecules are expected to reach posterior in which model, and how does it compare to experimental observations?
This can be estimated based on the root-mean-square distance for diffusive processes. We will provide this in the revision.

f) The sentence "we find that a model of Bcd dynamics that explicitly incorporates fast and slow forms of Bcd (rather than a single "effective" dynamic mode) is consistent with a range of observations that are otherwise incompatible with the standard SDD model" needs to be toned down and corrected since a simple SDD appears to be sufficient to account for the observed gradients. If the authors disagree, please specifically point out in the paragraph around line 249 what observations exactly are incompatible with a standard SDD model.
This is similar to the point raised by Reviewer 1. While the standard SDD model can explain the overall gradient shape, it is not compatible with the observed time scales and Bcd puncta tracked in the posterior pole. We will improve the Discussion around this point to make the distinctions between the models clearer.

5) Data presentation
a) In line 27 and 122 it would be better to rephrase the wording "find/found" and give credit to previous papers that first made these observations.
We will edit in the revision.

b) For the statement "This suggests that the dynamics of the fast fraction were not captured by previous FRAP measurements", please explain why this should not be the case even though the fast fraction is shown to be larger than the slow fraction in the current work.
We will edit in the revision.

c) Similarly, the sentence "The dynamics of the slower mode correspond closely to measured Bcd dynamics from FRAP" likely needs to be corrected since it neglects the contribution of the faster mode, which is fluorescent as well and should also contribute to the dynamics from FRAP.
This is similar to the point raised by Reviewer 1 and we will edit in the revision.

d) In the absence of further evidence (see above), the sentences "We establish that such spatially varying differences in the Bcd dynamics are sufficient to explain how Bcd can have a steep exponential gradient in the anterior half of the embryo and yet still have an observable fraction of Bcd near the posterior pole" and "These results explain how a long- ranged gradient can form while retaining a steep profile through much of its range" in the abstract need to be toned down.
We are not sure here what needs to be toned down. Our results show that there are (at least) two dynamic forms of Bcd and, combined, they are capable of forming a long-ranged gradient while also ensuring the gradient remains steep in the anterior (because the diffusion coefficient itself varies across the embryo). We will go through these statements and make sure the meaning is clear.

e) The authors state that "However, we show that eGFP::Bcd in its fastest form can move quickly (~18 μm2s-1), and the fraction of eGFP::Bcd in this form increases at lower concentrations", but this has not been directly shown. Please tone down this statement or directly test the prediction that Bcd has a higher fraction of the fast form in earlier nuclear cycles when Bcd concentration is smaller.
This is a good suggestion, and we will test whether early nuclear cycles of the anterior domain show faster dynamics.

MINOR POINTS
1) Introduction

© 2024. Published by The Company of Biologists under the terms of the Creative Commons Attribution License (https://creativecommons.org/licenses/by/4.0/).

a) Please explain explicitly what exactly the contention in Bcd, Nodal and Wingless dynamics is in the cited references.
We will add in the revision.
b) In line 95, it would be better to state that this is a variation of the SDD model rather than "a new model".
We changed from “a new model” to “an improved version of SDD model” in the current version of the manuscript.
2) Methods
a) The authors state that "The same software was also used to calculate the cross-correlation function", but I couldn't find any cross-correlation analyses. Please clarify.
It is line 538. There is no cross correlation. We changed this to the autocorrelation function.

b) Please correct the "uM" typo to "nM" in the legend of Method Fig. 2A.
We have changed this in the current version.

c) In the sentence "Further, since the brightness eGFP:Bcd in the anterior and posterior cytoplasm is lower compared to the nuclei", "brightness" probably needs to be changed to "concentration" since the molecular brightness is unlikely to change.
We edited the line no.591.

d) Please explain the background-correction method mentioned in line 612. Please also state at what temperature the experiments were performed.
We will add a better background correction in the revision. Currently, it is the non-embryo background as background noise. The measurements are carried out at 25°C.

3) Results
a) Please provide labels for anterior, posterior, dorsal and ventral in Fig. 1A.
b) Please explain the colors in Fig. 5C.
c) Please explain the dashed lines in Fig. 3C.
We have edited Figure 1A and Figure 5C. We will edit Figure 3C in further revision.

OPTIONAL
1) If possible, it would be helpful to mention whether the transgenic animals have any abnormal phenotypes or whether they can rescue the bcd mutant.
We will update in the revision.

2) To validate the concentration measurements, it would be ideal if the authors could determine the Bcd concentration gradient using FCS along the anterior-posterior axis. This would also address whether there are further unexpected changes in diffusivity in medial regions and along the anterior-posterior axis that would have to be considered for modeling.
To measure the Bcd concentration using FCS along the whole axis would be a very challenging undertaking. To get the data for the two positions analysed already represents a significant amount of work. We have done SPIM-FCS measurements, and we will be repeating our FCS measurements in the Fritzsche lab at Oxford. Combined, we believe this provides sufficient corroboration of our results.

3) Local photoconversion experiments, e.g. in Bcd-Dendra2 embryos if available, would provide compelling support for the relevance of the measurements in the current work.
This is a nice idea, but this would represent a substantial project in its own right and lies beyond the current work.

Reviewer #2 (Significance (Required)):

The paper investigates the diffusion dynamics of Bicoid (Bcd), a transcription factor crucial for establishing the anterior-posterior axis during Drosophila embryogenesis. The authors utilize Fluorescence Correlation Spectroscopy (FCS) and various Bcd mutants fused with eGFP to understand the role of Bcd's homeodomain and other domains in its nuclear and cytoplasmic diffusion dynamics. They performed elegant experiments using insightful transgenic lines, showcasing well-designed and well-executed methodology. The paper is very nice to read, with a clear and engaging writing style and excellent presentation of the data, making it easy to follow

© 2024. Published by The Company of Biologists under the terms of the Creative Commons Attribution License (https://creativecommons.org/licenses/by/4.0/).

and understand their findings.
We thank the Reviewer for the overall positive view on the manuscript.

However, there are a few limitations to consider. First, the paper does not provide evidence for the switching between slow and fast populations central for their modeling, leaving an important aspect of the dynamics unexplained. Second, there are doubts regarding the accuracy of the model used to fit the FCS data (see detailed comments in the section "Evidence, reproducibility and clarity"), underscored by the statement "While the increase in the slow mode was expected, the reason for the change in the fast mode is less clear". Third, the relevance of a potentially higher Bcd mobility for gradient formation remains unclear. For example, the fit in Fig. 3D deviates substantially from the data around 0 um embryo length, which is likely even larger than the error expected from a "simple diffusion" fit at the posterior end of the embryo. In addition, in Fig. 3C the lines for the different models appear to be indistinguishable given the noisy measurements, calling the relevance of the findings into question.
This concern is similar to that raised by Reviewer 1 regarding the accuracy of the model. We emphasise that the model is a phenomenological approach to contextualise our results, rather than meant to provide a mechanistic understanding of the dynamics. We will clarify this in the revision to hopefully make clear these distinctions.

Overall, the paper extends our knowledge by providing new insights into the role of the Bcd homeodomain in determining Bcd gradient formation. The paper highlights that homeodomain interactions in the cytoplasm and nuclei are significant contributors to determining Bcd dynamics. Additionally, the paper suggests that additional components within Bcd itself or other proteins in the cytoplasm affect Bcd dynamics at different Bcd concentrations. The paper will be of interest to a broad audience in developmental biology, molecular biology, and biophysics.
Researchers studying transcription factor dynamics, morphogen gradients, and Drosophila embryogenesis will find this study particularly valuable. While the study is primarily focused on basic research, the insights gained on Bcd diffusion dynamics and the role of the homeodomain may contribute to a broader understanding of transcription factor regulation and function in other systems.
We thank the Reviewer for the critical but overall positive review. We believe we can address all the major substantive points prior to resubmission.

Reviewer #3 (Evidence, reproducibility and clarity (Required)):

The manuscript presents experiments that combine a number of techniques to produce an extremely detailed view of Bcd gradient formation in the early Drosophila embryo. The authors provide both empirical and theoretical evidence that support a conclusion that Bcd has both slow and fast moving forms, that the proportion of fast vs slow Bcd is higher at the posterior of the embryo, and that a theoretical model involving both fast and slow moving components fits empirical observations substantially better than a simple diffusion model. Next, they extend the work by investigating what functional motifs of Bcd are required for gradient formation. They demonstrate that a missense mutation N51A in the homeodomain (HD) that affects both DNA binding and cad regulation has major effects on Bcd gradient formation, while the R54A mutation that only affects cad regulation does not. They further show that a protein composed of only an NLS and the Bcd HD can form a gradient that is similar to full-length Bcd, although the two-component dynamics are not recapitulated by the HD-only form.

In my estimation the experimental work is rigorous and the results fully support the conclusions of the authors. I was surprised, however, that the HD-only form localizes via very different and simpler dynamics than does full-length Bcd, but nevertheless forms at least a qualitatively similar gradient. That leads to the question as to whether the existence of the fast and slow forms and their different ratios in different parts of the embryo actually are physiologically relevant. I don't see a straightforward way to test this experimentally, because the mutations that effect Bcd gradient formation also affect essential functions of the protein that if abrogated produce severe downstream effects on embryonic development and lethality. However I would like to see this point at least addressed in the discussion. The data and the methods are presented in such a manner that they can be reproduced, and the number of replicates and statistical analysis is overall robust.
We thank the Reviewer for the positive and constructive review. They, like both previous

© 2024. Published by The Company of Biologists under the terms of the Creative Commons Attribution License (https://creativecommons.org/licenses/by/4.0/).

reviewers, raise the issue of the model and how it fits with the data. As outlined above, we will improve this part of the data presentation and also the Discussion to make sure the main results are clear.

We agree that the underlying importance of the different dynamic forms of Bicoid – and why they change across the embryo – remains unknown. We believe that our careful characterisation of such behaviour is important nonetheless, as it reveals that: (1) morphogen dynamics are more complicated than typically modelled, and this may be just as relevant for ligands moving through extracellular space; and (2) dynamics can vary in space/time, providing an additional possible mechanism of control for regulating morphogen gradient profiles.

Of course, we would like to explore potential physiological relevance. Further exploration of the homeodomain and its role in regulating dynamics is a potential route, but that belongs in future work.

Minor comments:

The presentation of the graphical data measuring Bcd levels along the a-p axis (Fig 1C, 1D, 4C-F and others) needs to be improved, because the grey lines that represent ACF curves are essentially invisible. This is partly because there is usually extensive overlap between the grey lines and other lines. This may be solved by using a more vivid colour than grey for the ACF curves, or perhaps the ACF lines could be made thicker but with some transparency so that overlapping data can be seen. In any event this aspect of the presentation needs to be improved.
We have made the ACF lines thicker to distinguish from the model fit.

In Figs 2D and 2I measurements of statistical significance between the proportion of protein in fast and slow modes need to be added.
We will add in the revision.

Relevant to line 174 and Fig 2, NLS should be defined when first used, the source of the NLS should be given (is it from Bcd?) and the rationale for looking at eGFP::NLS should be made explicit.
We have added details on how the eGFP::NLS is generated in the methods.

In Fig 3D the dashed lines need to be defined. I assume these are experimental error bars but this is not stated.
We now state this in the legends.

On lines 344-5, shouldn't this conclusion concern the HD rather than the NLS?
Yes, thanks for pointing it out it is related to only NLS not NLS^{HD}. We removed this statement from line 351.

On line 432, CAP is not an acronym, the correct term is 5' 'cap' or 'cap structure'. Also Cho et al. PMID 15882623 should be added to the references here.
We changed the corresponding section and added the references.

On lines 446, 456, 469, and throughout: replace 'blastocyst' with 'blastoderm'. The former term is generally used for embryos that undergo full cellular divisions and cleavage in early embryogenesis, not for syncytial embryos such as Drosophila.
We have changed blastocyst to blastoderm throughout the manuscript.

Reviewer #3 (Significance (Required)):

General assessment: The main strength of the paper is that it extends our knowledge about the dynamics of Bcd gradient formation, and by so doing it advances our understanding of the physical parameters underlying morphological gradients and patterning. The authors are admirably open about questions that were unanswered by the study, which include identifying molecular interactions that affect Bcd dynamics in the cytoplasm, whether Bcd diffusion is dependent on its concentration, and whether other morphogens form gradients in a similar manner. Answering any of these questions would involve substantial experimental work and it is appropriate to leave these questions for subsequent manuscripts.

© 2024. Published by The Company of Biologists under the terms of the Creative Commons Attribution License (https://creativecommons.org/licenses/by/4.0/).

Advance: A number of high-profile papers were published on this topic in the late 2000s and early 2010s that reached difficult conclusions, so the nature of the mechanisms underlying Bcd gradient formation have remained controversial and somewhat opaque. While this paper does not provide a definitive answer to all relevant open questions, it nevertheless represents a significant advance toward their resolution.

Audience: This paper will be of interest both to developmental biologists interested in gradients and pattern formation, and to biophysicists interested in physical parameters affect molecular movements. It is fundamental research that does not have an obvious clinical or translational component.
We thank the Reviewer for the constructive comments and also for recognising that a number of our unanswered questions belong in future work.

Reviewer #4 (Evidence, reproducibility and clarity (Required)):

Summary:
Athilingam et al. are interested in understanding how the Bicoid (Bcd) morphogen gradient is formed in the early stages of Drosophila embryonic development. Using fluorescence correlation spectroscopy (FCS), the authors quantify the dynamics of Bcd in nuclei and cytoplasm at anterior and posterior regions of the embryo.
First, they characterize the dynamics of eGFP::Bcd in space and time. Analysing FCS autocorrelation curves at the anterior and posterior regions of the embryo during the interphases of nuclear cycles 12 to 14, they detect differences in Bcd diffusion and are able to infer and distinguish two Bcd populations with slow and fast diffusions. Moreover, these dynamics do not vary between nuclear cycles.
Using the different diffusions of the slow and fast populations, they find a model capable of explaining the formation of the gradient at larger distances and the existence of the Bcd molecules in the posterior, compatible with the SDD model widely accepted in the community. Lastly, given that Bcd has multiple roles binding to DNA and also to RNA through its homeodomain, mutations affecting DNA/RNA and RNA only binding are used. Using a mutant without the ability to bind to DNA, their determine that the slower diffusion in the nuclei are due to DNA binding. They further confirm this by fusing the homeodomain of Bcd to eGFP::NLS.

Major comments:
The averaged autocorrelation curves were fitted to models of diffusion with one and two components. The one-component model was insufficient to reproduce the data and the two-component model seems to fit the data. Have the authors tested models with more than two components? Could it be possible to distinguish more Bcd populations?
While it is possible to fit with further components, it rarely provides useful further insight. In particular, the error in measuring three tau_D's is typically very large. In addition, the improvement in the fit will be marginal, and thus the extra components cannot be justified statistically. Of course, we cannot exclude a third (or more) possible dynamic modes, but within the resolution of our FCS measurements two components with triplets are in general the maximum that can be accommodated without overfitting. We will provide evidence for this claim in the supplement of the revised manuscript.

In Figure 2E, the same concentration of eGFP::NLS is estimated to exist in the cytoplasm and nucleus. Since the NLS should target eGFP to the nucleus, what is the explanation for this observation? Is it possible that the method used to estimate the concentration of molecules is underestimating the concentration in the nucleus or the opposite in the cytoplasm?
This is a good observation. There are two possible explanations. First, the regular division cycles “reset” the nuclear levels. Therefore, differences may not be so large. Second, FCS measurements of concentration can be noisy, as they depend on the very short time scales in the measurement. We will double check our measurements and clarify this in our revision.

In the simulation of the SDD model (Figure 3B), simulations at 10 min, 25 min and 120 min are shown. Assuming that 120 min corresponds to early nc14, are simulations at earlier timepoints corresponding to nc12 and nc13 indistinguishable from the profile at 120 min? This demonstration

© 2024. Published by The Company of Biologists under the terms of the Creative Commons Attribution License (https://creativecommons.org/licenses/by/4.0/).

would further support the option to merge the data from all nuclear cycles.
This is a good point. Here, we were primarily focused on showing the time evolution of the model, rather than directly mapping onto experiment. We will clarify in the revision.

The results obtained with the BcdN51A mutant show an increase in diffusion speed, while retaining similar proportions of fast and slow populations. In the slow fraction, a new population is found. Assuming that the BcdN51A molecules cannot bind specifically to DNA due to the mutation, what would this newly found population correspond to? Could the authors explore the possibility of nonspecific binding to DNA? The article would also win by discussing more on this aspect or other options.
This is an interesting question. D_{slow} for anterior nuclei of N51A mutants increases (D_{slow} from ~0.2mm^2/s to ~1.5 mm^2/s), and the proportion is similar to the slow fraction of WT Bcd in the anterior nuclei (F=50%). The D_{slow} values of bcd^{WT} suggest that 0.2mm^2/s is a result of DNA binding. For bcd^{N51A}, D_{slow} of 1.5 mm^2/s is suggestive of nonspecific interaction of bcd^{N51A} to the DNA. Such a nonspecific interaction is also noticed in the case of NLS::eGFP, where we see a significant amount (D_{slow}~ 1-1.5 mm^2/s , F=20%) of slow form in the anterior nuclei, likely due to non-specific interaction with the DNA.

It is worth noting that the inactive homeodomain of transcription factor sex comb reduced (scr) also interacts non-specifically with DNA at high concentration (Vukojevic *et al*., PNAS 2010). Non-specific interaction of eGFP fluorophore is also noted to be higher in the nuclei of AT-1 cells that suggest "obstacle-free accessible space" is low in the nuclei (Wachsmuth *et al*., JMB 2000). Therefore, though we do not understand the specific mechanism, our results for N51 mutants are aligned with previous observations of intra-nuclei dynamics.

The experimental rational behind the BcdMM reporter needs to be better explained as it is not clear. It was previously shown that the N51A mutation disturbs zygotic hb activation and Caudal gradient formation (see Figure 3 in Niessing et al., 2000). Since N51A already causes a strong phenotype by disturbing hb expression and Cad gradient formation, what is the reasoning being adding extra mutations to this background? Since the mutations in the PEST domain and YIRPYL motif are involved in cad translational repression, it would be more interesting to add them to the R54A mutation and further study the repression of cad? It would also shed light on the unexpected no difference or even decrease in diffusion in the cytoplasm of the R54A mutant which should increase if indeed the cad mRNA binding is being repressed.
Our rationale was to remove more elements of Bcd to see if there was some degree of redundancy - at least in terms of the dynamics.

The Bicoid homeodomain N51A mutation is physiologically known to cause de-repression of caudal and inhibit hunchback expression. Mechanistically, nuclear Bcd activates *hb* transcription. However, in the cytoplasm Bcd interacts with other proteins and forms a complex to de-repress *caudal*. Bcd binds to *caudal* mRNA through its HD at one end of the complex.
However, in the other end, other proteins in the complex are bound to the 5'cap region *caudal* mRNA. Our rationale for generating the MM mutation was that the N51A mutation may not be sufficient for Bcd to be released from the protein complex. Therefore, additional mutations to N51A may release Bcd from interactions with either DNA or with other proteins through PEST domain and YIRPYL motif.

Have the authors confirmed that their BcdR54A indeed inhibits cad translation?
We have not tested the eGFP:bcdR54A to inhibit *cad* translation. We will add the data in the revision.

How many embryos of BcdMM were analysed? The authors should also provide a table with all the values in SI as they have done for all the other reporters.
We will add this data with the revision.

The claims with eGFP::NLSBcdHD need to be supported by data from multiple embryos. Even if multiple ACF curves are obtained from one embryo, analysing only one embryo is not sufficient. This would clarify the fact that this reporter seems to be able to reproduce the mobility of Bcd in the nucleus.

© 2024. Published by The Company of Biologists under the terms of the Creative Commons Attribution License (https://creativecommons.org/licenses/by/4.0/).

We agree and we are arranging to collect more data. This should be completed by the end of the summer.

According to the methods, all reporters were expressed in a bcd null background, made with the bcd1 allele. This allele is also known as bcd085 and according to Driever and Nusslein-Volhard, 1988 (PMID: 3383244), this allele only causes an intermediate phenotype. This indicates that a truncated version of the protein probably still exists on the embryo. Do the conclusions obtained here still hold if a truncated version of the Bcd protein exists in addition to their reporters?
We used the bcd^{E1} mutant, a null mutant of bcd. This was used by Gregor *et al.*, Cell 2007 in their generation of the original Bcd::eGFP. We have also recently generated a more complete bcd^{KO} mutation (Huang *et al.*, eLife 2017). Our embryos do not have a clear phenotype that we can relate to the specific *bcd*⁻ background used. Nonetheless, we agree it is an important point to be clear about the genetic background and we will clarify in the revised manuscript.

Minor comments:
In line 45: "Morphogens are signalling molecules", the authors should consider removing the word "signalling" since not all morphogens are, especially the one being studied, Bicoid.
In lines 80-81 (and also throughout the text): "We measure the Bcd dynamics at multiple locations along the embryo AP-axis", should be more accurate and changed to anterior and posterior of the embryo. Using "multiple locations along the AP axis" is ambiguous and not exact for what was done.
Yes, this is a fair comment. We have edited these sections in the current manuscript.

Throughout the article, the authors refer multiple times to "modes for/of Bcd transport". Since they or others have not proven that Bcd is being transported, which would involve at least another factor, the authors should replace transport by movement, diffusion or a similar word with which they are comfortable.
We have changed transport to movement wherever relevant in the text.

Suggestion: The authors claim that the Bcd gradient is exponential up to 60% of embryo length. Would this information allow a more precise calculation of the gradient decay length in the exponential region than the 80-100μm stated on line 202?
This is an interesting point, but our results suggest that the idea of the decay length is not so applicable in the posterior region. There, the Bcd dynamics are generally quicker, thereby increasing l. Of course, we cannot discount possible spatial variation in degradation. However, in previous work, our Bcd tandem reporter (which is sensitive to changes in degradation) did not reveal spatial variation in degradation.

In lines 258-259, the sentence "Further, Bcd binds to caudal mRNA, repressing its expression in the cytoplasm" should be improved to clarify the role of Bcd in caudal mRNA translation repression and references should be added. This should also be corrected in the following paragraph.
We will add the necessary corrections in the revision.

In line 262, "mutations" should be singular since it corresponds to only one amino acid mutation.
We have corrected this.

Figure 4J needs to be corrected as the fractions of the slow and fast populations do not correspond to what is shown in Table 3. For example, Fslow fraction of AC is ~45% in the figure while it is 36% in Table 3. The problem occurs in all fractions.
We are sorry there is a mislabelling in the corresponding figure. AN is in the place of AC. We have edited figure 4J and removed the mislabelling.

In the discussion, in lines 379-380, "Given the changing fractions of the fast and slow populations in space, the interactions between the populations are likely non-linear". What is the reasoning for non-linearity and not interchangeability?
If the interactions between the two populations were linear, then the fraction in each form would be constant across the embryo. Some degree of nonlinearity is required in order to have spatially varying relative populations.

In line 432 caudal should be italicized.
We have edited this.

© 2024. Published by The Company of Biologists under the terms of the Creative Commons Attribution License (https://creativecommons.org/licenses/by/4.0/).

In the discussion, the authors conclude that "In the nucleus, the two populations can be largely (though not completely) explained by Bcd binding to DNA". The discussion would win by explaining all the possible options.
We will add the necessary changes in the discussion. This is also related to above reviewer comments.

Reviewer #4 (Significance (Required)):

The results presented in this article advance the knowledge of the field by adding data and quantifications of Bcd mobility at four locations: anterior nucleus, posterior nucleus, anterior cytoplasm and posterior cytoplasm. Until now, FCS studies have focused mostly on measuring the dynamics of Bcd in nuclei at the anterior (Abu-Arish et al. 2010; Porcher et al. 2010) of the embryo. The results are also consistent with what was previously found for eGFP:Bcd in the anterior nucleus. Still, this is not surprising as they use the same reporter as the previous studies. This article will be interesting to an audience comprising biologists and biophysicists interested in protein diffusion.
As a biologist, I do not have sufficient expertise to completely evaluate if the modelling is performed flawlessly. However, in my understanding, the FCS analysis is crucial for the results and their conclusions, hence the comment on the certainty of the existence of only two Bcd populations, though these populations being previously described with FCS. Comments from a physicist with experience in analysing FCS data are thus necessary.

3. **Description of the revisions that have already been incorporated in the transferred manuscript**

At this stage, we have only corrected the minor typos and errors. The new experiments are already planned and will be completed by the end of the summer. The other corrections largely concern rewriting and will be doable within the same timescale.

4. **Description of analyses that authors prefer not to carry out**

The Reviewers highlight a number of interesting future experiments. These include a more spatially refined dissection of the dynamics across the embryo and further analysis of the specific mechanisms of Bicoid dynamic regulation. However, as recognised by the Reviewers, such experiments lie beyond the current scope of the paper. For the revision, we will focus on validating our FCS measurements and also improving the model description.

Original submission

First decision letter

MS ID#: DEVELOP/2023/202128

MS TITLE: Long-ranged formation of the Bicoid gradient requires multiple dynamic modes that spatially vary across the embryo

AUTHORS: Thamarailingam Athilingam, Ashwin VS Nelanuthala, Catriona Breen, Timothy Saunders, and Thorsten Wohland

Many thanks for submitting your paper to us through Review Commons. We have now had the chance to read your paper, the Review Commons reports and your revision plan. We find the work potentially interesting, and think that you have laid out a sensible plan for revision. We'd therefore invite you to revise your article according to your response to the referee comments and submit the revised version to Development. At that point, we would seek input from one or more of the Review Commons referees as to how well they feel you have addressed their concerns.

© 2024. Published by The Company of Biologists under the terms of the Creative Commons Attribution License (https://creativecommons.org/licenses/by/4.0/).

If you have any questions about this, please don't hesitate to get in touch.

First revision

Author response to reviewers' comments

We thank the reviewers for their detailed comments. We have made substantial changes, including new experiments and additional theoretical considerations. We believe we have addressed all the substantive issues.

Below, we provide a point-by-point rebuttal (in blue) to the comments. Line numbers refer to the document "paper resubmission annotated".

Also note that since the FCS Methods had become very long, we have moved the more involved details (including the associated figures) to the Supplementary Materials and Methods.

Reviewer 1

1) Gradient profile quantification: Some of the conclusions made by the authors rely on the comparison between their model of gradient formation (as captured in the equations in lines 232 and 233) and the Bcd intensity profile measured in the embryos. Since the differences in gradient shape predicted by the different models are very small (see Fig. 3B, which is on a log scale and therefore emphasize small differences, and Fig. 3C), it is very important to understand how reliable the experimental concentration profiles are.

We agree that this was a problem in the original presentation of the results. It is worth noting that the key differences between the one- and two-component models are only apparent at large distances (and hence low concentrations) from the source. We discuss issues related to the modelling in later points; here we specifically address the gradient quantifications.

We performed the quantification of the gradients in a manner similar to the Gregor lab, whereby the midsagittal plane is analysed. We used 488nm illumination so our measurements are likely noisier. However, we are not investigating the variability in the gradient here, but the mean extent. In the original submission, we corrected background with a uniform subtraction, but this is not the best method.

In the revised manuscript, we have repeated the above experiments using a confocal microscope. Concurrently, we imaged lines expressing His::mCherry without eGFP under the same imaging conditions to more accurately estimate the background signal. The updated profiles are shown in Figures 3B and Figure 4B and we have updated the Methods and Supplementary Materials and Methods. This has improved the confidence in our curves, but we note there are no significant changes to the original profiles.

At the moment, I do not find the evidence that [Bcd] concentration profile is more consistent with a 2-component diffusion model than a 1-component model very strong. A few comments related to this:
1a. Line 249, it is mentioned that: "observations ... incompatible with the SDD model". Which observations exactly are incompatible with the SDD model?
The key points were in the preceding paragraph. We now move the details on this point to the Discussion (lines 456-459, 463-468, 479-485, 535-538).

1b. In Fig. 3D, only the prediction of the 2-component model is shown. What would the simple 1-component diffusion model look like? Is it really incompatible with the data?
We now provide the one-component fit to the gradient profiles (Figure 3C top). It does fit a large range of the profile, as expected as it has previously been shown to approximate well the Bicoid profile (Figure 3B). However, it clearly also does not fit the gradient as well in the embryo

© 2024. Published by The Company of Biologists under the terms of the Creative Commons Attribution License (https://creativecommons.org/licenses/by/4.0/).

posterior as the two-component model (Figure 3D). We expand on the differences between one- and two-component models in the Discussion (lines 463-469 and lines 479-485).

Regarding the FCS data, we have now added the one-particle fits in the Supplementary Materials and Methods (Supplementary Figure S1D-E; Supplementary Table 3). A two-particle fit is clearly an improvement (see also related response to Reviewer 2).

1c. Line 243: "The increased fraction in the fast form ... consistent with experimental observation of Bcd in the most posterior" (Mir et al.)". I am not sure how this is significant, since the simple model also predicts there will be Bcd in the posterior - the only difference is how much is there (as shown in Fig. 3C), and it's a very small difference.
The absolute differences in steady-state are not large between the two models, but due to the observed clustering (Mir et al. 2018), even small differences can have very large effects. Importantly, a concern raised previously about the SDD model is that it takes a long time to reach steady-state (Bergmann *et al.* PLoS Biology 2007), especially in the posterior. Our two-component model displays high concentration in the posterior much faster than the SDD model (Figure 3E). We have improved this panel to make this difference more apparent.

1d. Since the difference between models is in the posterior region where Bcd concentration is very low, when comparing the models to the data the question of background subtraction is essential. How was the subtracted background (mentioned line 612) estimated?
See above response to the first comment. We now include extended Methods (lines 704-711) and improved estimation of error.

1e. Along the same line, were the detectors on the Zeiss LSM analog or photon counting detectors, and how confident can we be that signal is exactly proportional to concentration?
We used PMTs and did not directly do photon counting. But the intensity is still proportional to the concentration. It is possible to estimate the absolute concentration value, *e.g.*, Zhang et al., 2021 (https://doi.org/10.1016/j.bpj.2021.06.035). However, our main conclusions – especially regarding the spatially varying Bcd dynamics – are not dependent on this.

1f. Can the gradients created by the two Bcd mutants (Fig. 4B) be quantified as well, and are they any different from the original Bcd gradient?
We provide the gradient quantifications of the *bcd* mutants in Figures 4B. We see no significant change in the mutant gradient profile (eGFP:Bcd^{N51A} and eGFP::Bcd^{R54A}) with that of the eGFP::Bcd.

1e. What is the pink line in Figure 5C (I am assuming the green one is the same as in Fig. 3D)? It could be better to not use normalization here, or normalize everything respective to the eGFP::Bcd data to make comparison in relative concentrations in the posterior for different constructs more evident (also maybe different colors for the three different data sets would help clarity).
We agree and have improved the data visualisation. For clarity, we have removed the model fits, as the point here is that the gradient profiles are clearly distinct under the different conditions.

1f. Discussion, lines 402-403: Does the detailed shape of the Bcd in the posterior region matter at all, since the posterior is not a region where Bicoid is active, as far as we know? Could a varying Bcd dynamics have other consequences that would be more biologically relevant?
Bcd is known to act at 70% EL (Singh *et al.*, Cell Reports 2022). So, the gradient is relevant for a large extent of the embryo length, though it is not known if there is any effect in the most posterior region. We have clarified this in the Discussion (lines 445-447).

2) Model for gradient formation (lines 231-238):
2a. Whether the molecules of Bcd can change from their fast to slow form is never questioned. How do we know (or why might we suspect) they do exchange?
Within the nucleus, and based on our mutant data, a likely scenario is that the fast/slow forms correspond to unbound/bound DNA states. Reversible DNA (un)binding is common, consistent with our model.

In the cytoplasm, the dynamics are less clear. Bcd can bind to cytoskeletal elements (Cai *et al.*, PLoS One 2017) as well as to *caudal* mRNA. Therefore, it seems reasonable to have different effective dynamic modes – yet, how such switching occurs remains unknown.

© 2024. Published by The Company of Biologists under the terms of the Creative Commons Attribution License (https://creativecommons.org/licenses/by/4.0/).

Our model approximates multiple dynamic modes that are integrated to drive Bcd motion. Including switching between states is a reasonable assumption based on what is known about cytoskeletal and protein dynamics, but we do not have a specific mechanism.

It is challenging to estimate specific rates of switching, partly because the dynamic changes also depend on the diffusion – which itself is changing. For now, we believe our level of abstraction is appropriate given what is known about the system. It will be very interesting to explore the specific interactions underlying such behaviour in the future, but that is beyond this current manuscript.

We include a caveat about this in the Discussion (lines 456-459, 463-469, 535-538).

2b. The values used in the model for alpha, beta_0 and rho_0 should be mentioned. Maybe having a table with all the parameters in the method section, or even in the supplementary section, would help. The exact values of alpha and beta matter, because if they are large (fast exchange) a single exponential gradient is to be expected, if they are 0 (no exchange) a double exponential gradient is to be expected, with intermediate behavior in between. Which case are we in here?
We agree and have added a more complete table in the Methods (Supplementary Table S7). The parameter values are such that the gradient is exponential like in the anterior domain, with a shift to longer length scales in the posterior. Therefore, our model is consistent with an intermediate level of switching. This is also supported by the observation that the fraction in slow/fast forms are around 40/60, except in the posterior cytoplasm (Figure 2D).

3) Discussion about anomalous diffusion (lines 386-388): The 2-component model used by the authors to interpret their FCS data seems very well justified here (excellent fits with very small residuals). I agree with the authors' conclusion that "the dynamics of Bcd within the nucleus are more complicated than a simple model of bound versus unbound Bcd", but I don't see how that can lead to a diagnostic of anomalous diffusion instead. Maybe it is just a matter of exactly explaining what is meant by anomalous diffusion here (since this term is often used to mean different things). A more likely scenario I think, is that there are more than just two Bcd components in the system.
This is a good point, and we can't easily differentiate two/multi- component fits from anomalous diffusion ones. This is a known problem. But we have recently shown in a collaboration with the Laurent Heliot lab (Furlan et al, Biophys J 2019), that anomalous diffusion is a good stable indicator of changes, even if it might not be the right model. We use anomalous diffusion as it stably predicts changes. We do not claim, however, that diffusion is anomalous. We have improved the discussion of these points in the revised manuscript (lines 471-485).

4) Line 440 and after: What is the evidence that the transition between the two forms might vary non-linearly with Bcd concentration? How would that help adapt to different embryo sizes? It would be good to be more explicit here instead of just referring to another paper.
Bicoid is unlikely to simply depend linearly on concentration as in that case the ratio of fast to slow forms would be roughly constant across the embryo. Related to the above comment, it is important to emphasise that we are using a phenomenological model, not one based on a specific mechanism.

We do not explicitly argue that the two forms of Bcd aid scaling to embryos of different size. That is an interesting idea, and it could be tested in *fat2*::RNAi lines, which lay small eggs (Huang *et al.*, eLife 2020).

5) Since an important aspect of this work is the study of different Bcd constructs *in vivo*, it is important that these constructs are very clearly described, so the section on the generation of the fly lines (Methods) should be expanded. In particular:
5a. It seems that the eGFP:: NLS control used here was different from that first described in Ref. 64 (and used for FCS experiments in Ref. 30 and 36)? If so, what NLS sequence was used here, and precisely what type of eGFP was used (in particular, was the A206K mutation that prevents dimerization present in the eGFP used)? If it is the same construct as in Ref. 64, it should be mentioned explicitly.
We have added how eGFP::bcd^{N51A}, eGFP::bcd^{R54A}, eGFP::bcd^{HD}:NLS, and eGFP::bcd^{MM} are generated in the method section (lines 595-629). The eGFP:NLS control is generated in the same way as described in (Gregor *et al.*, 2008) with same NLS sequence. Further, the eGFP sequence is from construct eGFP::Bcd pCaSpeR7 (Gregor *et al.*, 2007).

© 2024. Published by The Company of Biologists under the terms of the Creative Commons Attribution License (https://creativecommons.org/licenses/by/4.0/).

5b. Were the mutant N51A and R54A lines gifts as well, or have they been described before? If so, previous publications should be referenced. If not, how the plasmid was introduced in the embryo should be briefly explained.
We clarified the information on the generation of transgenic fly lines (eGFP::bcd^{N51A}, eGFP::bcd^{R54A}, eGFP::bcd^{HD}:NLS, and eGFP::bcd^{MM}) substantially in the Methods section of the manuscript (lines 627-630).

6) Concentration calibration measurements (Methods Fig. 2, line 568 and on). It is well known that background noise is going to interfere with the measurement of N when the signal becomes equivalent to the background noise (Koppel 197, Phys Rev A 10:1938-1945, and for a recent discussion of this effect for morphogens in fly embryos: Zhang et al., 2021, Biophysical Journal 120,4230-4241). It is almost certain that in the low signal regions of the embryo (e.g. posterior cytoplasm) this is affecting the reported concentration, and should be at least acknowledged.
We now provide the information on the background noise and its influence on the posterior measurements in the Methods section under the title "*Determination of Bicoid concentration in the posterior domain of the embryo*". Further, we caveat our posterior results on line 176-180.

7) Reference 3 is mis-characterized in two different ways in the manuscript:
7a. Line 50: The conclusion in Ref. 3 was not that the gradient was due to a diffusive process, on the contrary Gregor et al. argued that Bcd was too slow to form such a long-range gradient by diffusion. Studies that do present data consistent with a morphogen gradient formation mechanism driven by diffusion are reference 5, reference 30, Zhou et al., Curr. Biol. 2012;22(8):668-75 and Müller et al., Science 336 (2012) 721-724.
Gregor *et al.*, do not argue against a diffusion process – indeed, they utilise a SDD model in their paper. However, they do extensively discuss how the predicted dynamics from the SDD model are not compatible with the time scale of gradient formation as observed after n.c. 13 (Little *et al.*, PLoS Biology 2011). This problem was resolved to some degree by FCS measurements of Bcd (e.g., Dostatni lab, Development 2011) and the use of a Bcd tandem reporter which showed that production and degradation change during n.c. 14 (Durrieu *et al.*, MSB 2018). We now add an additional sentence to make it clear there is ambiguity about interpretation of the SDD model, even for diffusive systems (line 51-53 and 463-469).

7b. The diffusion coefficient estimated from FRAP measurements and reported in Ref. 3 (D = 0.4 micron^2/s) is mentioned a couple of times in the manuscript (line 66, line 395, line 411). However, this number is simply incorrect. When fast components (such as the ones clearly detected here by FCS) are present, they diffuse out of the photobleached area during the photobleaching step. If that is not corrected for during the analysis (and it wasn't in Ref. 3), then the recovery time measured is just equal to the photobleaching time, and has nothing to do with either the fast or slow fraction of the studied molecule - it has no other meaning than to give a lower bound on the value of the actual effective diffusion coefficient of the molecule. This effect (called the halo effect) is well known in the FRAP community (see e.g. Weiss 2004, Traffic 5:662-671), it has been experimental demonstrated to occur for Bcd-eGFP in the conditions used in Ref. 3 (Reference 30), and the actual diffusion coefficient that should have been extracted from the data presented in Ref. 3 has been recalculated by another group to be instead D = 0.9 micron^2/s (Castle et al., 2011, Cell. Mol. Bioeng. 4:116-121). It would therefore be better to report the corrected value from Castle et al. to help the field converge towards an accurate description of Bcd mobility.
We fully agree and now use the improved FRAP estimated value for Bcd and include the appropriate references. It is noteworthy that the FRAP measurements and our "slow" fraction are similar in magnitude. In addition, we carried out the SPIM-FCS measurement of eGFP::Bcd where the slow diffusion values of FRAP and point FCS are reproduced (Supplementary Figure S3). Returning to the above point (7a), the conclusion in the Gregor *et al.*, paper was based on FRAP measurements, which did not capture the fast dynamics.

Minor comments and suggestions:

8) Figure 1: From panel A, it seems that what is called "Anterior" and "Posterior" is about 150 micron away from the embryo mid-section, i.e. about 100 micron from either the anterior pole or the posterior pole (so not the tip of the embryo, but somewhere in the anterior half or posterior half). Maybe this should be made clear in the text.

© 2024. Published by The Company of Biologists under the terms of the Creative Commons Attribution License (https://creativecommons.org/licenses/by/4.0/).

We have made changes in Figure 1A to indicate the region within which the FCS measurements are carried out. We have added the relevant details in the legend of Figure 1 (line 155).

9) Fig. 2A; It might be good to put this graph on a log scale, so that cytoplasmic values are seen more clearly. Also, what about reporting on nuclear to cytoplasmic ratios?
We have reworked this graph. Now, Figure 2A and E are on log scales. We also include as an inset the n/c ratio in anterior and posterior in Supplementary Figure S1H.

10) Fig. 2: It could be interesting to plot D_effective as a function of the measured concentration of Bicoid in different locations, since the (interesting) suggestion is made several time that [Bcd] could the a determinant of the protein mobility.
By measuring at different locations, our work provides an indication that Bcd diffusion is connected to the the local Bcd concentration. We now show this in Figure S13E-F. This is a qualitative data (we could not ascertain the precise distance from the anterior pole) showing the variation of D with respective the FCS carried out at four locations in the embryo. To extend this to a rigorous model would require substantial new measurements along the whole length from multiple embryos. While interesting, this represents a very large investment of time and lies beyond the current manuscript.

11) Figure 3B&C: Is the curve for 2-component diffusion (without concentration dependence) for steady-state missing?
In steady-state, it had very similar concentration to our full two-component model (as average diffusion is the same), so the curves overlapped. We have now altered this figure and more complete modelling data is in the Supplementary Materials and Methods.

12) Lines 78 and 471: What do the authors mean by "new reagents"? The word reagent evokes a chemical reaction, but there are none here. Do the authors mean new constructs? or new mutants?
We have changed “new reagents” to new Bcd mutant eGFP lines” elsewhere in the text.

13) Lines 57-59: Another good reference for FCS measurements performed to study the dynamics of a morphogen (in this case Dpp) is Zhou et al., Curr. Biol. 2012;22(8):668-75
We added this reference.

14) Lines 109-111: A word must be missing. Precisely determined what?
We meant precisely measure within cytoplasm, and nuclear compartments and also during interphase stages. We have changed to “precisely measure in the cytoplasmic and nuclear regions during the interphase stages of nuclear cycles (n.c.)12-14.” in lines 126-128.

15) Line 278: The increase in the slow mode is expected. Maybe explicitly mention why.
In line 337, we have added “due to the loss of Bcd binding to the DNA”.

16) Line 282: "with the fast component increasing", maybe replace with "with the diffusion coefficient of the fast component increasing" or "with the fraction of the fast component increasing".
We have changed line 340-341 “with the diffusion component of fast component increasing towards the posterior”.

17) Line 517: Is there a reason why the dorsal surface is always placed in the coverslip?
We wanted to standardise the imaging conditions as much as possible to minimise embryo-to-embryo variation. We have added these details in lines 122-124, and 635-636 in Methods.

18) Line 524 and on: FCS measurements: What was the duration of each individual FCS measurement? It is great that the exact number of measurements are reported in the supplementary!
Thank you for the complement. Typically, cytoplasmic measurements are 60secs and nuclear measurements are 20-40s. We have added this in line 640-642.

19) An Airy unit of 120 um seems large in combination with an objective with a NA of 1.2, is there a reason for that? What was the radius of the resulting detection volume?
In the particular Olympus confocal microscope used, there is a 3x magnification stage. This leads to the change in the Airy unit. Otherwise, it would be 40 μm.

© 2024. Published by The Company of Biologists under the terms of the Creative Commons Attribution License (https://creativecommons.org/licenses/by/4.0/).

20) Thank you for detailing the reasons behind the choice of excitation power, an important and often omitted details. Where in the excitation path were the values of the laser power measured (before or after the objective?)?
Thank you for the complement. The laser power is measured before the objective. We removed the objective and measured the laser power in the objective path. We have added this detail in the legend of Figure S10.

21) Line 585: "since the brightness of eGFP::Bcd..." do the authors mean the molecular brightness of a single eGFP::Bcd molecule, or the total fluorescence signal?
It is the total fluorescence signal. We have this is in the extended FCS Methods in the Supplementary Materials and Methods.

22) It would be good for reference to mention the approximate value of the molecular brightness recorded for these eGFP constructs at the laser power used.
We found the eGFP brightness is approximately 600-800 counts per sec/molecule in the anterior and posterior cytoplasm.

23) Reference 766: The year (and maybe other things) is missing.
We have corrected this reference.

24) Figure 2 (Methods): The concentrations shown on the figure should be in nM not uM.
Thanks for noticing - we have changed.

Reviewer #1 (Significance (Required)):

Strengths:
Very careful and systematic study of Bcd's dynamics in the early embryo
Use of several mutant and truncated forms of Bcd to pinpoint the importance of the DNA binding domain in setting this dynamics
Uncovers a previously unknown change in Bcd dynamics from the anterior to the posterior of the embryo
Modelling of the Bcd concentration gradient shape taking into account the measured dynamics

Limitations:
The quantitative comparison between modelled and measured gradient could be improved.
The discussion of the biological implications of the work is limited

Advance:
Uncovers a previously unknown change in Bcd dynamics from the anterior to the posterior of the embryo. This raises very interesting questions about molecular mechanisms involving Bicoid.
Other studies (cited in this manuscript) reported on Bcd dynamics, but the present study represents a very welcome expansion of these earlier studies, by looking at spatial dependence and by examining several Bcd constructs.

Audience:
- Somewhat specialized, as this work should firstly be of interest to scientists studying morphogen gradients. However, it is also a beautiful example dynamical studies in vivo, so it will also be of interest to experimental physicists studying protein motions in vivo (a rather large community).

We thank the Reviewer for the critical but positive comments on the manuscript. We believe that the above changes have helped to tighten the key manuscript results.

Reviewer #2 (Evidence, reproducibility and clarity (Required)):

SUMMARY

In this study, Athilingam et al. investigated Bicoid (Bcd) protein dynamics along the anterior-posterior axis of Drosophila embryos. They performed Fluorescence Correlation Spectroscopy (FCS) experiments to analyze Bcd protein diffusion and found that Bcd had a 1.7-fold higher mobility in

© 2024. Published by The Company of Biologists under the terms of the Creative Commons Attribution License (https://creativecommons.org/licenses/by/4.0/).

the posterior compared to the anterior. The authors also generated Bcd homeodomain mutations and analyzed their impact on gradient formation. They found that the BcdN51A mutation exhibited altered nuclear dynamics with faster diffusion, while cytoplasmic dynamics remained unchanged. BcdR54A embryos showed dynamics more similar to the wild-type Bcd, with a minor decrease in slow diffusion in the posterior region. Interestingly, the Bcd homeodomain alone, when fused to eGFP-NLS, was able to replicate the observed Bcd protein dynamics, particularly in the slower diffusive mode. The paper provides evidence that the Bcd homeodomain has a significant influence on protein dynamics and suggests a complex interplay of interactions between Bcd, nuclear DNA, and cytoplasmic RNA that regulate Bcd diffusion and function.

Overall, the data and methods presented in the paper are clearly described. The experiments are adequately replicated, and the statistical analysis appears sufficient. Prior studies are referenced appropriately, and the claims and conclusions are mostly supported by the data. However, the following points should be addressed to corroborate the conclusions:

We thank the reviewer for the appreciation of our work. Below, we address the specific issues raised.

MAJOR POINTS

1) FCS measurements and fits
a) Please state the duration of each individual FCS measurement.
In the cytoplasm, the measurements were carried out for 60 secs and in nuclei it is between 20-40s. We could not measure for 60s in the nuclei as the nuclear position fluctuates from its initial position. We have added the duration of FCS measurements in the Methods (lines 640-642).

b) The authors acknowledge potential issues with fluorophore photophysics and use different lag time ranges for the calibration dye Atto-488 (0.001 ms in Method Fig. 2) and eGFP (0.1 ms in the main figures). Given the strong influence of different parameters on data interpretation and conclusions, Method Fig. 2 should be repeated with purified eGFP. This is particularly relevant for the noisy FCS measurements in posterior regions.
Performing the experiment with purified eGFP will be a volume calibration. We routinely performed this before each imaging session, and that should be fluorophore independent. As noted by Reviewer 1, it is also important to be clear about background correction.

We have measured eGFP *in vitro* and used this for concentration calibration (Figure S12) to calculate the triplet values (Figure S11). Our Bcd::eGFP concentration estimation from the calibrated Atto-488 dye did not show drastic difference with that of eGFP calibration.

We use 0.1 ms to start, as at that point any contribution from the photo-physics should have decayed (0.1 ms is about 3-5 times the day rate of the photophysical process, Sun *et al.*, Analytical Chem 2015).

c) Please explain why no data is shown for "AN" around 0.1 ms lag time in Fig. 1B in contrast to all other figures.
We now include the data for AN from 0.01 in the revised Figure 1B and Supplementary Figure S1.

d) Please state what the estimated diffusion coefficients with one-component model fits are. Please also explain why the fits in Fig. S1E do not reach a value of 1 and why they plateau higher than the experimental data at long lag times. Please constrain the fits to G=1 at 0.1 ms tau and G=0 at 1 s tau to make a fair comparison.

We thank the reviewer for the suggestion. Supplementary Figure S1D-E is now changed. The one-particle and two-particle fits are compared with constrained fits keeping G(0) and G(∞) fixed. We also compared the quality of the fit using reduced □^{2} analysis in new Supplementary Table 3. It is clear that the one-component fit does not accurately reflect the measurements.

e) Please assess the validity of all multi-component fits by comparing the relative quality of the models to the number of estimated parameters using the Akaike information criterion or similar approaches.

© 2024. Published by The Company of Biologists under the terms of the Creative Commons Attribution License (https://creativecommons.org/licenses/by/4.0/).

We used reduced χ^2 analysis to assess the quality of fits in Supplementary Table 3.

f) Please also present the Bcd-GFP fits with 0.001 ms that are mentioned in line 590, and present the results for the data that did not give comparable tau_D1 and tau_D2 values mentioned in line 593.

We now provide fits from 0.001ms in the Supplementary Figure S2. We have fitted each of our measurement with 2-Particle 1-triplet (variable triplet) to test the accuracy of our TauD values. We have plotted all our TauD1 and TauD2 values with respect to the obtained triplet values. We also provided weighted mean TauD1 and TauD2 values. Our TauD1 and TauD2 values did not vary from the non-triplet fits given in Supplementary Tables 1 and 2.

3) Bicoid gradient and modeling
a) Little et al. 2011 observed that the Bcd gradient decreases around n.c. 13. Can the authors of the present work observe a similar concentration decrease using FCS? This is important to i) validate the FCS concentration measurements, and ii) to resolve the controversy regarding "previous claims based on imaging the Bcd profile within nuclei, which predicted decrease in Bcd diffusion in later stages".
This is a good point regarding conclusions from the previous literature. The Little *et al*. paper inferred that diffusion had to decrease from fitting to the gradient profiles. However, subsequent analysis from our lab (Durrieu *et al.*, MSB 2018 - which uses a different method involving a tandem reporter for Bicoid - and this manuscript) strongly suggest that Bicoid remains dynamic, at least through n.c. 13 and early n.c. 14.

One way to test this is to use SPIM-FCS, where longer time courses can be taken (though with slower time resolution in the FCS). As SPIM-FCS is limited by the camera speed, we largely only collect the slow fraction. We have performed SPIM-FCS and do not see significant changes in the ACF during n.c. 13 and early n.c. 14. However, more work is required to validate these measurements, more suitable for a follow-up publication. From our SPIM-FCS, we do see clear and reproducible variation in the diffusivity across the nuclear envelope. We now include this in the manuscript (Supplementary Figure 3), as it provides support for their being distinct dynamics domains of Bcd.

We have extended the Discussion to make the results clearer in terms of previous models and literature (lines 449-469).

b) Please explain why the experimental Bcd-GFP gradient data does not reach a value of 1 (e.g. in Fig. 3D) despite normalization. Please also explain why the fits become flatter in Fig. 5B compared to the steep fit in Fig. 3D.

Both lines were measured under identical conditions. Therefore, we previously normalised to the maximum value of both experiments. We have now redone, keeping the intensity values for individual experiments. In Figure 3D (now 3C), we originally used a point source, but in Fig. 5C we included a broader possible domain for Bcd production as a fitting parameter (see Supplementary Materials and Methods, where model details are further provided). Note, we also account for the Bcd folding time, which does impact the observed (compared to actual) profiles (see Figure 8, Little *et al*. PLoS Biology 2011).

The flatter curve in Figure 5 is the line with eGFP tagged to a NLS alone. We provide new gradient profiles in Figure 5, which show clear differences in the gradients of different constructs.

c) For modeling, please take into account observations that the Bcd source is graded with a wide distribution (30-40% EL, see Spirov et al. 2009, Little et al. 2011, Cai et al. 2017 etc.). The extent of the source used in the present work (x_s=20 um, line 620) is at least five times too small.
Care must be taken in defining the source extent. The most careful measurements are reported in Little *et al.*, PLoS Biology 2011 who performed single molecule FISH. They conclude “We demonstrate that all but a few mRNA particles are confined to the anterior 20% of the egg”. Further, the peak in the particle density is around 20-30mm from the anterior (Figure 3, Little *et al.*, PLoS Biology 2011), with the vast majority of counts being within 10% (<50mm) of the anterior pole. Further, Durrieu *et al*. MSB 2018, showed using a Bcd tandem reporter that there was unlikely

© 2024. Published by The Company of Biologists under the terms of the Creative Commons Attribution License (https://creativecommons.org/licenses/by/4.0/).

to be an extended gradient of *bcd* mRNA (maximum extent of around 50μm). We originally used a simple source domain, which was arguable a little narrow, but not significantly so. We now keep the source domain as a fitting parameter, with a typical fitted value around 30μm (Supplementary Table 7), which matches well with Little *et al.*, and Durrieu *et al.*.

d) Please discuss in the paper how well the simulations in Fig. 3B agree with the experimental data. We now provide these details in the revision, including fit measures in the Supplementary Materials and Methods (Supplementary Figure S5). We have improved Figure 3, which more clearly shows the fit quality across the embryo AP axis for different models.

e) Please provide a precise estimate for the statement "Even with an effective diffusion coefficient of 7 μm2s-1, few molecules would be expected at the posterior given the estimated Bcd lifetime (30-50 minutes)" to turn this into a quantitative argument. How many molecules are expected to reach posterior in which model, and how does it compare to experimental observations?
The time taken for a molecule to diffuse an average distance x_{rms} is given by $t \sim x_{rms}^2/(cD)$, where the constant c depends on the system dimensionality. Taking D~7 $\mu m^2 s^{-1}$, x_{rms}~*450mm* and c=4 (representing effectively 2D diffusion near the surface) gives t~2 hours. This is 2-4 times the Bicoid lifetime. Therefore, few molecules are expected to reach the posterior before degrading if there is not a fast pool of Bcd present. We present this in lines 290-296.

f) The sentence "we find that a model of Bcd dynamics that explicitly incorporates fast and slow forms of Bcd (rather than a single "effective" dynamic mode) is consistent with a range of observations that are otherwise incompatible with the standard SDD model" needs to be toned down and corrected since a simple SDD appears to be sufficient to account for the observed gradients. If the authors disagree, please specifically point out in the paragraph around line 249 what observations exactly are incompatible with a standard SDD model.

This is similar to the point raised by Reviewer 1. While the standard SDD model can explain the overall gradient shape, it is not compatible with the observed time scales and Bcd puncta tracked in the posterior pole. We have removed this particular sentence, as we realise it is distracting. We have improved the Discussion around this point to make the distinctions between the models clearer.

5) Data presentation
a) In line 27 and 122 it would be better to rephrase the wording "find/found" and give credit to previous papers that first made these observations.
We have edited in the revision.

b) For the statement "This suggests that the dynamics of the fast fraction were not captured by previous FRAP measurements", please explain why this should not be the case even though the fast fraction is shown to be larger than the slow fraction in the current work.
We have edited in the revision - see also Reviewer 1 comment.

c) Similarly, the sentence "The dynamics of the slower mode correspond closely to measured Bcd dynamics from FRAP" likely needs to be corrected since it neglects the contribution of the faster mode, which is fluorescent as well and should also contribute to the dynamics from FRAP.
This is similar to the point raised by Reviewer 1 and have edited in the revision.

d) In the absence of further evidence (see above), the sentences "We establish that such spatially varying differences in the Bcd dynamics are sufficient to explain how Bcd can have a steep exponential gradient in the anterior half of the embryo and yet still have an observable fraction of Bcd near the posterior pole" and "These results explain how a long- ranged gradient can form while retaining a steep profile through much of its range" in the abstract need to be toned down.
Our results show that there are (at least) two dynamic forms of Bcd and, combined, they are capable of forming a long-ranged gradient while also ensuring the gradient remains steep in the anterior (because the diffusion coefficient itself varies across the embryo). We have adjusted the second statement to make it less definitive - making clear that it is an output from the model. Further, we have emphasised that our model is phenomenological (lines 467 and 484).

© 2024. Published by The Company of Biologists under the terms of the Creative Commons Attribution License (https://creativecommons.org/licenses/by/4.0/).

e) The authors state that "However, we show that eGFP::Bcd in its fastest form can move quickly (~18 μm2s-1), and the fraction of eGFP::Bcd in this form increases at lower concentrations", but this has not been directly shown. Please tone down this statement or directly test the prediction that Bcd has a higher fraction of the fast form in earlier nuclear cycles when Bcd concentration is smaller.
We do show that the diffusion coefficient is larger at lower concentrations (Figure 2). However, this is correlative, and we do not directly test this. We have added an additional sentence qualifying this statement (lines 456-459). The suggested experiment will be very helpful, but comparing across embryos with different concentration levels represents a project in its own right.

MINOR POINTS
1) Introduction
a) Please explain explicitly what exactly the contention in Bcd, Nodal and Wingless dynamics is in the cited references.
A full discussion of these controversies would make an interesting review article in its own right (indeed, one of us (Saunders) has written such a review focused on Bicoid). We agree that adding some detail is helpful, and so we highlight one of the controversies regarding Dpp transport (lines 57-60).

b) In line 95, it would be better to state that this is a variation of the SDD model rather than "a new model".
We changed from “a new model” to “an improved version of SDD model” in the current version of the manuscript.

2) Methods
a) The authors state that "The same software was also used to calculate the cross-correlation function", but I couldn't find any cross-correlation analyses. Please clarify.
This is a typo; there is no cross correlation. We changed this to the autocorrelation function.

b) Please correct the "uM" typo to "nM" in the legend of Figure S12A.
We have changed this in the current version.

c) In the sentence "Further, since the brightness eGFP:Bcd in the anterior and posterior cytoplasm is lower compared to the nuclei", "brightness" probably needs to be changed to "concentration" since the molecular brightness is unlikely to change.
We have edited this.

d) Please explain the background-correction method mentioned in line 612. Please also state at what temperature the experiments were performed.
We have included better background correction in the revision Methods (see Reviewer 1) lines 704-711. The measurements are carried out at 25°C.

3) Results
a) Please provide labels for anterior, posterior, dorsal and ventral in Fig. 1A.
b) Please explain the colors in Fig. 5C.
c) Please explain the dashed lines in Fig. 3C.
We have edited the figures as requested.

OPTIONAL
1) If possible, it would be helpful to mention whether the transgenic animals have any abnormal phenotypes or whether they can rescue the bcd mutant.
We are in the process of testing the mutants in bcd mutant background. The eGFP::bcd mutants generated in the study are characterised previously; *i.e.*, bcd^{N51A}, bcd^{R54A} (Niessing et al.,2000) and bcd^{MM} (Niessing et al., 2002, 1999). We will report mutant characterisations in subsequent works.

2) To validate the concentration measurements, it would be ideal if the authors could determine the Bcd concentration gradient using FCS along the anterior-posterior axis. This would also address whether there are further unexpected changes in diffusivity in medial regions and along the anterior-posterior axis that would have to be considered for modeling.

© 2024. Published by The Company of Biologists under the terms of the Creative Commons Attribution License (https://creativecommons.org/licenses/by/4.0/).

We show an example of such measurements in Figure S13. We have shown ACF curves and 2-particle fits from one embryo at n.c.13 in 3E and 3F. We acknowledge that the amplitude G(0) of ACF curves drops down at very low concentration in the posterior cytoplasm. Therefore, accurate measurement of concentration in the posterior nuclei and cytoplasm is challenging. The normalised curves in Figure S1 show the diffusional variability of Bcd across the embryo. We also quantified FCS noise in the embryo using Histone::mCherry. The noise is uncorrelated compared to Bcd; however, the noise level in the posterior is similar with the Bcd signal. Further quantification from multiple embryos is a challenge. We have generated this new data in an independent lab (Frietsche lab, Oxford). Given the consistency in our results between different setups, we believe our results are sufficiently robust.

3) Local photoconversion experiments, e.g. in Bcd-Dendra2 embryos if available, would provide compelling support for the relevance of the measurements in the current work.
This is a nice idea, but this would represent a substantial project in its own right and lies beyond the current work.

Reviewer #2 (Significance (Required)):

The paper investigates the diffusion dynamics of Bicoid (Bcd), a transcription factor crucial for establishing the anterior-posterior axis during Drosophila embryogenesis. The authors utilize Fluorescence Correlation Spectroscopy (FCS) and various Bcd mutants fused with eGFP to understand the role of Bcd's homeodomain and other domains in its nuclear and cytoplasmic diffusion dynamics. They performed elegant experiments using insightful transgenic lines, showcasing well-designed and well-executed methodology. The paper is very nice to read, with a clear and engaging writing style and excellent presentation of the data, making it easy to follow and understand their findings.
We thank the Reviewer for the overall positive view on the manuscript.

However, there are a few limitations to consider. First, the paper does not provide evidence for the switching between slow and fast populations central for their modeling, leaving an important aspect of the dynamics unexplained. Second, there are doubts regarding the accuracy of the model used to fit the FCS data (see detailed comments in the section "Evidence, reproducibility and clarity"), underscored by the statement "While the increase in the slow mode was expected, the reason for the change in the fast mode is less clear". Third, the relevance of a potentially higher Bcd mobility for gradient formation remains unclear. For example, the fit in Fig. 3D deviates substantially from the data around 0 um embryo length, which is likely even larger than the error expected from a "simple diffusion" fit at the posterior end of the embryo. In addition, in Fig. 3C the lines for the different models appear to be indistinguishable given the noisy measurements, calling the relevance of the findings into question.
This concern is similar to that raised by Reviewer 1 regarding the accuracy of the model. We emphasise that the model is a phenomenological approach to contextualise our results, rather than meant to provide a mechanistic understanding of the dynamics. We have clarified this in the revision to hopefully make clear these distinctions.

Overall, the paper extends our knowledge by providing new insights into the role of the Bcd homeodomain in determining Bcd gradient formation. The paper highlights that homeodomain interactions in the cytoplasm and nuclei are significant contributors to determining Bcd dynamics. Additionally, the paper suggests that additional components within Bcd itself or other proteins in the cytoplasm affect Bcd dynamics at different Bcd concentrations. The paper will be of interest to a broad audience in developmental biology, molecular biology, and biophysics. Researchers studying transcription factor dynamics, morphogen gradients, and Drosophila embryogenesis will find this study particularly valuable. While the study is primarily focused on basic research, the insights gained on Bcd diffusion dynamics and the role of the homeodomain may contribute to a broader understanding of transcription factor regulation and function in other systems.
We thank the Reviewer for the critical but overall positive review.

Reviewer #3 (Evidence, reproducibility and clarity (Required)):

The manuscript presents experiments that combine a number of techniques to produce an extremely detailed view of Bcd gradient formation in the early Drosophila embryo. The authors

© 2024. Published by The Company of Biologists under the terms of the Creative Commons Attribution License (https://creativecommons.org/licenses/by/4.0/).

provide both empirical and theoretical evidence that support a conclusion that Bcd has both slow and fast moving forms, that the proportion of fast vs slow Bcd is higher at the posterior of the embryo, and that a theoretical model involving both fast and slow moving components fits empirical observations substantially better than a simple diffusion model. Next, they extend the work by investigating what functional motifs of Bcd are required for gradient formation. They demonstrate that a missense mutation N51A in the homeodomain (HD) that affects both DNA binding and cad regulation has major effects on Bcd gradient formation, while the R54A mutation that only affects cad regulation does not. They further show that a protein composed of only an NLS and the Bcd HD can form a gradient that is similar to full-length Bcd, although the two-component dynamics are not recapitulated by the HD-only form.

In my estimation the experimental work is rigorous and the results fully support the conclusions of the authors. I was surprised, however, that the HD-only form localizes via very different and simpler dynamics than does full-length Bcd, but nevertheless forms at least a qualitatively similar gradient. That leads to the question as to whether the existence of the fast and slow forms and their different ratios in different parts of the embryo actually are physiologically relevant. I don't see a straightforward way to test this experimentally, because the mutations that effect Bcd gradient formation also affect essential functions of the protein that if abrogated produce severe downstream effects on embryonic development and lethality. However I would like to see this point at least addressed in the discussion. The data and the methods are presented in such a manner that they can be reproduced, and the number of replicates and statistical analysis is overall robust.
We thank the Reviewer for the positive and constructive review. They, like both previous reviewers, raise the issue of the model and how it fits with the data. As outlined above, we have improved this part of the data presentation and also the Discussion to make sure the main results are clear. We have also substantially extended the Supplementary Materials and Methods on the modelling.

We agree that the underlying importance of the different dynamic forms of Bicoid – and why they change across the embryo – remains unknown. We believe that our careful characterisation of such behaviour is important nonetheless, as it reveals that: (1) morphogen dynamics are more complicated than typically modelled, and this may be just as relevant for ligands moving through extracellular space; and (2) dynamics can vary in space/time, providing an additional possible mechanism of control for regulating morphogen gradient profiles.

Of course, we would like to explore potential physiological relevance. Further exploration of the homeodomain and its role in regulating dynamics is a potential route, but that belongs in future work.

Minor comments:

The presentation of the graphical data measuring Bcd levels along the a-p axis (Fig 1C, 1D, 4C-F and others) needs to be improved, because the grey lines that represent ACF curves are essentially invisible. This is partly because there is usually extensive overlap between the grey lines and other lines. This may be solved by using a more vivid colour than grey for the ACF curves, or perhaps the ACF lines could be made thicker but with some transparency so that overlapping data can be seen. In any event this aspect of the presentation needs to be improved.
We have made the ACF lines thicker to distinguish from the model fit.

In Figs 2D and 2I measurements of statistical significance between the proportion of protein in fast and slow modes need to be added.
We have included in the revision statistical test comparing the proportions in fast/slow between NLS::eGFP and Bcd::eGFP. Within each dataset, proportions were significantly different from 50/50, except for Bcd::eGFP anterior nuclei.

Relevant to line 174 and Fig 2, NLS should be defined when first used, the source of the NLS should be given (is it from Bcd?) and the rationale for looking at eGFP::NLS should be made explicit.
We have added details on how the eGFP::NLS is generated in the Methods.

In Fig 3D the dashed lines need to be defined. I assume these are experimental error bars but this is not stated.

© 2024. Published by The Company of Biologists under the terms of the Creative Commons Attribution License (https://creativecommons.org/licenses/by/4.0/).

We have reworked Figure 3 based on reviewer comments and have addressed data presentation.

On lines 344-5, shouldn't this conclusion concern the HD rather than the NLS?
Yes, thanks for pointing this out. We have removed this statement.

On line 432, CAP is not an acronym, the correct term is 5' 'cap' or 'cap structure'. Also Cho et al. PMID 15882623 should be added to the references here.
We have changed the corresponding section and added the reference.

On lines 446, 456, 469, and throughout: replace 'blastocyst' with 'blastoderm'. The former term is generally used for embryos that undergo full cellular divisions and cleavage in early embryogenesis, not for syncytial embryos such as Drosophila.
We have changed blastocyst to blastoderm throughout the manuscript.

Reviewer #3 (Significance (Required)):

General assessment: The main strength of the paper is that it extends our knowledge about the dynamics of Bcd gradient formation, and by so doing it advances our understanding of the physical parameters underlying morphological gradients and patterning. The authors are admirably open about questions that were unanswered by the study, which include identifying molecular interactions that affect Bcd dynamics in the cytoplasm, whether Bcd diffusion is dependent on its concentration, and whether other morphogens form gradients in a similar manner. Answering any of these questions would involve substantial experimental work and it is appropriate to leave these questions for subsequent manuscripts.

Advance: A number of high-profile papers were published on this topic in the late 2000s and early 2010s that reached difficult conclusions, so the nature of the mechanisms underlying Bcd gradient formation have remained controversial and somewhat opaque. While this paper does not provide a definitive answer to all relevant open questions, it nevertheless represents a significant advance toward their resolution.

Audience: This paper will be of interest both to developmental biologists interested in gradients and pattern formation, and to biophysicists interested in physical parameters affect molecular movements. It is fundamental research that does not have an obvious clinical or translational component.
We thank the Reviewer for the constructive comments and also for recognising that a number of our unanswered questions belong in future work.

Reviewer #4 (Evidence, reproducibility and clarity (Required)):

Summary:
Athilingam et al. are interested in understanding how the Bicoid (Bcd) morphogen gradient is formed in the early stages of Drosophila embryonic development. Using fluorescence correlation spectroscopy (FCS), the authors quantify the dynamics of Bcd in nuclei and cytoplasm at anterior and posterior regions of the embryo.
First, they characterize the dynamics of eGFP::Bcd in space and time. Analysing FCS autocorrelation curves at the anterior and posterior regions of the embryo during the interphases of nuclear cycles 12 to 14, they detect differences in Bcd diffusion and are able to infer and distinguish two Bcd populations with slow and fast diffusions. Moreover, these dynamics do not vary between nuclear cycles.
Using the different diffusions of the slow and fast populations, they find a model capable of explaining the formation of the gradient at larger distances and the existence of the Bcd molecules in the posterior, compatible with the SDD model widely accepted in the community.
Lastly, given that Bcd has multiple roles binding to DNA and also to RNA through its homeodomain, mutations affecting DNA/RNA and RNA only binding are used. Using a mutant without the ability to bind to DNA, their determine that the slower diffusion in the nuclei are due to DNA binding. They further confirm this by fusing the homeodomain of Bcd to eGFP::NLS.

Major comments:

© 2024. Published by The Company of Biologists under the terms of the Creative Commons Attribution License (https://creativecommons.org/licenses/by/4.0/).

The averaged autocorrelation curves were fitted to models of diffusion with one and two components. The one-component model was insufficient to reproduce the data and the two-component model seems to fit the data. Have the authors tested models with more than two components? Could it be possible to distinguish more Bcd populations?
While it is possible to fit with further components, it rarely provides useful further insight. In particular, the error in measuring three tau_D's is typically very large. In addition, the improvement in the fit will be marginal, and thus the extra components cannot be justified statistically. Of course, we cannot exclude a third (or more) possible dynamic modes, but within the resolution of our FCS measurements two components with triplet states are in general the maximum that can be accommodated without overfitting.

In Figure 2E, the same concentration of eGFP::NLS is estimated to exist in the cytoplasm and nucleus. Since the NLS should target eGFP to the nucleus, what is the explanation for this observation? Is it possible that the method used to estimate the concentration of molecules is underestimating the concentration in the nucleus or the opposite in the cytoplasm?
This is a good observation. There are two possible explanations. First, the regular division cycles "reset" the nuclear levels. Therefore, differences may not be so large. Second, FCS measurements of concentration can be noisy, as they depend on the very short time scales in the measurement. We now clarify this, lines 176-180.

In the simulation of the SDD model (Figure 3B), simulations at 10 min, 25 min and 120 min are shown. Assuming that 120 min corresponds to early nc14, are simulations at earlier timepoints corresponding to nc12 and nc13 indistinguishable from the profile at 120 min? This demonstration would further support the option to merge the data from all nuclear cycles.
This is a good point. Here, we were primarily focused on showing the time evolution of the model, rather than directly mapping onto experiment. We now provide a more detailed time course of the model predictions, where we show the gradient profile relative to the steady-state (Figure 3E). We believe this makes the message clearer. We do note that the profile reaches an approximate steady-state by n.c. 12 (Little *et al.* PLoS Biology 2011).

The results obtained with the BcdN51A mutant show an increase in diffusion speed, while retaining similar proportions of fast and slow populations. In the slow fraction, a new population is found. Assuming that the BcdN51A molecules cannot bind specifically to DNA due to the mutation, what would this newly found population correspond to? Could the authors explore the possibility of nonspecific binding to DNA? The article would also win by discussing more on this aspect or other options.
This is an interesting question. D_{slow} for anterior nuclei of N51A mutants increases (D_{slow} from ~$0.2\mu m^2/s$ to ~$1.5\ \mu m^2/s$), and the proportion is similar to the slow fraction of WT Bcd in the anterior nuclei (F=50%). The D_{slow} values of bcd^{WT} suggest that $0.2\mu m^2/s$ is a result of DNA binding. For bcd^{N51A}, D_{slow} of $1.5\ \mu m^2/s$ is suggestive of nonspecific interaction of bcd^{N51A} to the DNA. Such a nonspecific interaction is also noticed in the case of NLS::eGFP, where we see a significant amount (D_{slow}~ 1-1.5 $\mu m^2/s$, F=20%) of slow form in the anterior nuclei, likely due to non-specific interaction with the DNA.

It is worth noting that the inactive homeodomain of transcription factor *sex comb reduced* (scr) also interacts non-specifically with DNA at high concentration (Vukojevic *et al.*, PNAS 2010). Non-specific interaction of eGFP fluorophore is also noted to be higher in the nuclei of AT-1 cells that suggest "obstacle-free accessible space" is low in the nuclei (Wachsmuth *et al.*, JMB 2000). Therefore, though we do not understand the specific mechanism, our results for N51 mutants are aligned with previous observations of intra-nuclei dynamics. We discuss this briefly in lines 513-516.

The experimental rational behind the BcdMM reporter needs to be better explained as it is not clear. It was previously shown that the N51A mutation disturbs zygotic hb activation and Caudal gradient formation (see Figure 3 in Niessing et al., 2000). Since N51A already causes a strong phenotype by disturbing hb expression and Cad gradient formation, what is the reasoning being adding extra mutations to this background? Since the mutations in the PEST domain and YIRPYL motif are involved in cad translational repression, it would be more interesting to add them to the R54A mutation and further study the repression of cad? It would also shed light on the unexpected

© 2024. Published by The Company of Biologists under the terms of the Creative Commons Attribution License (https://creativecommons.org/licenses/by/4.0/).

no difference or even decrease in diffusion in the cytoplasm of the R54A mutant which should increase if indeed the cad mRNA binding is being repressed.
Our rationale was to remove more elements of Bcd to see if there was some degree of redundancy – at least in terms of the dynamics.

The Bicoid homeodomain N51A mutation is physiologically known to cause de-repression of caudal and inhibit hunchback expression. Mechanistically, nuclear Bcd activates *hb* transcription. However, in the cytoplasm Bcd interacts with other proteins and forms a complex to de-repress *caudal*. Bcd binds to *caudal* mRNA through its HD at one end of the complex. However, in the other end, other proteins in the complex are bound to the 5'cap region *caudal* mRNA. Our rationale for generating the MM mutation was that the N51A mutation may not be sufficient for Bcd to be released from the protein complex. Therefore, additional mutations to N51A may release Bcd from interactions with either DNA or with other proteins through PEST domain and YIRPYL motif. We have clarified this in the revision (lines 375-377).

Have the authors confirmed that their BcdR54A indeed inhibits cad translation?
We have not tested the eGFP:bcd^{R54A} to inhibit *cad* translation. The mutant is well characterised before (Niessing et al., 2000). We took advantage of this published data and generated this mutant line. We are still in the process of doing the testing all the mutant lines generated with null *bicoid* background.

How many embryos of BcdMM were analysed? The authors should also provide a table with all the values in SI as they have done for all the other reporters.
We have now completed the table of measurements. We have added new measurements from two embryos. We have generated this new data from instrument in an independent lab (Frietsche lab, Oxford). From Supplementary Figure S8 and Supplementary Table S5, the values did not change between the two instruments, suggesting robustness of our data.

The claims with eGFP::NLSBcdHD need to be supported by data from multiple embryos. Even if multiple ACF curves are obtained from one embryo, analysing only one embryo is not sufficient. This would clarify the fact that this reporter seems to be able to reproduce the mobility of Bcd in the nucleus.
We agree and we have collected further data from a different FCS setup. It is tabulated in Supplementary Table S4. Our overall conclusions remain unchanged.

According to the methods, all reporters were expressed in a bcd null background, made with the bcd1 allele. This allele is also known as bcd085 and according to Driever and Nusslein-Volhard, 1988 (PMID: 3383244), this allele only causes an intermediate phenotype. This indicates that a truncated version of the protein probably still exists on the embryo. Do the conclusions obtained here still hold if a truncated version of the Bcd protein exists in addition to their reporters?

We used the bcd^{E1} mutant, a null mutant of bcd. This was used by Gregor *et al.*, Cell 2007 in their generation of the original Bcd::eGFP. We have also recently generated a more complete bcd^{KO} mutation (Huang *et al.*, eLife 2020). Our embryos do not have a clear phenotype that we can relate to the specific null *bcd* background used. If there is concentration dependence in Bcd diffusivity and there is some truncated Bcd, this could impact the dynamics. In future work, we can investigate different alleles, to test whether this affect the Bcd dynamics.

Minor comments:
In line 45: "Morphogens are signalling molecules", the authors should consider removing the word "signalling" since not all morphogens are, especially the one being studied, Bicoid.
In lines 80-81 (and also throughout the text): "We measure the Bcd dynamics at multiple locations along the embryo AP-axis", should be more accurate and changed to anterior and posterior of the embryo. Using "multiple locations along the AP axis" is ambiguous and not exact for what was done.
We have edited these sections in the current manuscript.

Throughout the article, the authors refer multiple times to "modes for/of Bcd transport". Since they or others have not proven that Bcd is being transported, which would involve at least another factor, the authors should replace transport by movement, diffusion or a similar word with which they are comfortable.

© 2024. Published by The Company of Biologists under the terms of the Creative Commons Attribution License (https://creativecommons.org/licenses/by/4.0/).

We have changed transport to movement wherever relevant in the text.

Suggestion: The authors claim that the Bcd gradient is exponential up to 60% of embryo length. Would this information allow a more precise calculation of the gradient decay length in the exponential region than the 80-100µm stated on line 202?
This is an interesting point, but our results suggest that the idea of the decay length is not so applicable in the posterior region. There, the Bcd dynamics are generally quicker, thereby increasing □. Of course, we cannot discount possible spatial variation in degradation. However, in previous work, our Bcd tandem reporter (which is sensitive to large changes in degradation) did not reveal spatial variation in degradation.

In lines 258-259, the sentence "Further, Bcd binds to caudal mRNA, repressing its expression in the cytoplasm" should be improved to clarify the role of Bcd in caudal mRNA translation repression and references should be added. This should also be corrected in the following paragraph.
We have corrected in the revision (lines 315-317).

In line 262, "mutations" should be singular since it corresponds to only one amino acid mutation.
We have corrected this.

Figure 4J needs to be corrected as the fractions of the slow and fast populations do not correspond to what is shown in Table 3. For example, Fslow fraction of AC is ~45% in the figure while it is 36% in Table 3. The problem occurs in all fractions.
We thank the Reviewer for spotting this; there was a mislabelling in the corresponding figure. AN is in the place of AC. We have edited Figure 4J.

In the discussion, in lines 379-380, "Given the changing fractions of the fast and slow populations in space, the interactions between the populations are likely non-linear". What is the reasoning for non-linearity and not interchangeability?
If the interactions between the two populations were linear, then the fraction in each form would be constant across the embryo. Some degree of nonlinearity is required in order to have spatially varying relative populations. This related to points raised by Reviewers 1 and 2, and we have updated the manuscript.

In line 432 caudal should be italicized.
We have edited this.

In the discussion, the authors conclude that "In the nucleus, the two populations can be largely (though not completely) explained by Bcd binding to DNA". The discussion would win by explaining all the possible options.
We have improved the Discussion (lines 479-485).

Reviewer #4 (Significance (Required)):

The results presented in this article advance the knowledge of the field by adding data and quantifications of Bcd mobility at four locations: anterior nucleus, posterior nucleus, anterior cytoplasm and posterior cytoplasm. Until now, FCS studies have focused mostly on measuring the dynamics of Bcd in nuclei at the anterior (Abu-Arish et al. 2010; Porcher et al. 2010) of the embryo. The results are also consistent with what was previously found for eGFP:Bcd in the anterior nucleus. Still, this is not surprising as they use the same reporter as the previous studies.
This article will be interesting to an audience comprising biologists and biophysicists interested in protein diffusion.
As a biologist, I do not have sufficient expertise to completely evaluate if the modelling is performed flawlessly. However, in my understanding, the FCS analysis is crucial for the results and their conclusions, hence the comment on the certainty of the existence of only two Bcd populations, though these populations being previously described with FCS. Comments from a physicist with experience in analysing FCS data are thus necessary.

© 2024. Published by The Company of Biologists under the terms of the Creative Commons Attribution License (https://creativecommons.org/licenses/by/4.0/).

Second decision letter

MS ID#: DEVELOP/2023/202128

MS TITLE: Long-ranged formation of the Bicoid gradient requires multiple dynamic modes that spatially vary across the embryo

AUTHORS: Thamarailingam Athilingam, Ashwin VS Nelanuthala, Catriona Breen, Thorsten Wohland, and Timothy Saunders
ARTICLE TYPE: Research Article

Thank you for sending your manuscript to Development through Review Commons.

I am happy to tell you that your manuscript has been accepted for publication in Development, pending our standard ethics checks.

Reviewer 1

Advance summary and potential significance to field

In their revised manuscript, the authors have satisfactorily addressed most of the reviewers' comments.

Comments for the author

It would be useful if the authors could add the missing data for "AN" around 0.1 ms lag time in Fig. 1B.
The presentation of the controversies in morphogen diffusion has improved for Bcd and Dpp, but from the cited Nodal references (Lord et al., 2021; Muller et al., 2012; Wang et al., 2016) that have reported or used similarly high diffusion constants (30-60 um2s-1), it is still not clear to the reader what the contention is.
The authors could also consider adding the newly discovered small Bcd variant as a candidate for the more diffusive fraction (doi:10.1101/2023.12.08.570753) since this hypothesis would not require the switching assumption and could explain the spatial variation of the relative fractions across the embryo.

Reviewer 2

Advance summary and potential significance to field

This manuscript reports a very thorough and careful study of the mobility of Bicoid in the early embryo explored with single-point fluorescence correlation spectroscopy. Although previous groups have looked into this question in the past, the work presented here has more breadth, and is novel and interesting because of the different Bicoid mutants and constructs the authors have examined, in particular with the goal of understanding the role of the protein DNA-binding homeodomain. The authors convincingly show that Bcd exhibits at least two dynamics modes, that there is a significant increase in Bcd dynamics from the anterior to the posterior region of the embryo, and that the homeodomain plays an important role in regulating the Bcd's dynamics. The authors modelled gradient formation and show that this change in dynamics play a small but potentially important role in setting the shape of the gradient. In conclusion, this paper makes a significant advance in our understanding of Bicoid's behavior in the early embryo, and will have an impact on our understanding of morphogen dynamics and gradient formation.

Comments for the author

The authors have satisfactorily answered all my previous comments, as well as that of the other reviewers and significantly improved the clarity of the manuscript and the discussion of their results.

© 2024. Published by The Company of Biologists under the terms of the Creative Commons Attribution License (https://creativecommons.org/licenses/by/4.0/).
